# Superstructure and Correlated Na^+^ Hopping in a Layered Mg-Substituted Sodium Manganate Battery Cathode are Driven by Local Electroneutrality

**DOI:** 10.1021/acs.chemmater.3c02180

**Published:** 2023-12-07

**Authors:** Euan N. Bassey, Ieuan D. Seymour, Joshua D. Bocarsly, David A. Keen, Guido Pintacuda, Clare P. Grey

**Affiliations:** † Yusuf Hamied Department of Chemistry, University of Cambridge, Lensfield Road, Cambridge CB2 1EW, U.K.; ‡ Department of Materials, Imperial College London, South Kensington Campus, London SW7 2AZ, U.K.; § ISIS Facility, STFC Rutherford Appleton Laboratory, Harwell Oxford Campus, Didcot OX11 0QX, U.K.; ∥ Centre de RMN à Très Hauts Champs, UMR 5082 (CNRS/Université Claude Bernard Lyon 1/Ecole Normale Supérieure de Lyon), University of Lyon, 69100 Villeurbanne, France

## Abstract

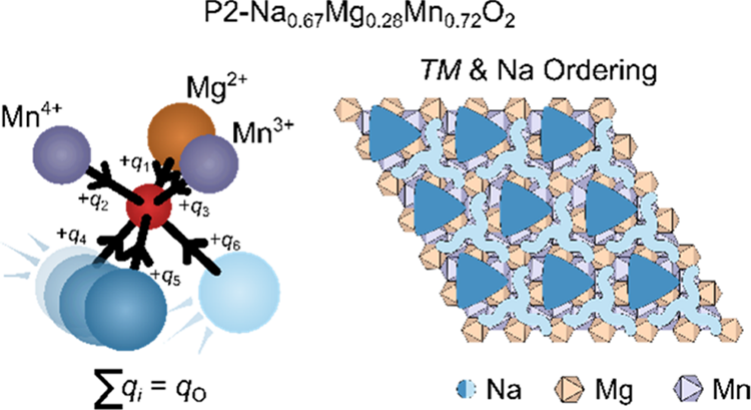

In this work, we present a variable-temperature ^23^Na NMR and variable-temperature and variable-frequency electron paramagnetic resonance (EPR) analysis of the local structure of a layered P2 Na-ion battery cathode material, Na_0.67_[Mg_0.28_Mn_0.72_]O_2_ (NMMO). For the first time, we elucidate the superstructure in this material by using synchrotron X-ray diffraction and total neutron scattering and show that this superstructure is consistent with NMR and EPR spectra. To complement our experimental data, we carry out *ab initio* calculations of the quadrupolar and hyperfine ^23^Na NMR shifts, the Na^+^ ion hopping energy barriers, and the EPR *g*-tensors. We also describe an in-house simulation script for modeling the effects of ionic mobility on variable-temperature NMR spectra and use our simulations to interpret the experimental spectra, available upon request. We find long-zigzag-type Na ordering with two different types of Na sites, one with high mobility and the other with low mobility, and reconcile the tendency toward Na^+^/vacancy ordering to the preservation of local electroneutrality. The combined magnetic resonance methodology for studying local paramagnetic environments from the perspective of electron and nuclear spins will be useful for examining the local structures of materials for devices.

## Introduction

The development of cheap energy storage systems with long lifetimes and high capacities for grid-scale applications is critical to mitigating the effects that arise from the mismatch between the use and production of electricity and modern renewable energy sources. In this regard, sodium-ion batteries (NIBs) are ideally suited, owing to their sustainability and low production costs.^1−4^ If NIBs are to be used on the grid, their capacities, lifetimes, and rate behaviors must, however, be optimized and their degradation and failure mechanisms understood.

At present, the electrochemical performance, and in particular the capacity of a NIB is limited primarily by the cathode material. Understanding how the structures of these cathode materials evolve during cycling—ideally in a noninvasive manner, such that metastable states can be probed without damaging the structure—is critical to developing new cathode materials with high capacities, long lifetimes, and fast rate capabilities.

Layered sodium transition metal oxide materials, Na_*x*_*TM*O_2_ (*TM* = transition metal), similar to their related Li analogues, represent an important class of NIB cathode materials.^5^ As for the Li-containing materials, the structures of these materials may be described using the notation developed by Delmas et al.,^6^ which uses a letter to denote the coordination environment of the alkali metal cation—in NIB cathodes, this is either P for prismatic or O for octahedral—and a number to indicate the number of *TM*O_2_ layers per unit cell. Many problems remain to be solved before layered sodium transition metal oxide materials, Na_*x*_*TM*O_2_, progress to widespread adoption, one of which is the large number of phase transformations that they undergo during charge and discharge, leading to poorer power performance and capacity retention problems.^5^

**Figure 1:**
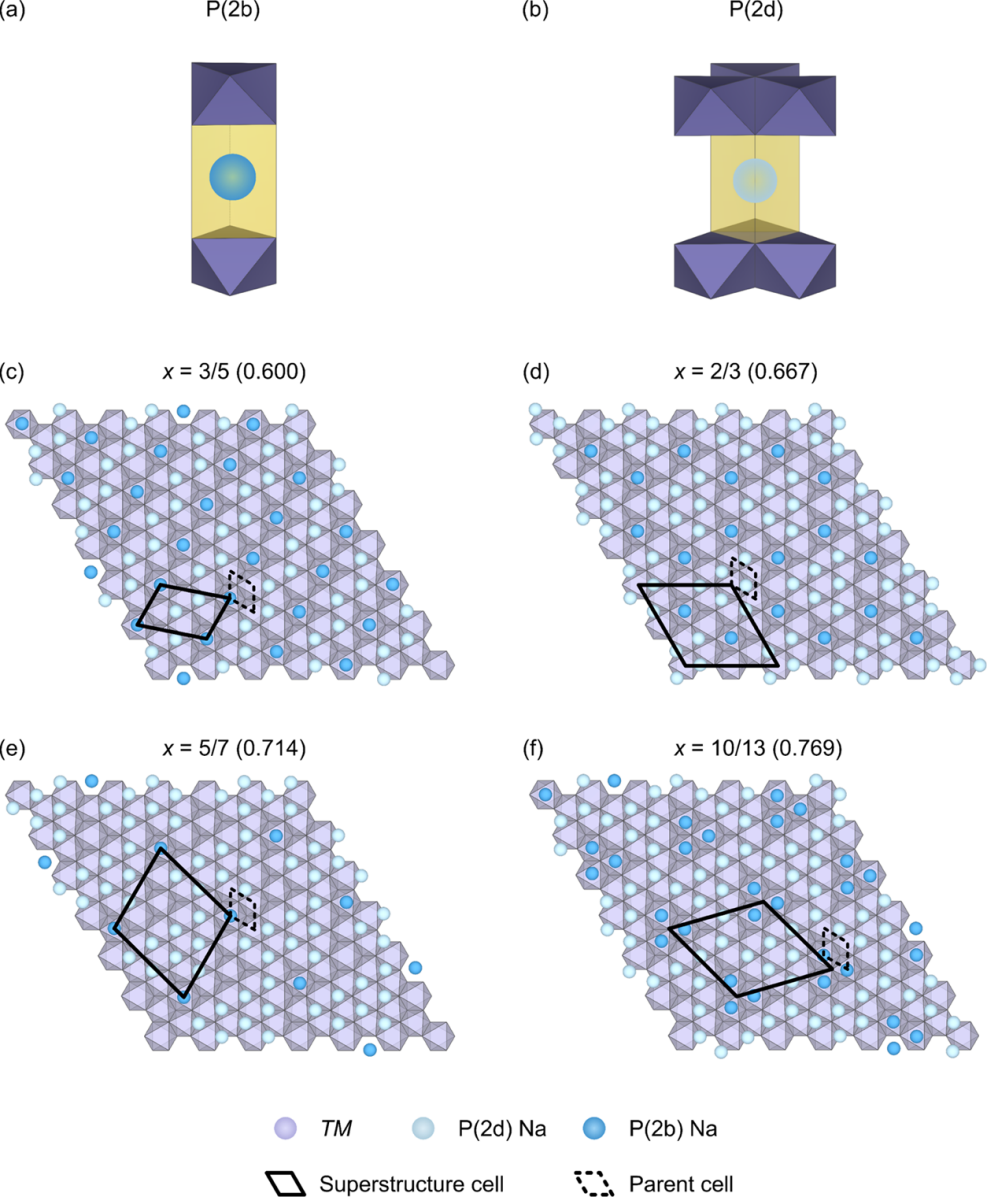
Illustrations of the (a) P(2b) and (b) P(2d) sites in P-type Na_*x*_*TM*O_2_ cathodes and the possible Na^+^ ion/vacancy ordering schemes, as proposed by Hinuma, Meng, and Ceder in refs (9) and (10): (c) *x* = 3/5, corresponding to “row” ordering; (d) *x* = 2/3 and (e) *x* = 5/7, corresponding to long zigzag ordering; and (f) *x* = 10/13, corresponding to droplet ordering. The black outline indicates the unit cell of the superstructure, while the dashed line shows the original parent unit cell (i.e., without Na^+^/vacancy order).

Arguably one of the most promising classes of cathode materials is the P2-type cathodes, where Na^+^ ions occupy prismatically coordinated sites, known as either P(2b) sites—where the Na^+^ prisms share faces with the *TM* octahedra—or P(2d) sites—where the edges of the Na^+^ prismatic coordination polyhedra share edges with the *TM* octahedra in the *TM*O_2_ layers above and below (Figure 1a,b, respectively). Within the Na layer, the large, open faces through which Na^+^ ions in P2-type cathodes hop enable fast charge–discharge rates in these materials when cycled in a NIB. These rates, however, may be severely impacted by Na^+^/vacancy ordering phase transitions, where the development of ordered arrays of Na^+^ and vacancies increases the barrier to Na^+^ hopping,^6,8−10^ resulting in a slower rate of Na^+^ extraction and insertion. Such ordering transformations are electrostatically driven and more prevalent in NIB cathodes than in their Li^+^ ion counterparts (although ordering in Li materials is seen, such as in Li_*x*_CoO_2_ when *x* = 0.5).^11,12^ The origins of this are the greater ionicity (i.e., greater charge separation and weaker covalent interactions) and size of Na^+^ ions compared to Li^+^, resulting in strong Na^+^–Na^+^ and Na^+^–*TM* Coulombic interactions and increased steric hindrance to hopping.^7^ Such transitions may also be only partially reversible, resulting in losses in the active material, or require that large overpotentials are applied to overcome the barrier to Na^+^ extraction (and to move phase boundaries between ordered and disordered regions through the solid^13^); all are deleterious to the electrochemical performance of the cathode. Therefore, understanding and removing these ordering processes are important if we are to develop new cathodes with good performances.

The ordering pattern adopted by Na^+^ ions depends intimately on the Na^+^ content in the cathode (*x* in Na_*x*_*TM*O_2_); in P-type materials, known ordering schemes include row ordering at *x* = 0.5 and *x* = 3/5, where P(2b) Na^+^ are arranged in straight rows separated by small zigzag rows of P(2d) centers; long zigzag (LZZ, a term coined by Hinuma, Meng, and Ceder^9,10^), at *x* = 2/3 and *x* = 5/7, where P(2b) Na^+^ surround “islands” of P(2d) Na^+^ ions (note that if lines connected the P(2b) centers, one could describe the hexagonal set of lines as zigzags; unlike the row ordering, these zigzags span a larger portion of the unit cell); and “droplet” ordering above *x* = 0.75 (a term also coined by Hinuma, Meng, and Ceder^9,10^), where islands of P(2b) Na^+^ are surrounded by “pools” of P(2d) Na^+^ (Figure 1).

In addition to the Na^+^ content, the presence of Jahn–Teller (JT) active *TM* ions (either present in the pristine material or created on cycling) can encourage Na^+^/vacancy ordering, for example, in Na_5/8_MnO_2_, where Na^+^ ions and their vacancies charge-order with the JT-distorted Mn^3+^ centers. In these systems, it was argued that the JT distortion, rather than electrostatics, drives Na^+^/vacancy ordering.^14^ As such, several studies have sought to minimize the number of JT centers present in the pristine material and/or during cycling, most commonly achieved in Na_*x*_MnO_2_-based materials by doping low-valent cations into the *TM* sublattice and raising the average oxidation state of Mn.

While, in many cases, dopants occupy the *TM* sites randomly, some NIB cathode compositions exhibit ordering on the *TM* sublattice, resulting in a superstructure.^15^ This in turn influences the Na^+^ ion ordering patterns.^18,19^ In our earlier work on Na_0.67_[Mg_0.28_Mn_0.72_]O_2_ (henceforth NMMO),^20^ we showed that the Mg and Mn were partially ordered across the *TM* layers. This material showed a surprisingly simple (considering the partial disordering on the Mg/Mn sublattice^21^) ^23^Na NMR spectrum comprising three peaks. Using hybrid density functional theory (DFT) calculations and a simplistic statistical averaging model, we attempted to reproduce the observed spectrum. We noted in that work the strong dependency of Na^+^ ion mobility on the state of charge (SOC) and the need for more complex models to accurately describe the effect of motion on the (variable-temperature) spectra. A similar method was also employed by Lin et al.^22^ to examine the related material P2–Na_2/3_[Mg_1/3_Mn_2/3_]O_2_. This description faithfully models these complex NMR spectra at fast Na-ion exchange rates, paving the way for future studies seeking to model paramagnetic NMR spectra in the presence of ion hopping as a means to extract useful information about ion dynamics in paramagnetic NIB cathode materials. These models do not, however, model all regimes of Na^+^ ion motion: they can capture only the high-temperature, dynamically averaged (fast-exchanged) regime.

In this work, we use synchrotron X-ray diffraction (PXRD), powder neutron diffraction (PND), and neutron pair distribution function (PDF) analysis on pristine NMMO to establish the superstructure. Variable-temperature X-band electron paramagnetic resonance (EPR) is used to examine the local (super)structure of NMMO from the perspective of the unpaired electron spins. The effect of this superstructure is examined in the context of Na^+^ ion mobility by using variable-temperature ^23^Na NMR. These spectra are accurately simulated by solving the Bloch–McConnell equations (see Theoretical Background) for a set of independent two-site exchanges. These simulations enable the assignment of the NMR spectra to individual local Na^+^ environments and provide estimates of the Na^+^ ion hopping barriers, which are compared to *ab initio* barriers determined from the climbing image nudged elastic band (CI-NEB) and against qualitative observations from molecular dynamics (MD) simulations. Our approach is unique in its ability to simulate fast-, intermediate-, and slow-motion regimes, making it applicable to all states of charge and discharge.

## Background Theory

### Theoretical Background: Hyperfine Shifts

The NMR spectrum of paramagnetic materials is generally dominated by the hyperfine interaction between an unpaired electron spin and nearby nuclear spins via through-space (dipolar) and through-bond (Fermi contact) components.^22^ The effects of this interaction are shortened nuclear longitudinal and transverse relaxation times, *T*_1_ and *T*_2_, respectively, in addition to large magnitude isotropic shifts, where the latter is usually dominated by Fermi contact interactions.^23−25^

Previous studies have shown that the hyperfine shift, δ_hyp_, of the nucleus of interest (here, ^23^Na) may be determined by calculating the shifts induced by a single “bond pathway” between a nucleus and the unpaired spin (here, Na^+^–O–*TM*) and summing the bond pathway shifts, δ_hyp_, for a given ^23^Na environment, i.e.,
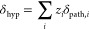
1where *z*_*i*_ is the number of paths with shift δ_path,*i*_, and the sum is taken over all paths between the Na^+^ ion and the *TM* cations in the material.^26−28^ In practice, this sum is computed only over nearest and next-nearest neighbors, as *TM* spins in outer coordination spheres make negligible contributions to the observed Fermi contact shift. We note that while Mg^2+^ is not a *TM* cation, we denote the sites occupied by both Mg and Mn as *TM* sites for the sake of clarity.

The layered P2 cathode has two crystallographic Na^+^ sites: P(2d) and P(2b) (Figure 1a), but due to atomic site disorder, Na ions on a single crystallographic site can have different local environments (i.e., a different number of nearby *TM* cations with different Na^+^–O–*TM* bond angles and distances), resulting in an NMR spectrum with more than just two resonances.^23,25^ In the NMMO, three species can occupy the *TM* layer: Mn^3+^, Mn^4+^, or Mg^2+^. All P(2d) sites have six nearest neighbor *TM* cations and six next-nearest neighbor*s*; all P(2b) sites have two nearest neighbors and 12 next-nearest neighbors. If a “random” distribution of Mn^3+^, Mn^4+^, and Mg^2+^ is assumed, 1144 unique Na^+^ environments occur (784 P(2d) and 360 P(2b)); this number significantly increases if more distant shells are considered.

In our previous work, we used a model system, Na_2/3_[Mg_1/3_Mn_2/3_]O_2_, to calculate the hyperfine bond pathways, as well as the quadrupole-induced shifts.^24^ We note that Mg contributes a negligible amount to the total shift of Na, as Mg^2+^ is diamagnetic. Attempts to calculate bond pathways involving Mn^3+^—either by adding a single Mn^3+^ center or adding multiple Mn^3+^ centers (and charge compensating each by adding Na^+^ ions) or by doping a polaron into the system (making the cell charged)—were unsuccessful. Despite this, the bond pathways calculated for this model represent a good starting point with which to model the ^23^Na NMR shifts of NMMO (considering that the “true” composition is Na_0.67_[Mg_0.28_Mn_0.11_^3+^Mn_0.61_^4+^]O_2_).

Previous attempts to model the NMR spectra using a random arrangement of Mg^2+^ and Mn^4+^ ions (a total of 88 unique local Na^+^ environments) or to model a system with a honeycomb-ordered arrangement of Mg^2+^ and Mn^4+^ ions generated spectra inconsistent with experimental spectra. Each of these models, however, assumed a static arrangement of Na^+^ ions, i.e., Na^+^ ions did not hop between sites during the NMR experiment. As observed in several layered NIB and lithium-ion battery (LIB) cathodes,^24,28−30^ the effect of alkali ion hopping on the NMR time scale results in significant changes to the appearance of the spectrum.

The form of the NMR spectrum in the presence of Na^+^ ion motion (more generally known as chemical exchange) depends primarily on the difference in shift between the sites, Δ*ω*, and the hopping rate, *k*.^31^ Three regimes exist: (1) the fast-hopping regime, where Na^+^ ions hop at a rate that is faster than the difference in the sites’ resonant frequencies (i.e., *k* ≫ Δ*ω*); (2) the intermediate regime, where Na^+^ ions hop at a rate which is comparable to the difference in resonant frequencies (i.e., *k* ∼ Δ*ω*), and (3) the slow hopping regime, where Na^+^ ions infrequently hop between sites compared to the frequency difference (i.e., *k* ≪ Δ*ω*). In the fast-hopping regime, a sharp resonance is observed at a frequency that is the population-weighted average of the sites’ frequencies; the intermediate regime results in resonances that are broad, while the slow regime is characterized by two (or more) distinct resonances at the resonant frequencies of the different sites (Figure S1).

In our earlier work, we modeled the NMMO spectra by assuming a population-weighted average of the Na^+^ sites (in a fashion similar to Lin et al.^22^), using an exchange equilibrium constant, *K*, to obtain the ^23^Na NMR shifts for a Na ion hopping between a P(2d) and P(2b) site.^25^ This approach is appropriate for the fast regime but does not capture the behavior of the intermediate or slow regimes seen in NMMO. In this work, we construct a model that accounts for all three regimes and simulate the variable-temperature ^23^Na NMR spectra of NMMO using this model.

## Experimental Section

### Synthesis

The synthetic route used was based on previous reports (see the Supporting Information (SI), Section S1 for further details).^16,32^ Throughout this work, all samples were handled and prepared in an Ar-filled glovebox (H_2_O and O_2_ < 1 ppm).

### Powder Synchrotron X-ray Diffraction

A powder sample of NMMO was sealed in a borosilicate glass capillary by using two-component epoxy resin inside the glovebox. Powder synchrotron X-ray diffraction (PXRD) patterns were acquired on beamline I11 at the Diamond Light Source.^33,34^ The pattern was collected with an exposure time of 1 min using a position-sensitive detector (PSD, Mythen2; X-ray wavelength = 0.826866 Å) over the range 2θ = 2.0–92°. Variable-temperature PXRD patterns were also recorded on heating from 100 to 500 K (heating rate 3 K min^–1^), with an exposure time of 12 s per pattern (i.e., 0.6 K temperature difference between the start and end of acquisition), using the same PSD and 2θ range.

### Powder Total Neutron Scattering

Powder neutron diffraction (PND) measurements were carried out at the ISIS Pulsed Neutron and Muon Source using the GEM instrument.^35^ A powder sample of pristine Na_0.67_[Mg_0.28_Mn_0.72_]O_2_ (3.44 g) was loaded into a thin-walled vanadium canister (8 mm diameter) to a height of 59 mm to maximize scattering from the sample. Total scattering data were initially collected at 130 K, then 1.6 K, followed by a series of diffraction patterns acquired from 2 K up to 7 K in 1 K steps. The complete list of temperature steps and data collections may be found in the SI (Section S4). The data were corrected for absorption effects using the Mantid software package^36^ to provide data suitable for Rietveld refinement. To normalize the total scattering data, additional measurements of the empty vanadium canister, empty cryostat, and vanadium rod were taken and all data were processed within the GudrunN software to produce absolute total scattering structure functions, *F*(*Q*). The *F*(*Q*) were then Fourier transformed to provide pair distribution functions, PDF, or *D*(*r*), as defined in ref (37).

The structure was determined by Rietveld refinement^37,38^ against powder neutron diffraction data collected above the Néel temperature, *T*_N_, using a model derived from the previously reported powder diffraction structure of Na_0.67_[Mg_0.28_Mn_0.72_]O_2_.^16^ All refinements were carried out using TOPAS Academic 6.0.^39^

### ISODISTORT and Refining Superstructures

Candidate superstructures were generated using the software package ISODISTORT, part of the ISOTROPY suite.^40^ Occupancies and displacements of ions in the structure are calculated based on the irreducible representations of the parent structure space group, which are consistent with the ordering scheme provided (where the ordering scheme may be deduced from the location of superlattice reflections). The standalone packages ISOVIS and ISOVISQ are especially useful for identifying symmetry-compatible distortion modes, as well as for visualizing the effects of distortions on the structure and diffraction pattern.

Rietveld refinements of the superstructure using the candidate Na^+^ ion occupation and Mg/Mn occupation irreducible representations (irreps) showed that the *M*_2_^+^ irrep structures were consistent with the experimental data. On this basis, the superstructure was refined using the P3 mode of the *M*_2_^+^ irrep. Here, P3 is distinct from the labels for cathode structures, the P here denoting a one-dimensional order parameter direction, and three an arbitrary label (i.e., the third such one-dimensional order parameter direction). All refinements were carried out by simultaneously refining against the pair distribution function data (obtained from a Fourier transform of the total scattering data in GudrunN), the diffraction data collected on banks 2 to 5, and the PXRD data collected.

For refinement against the PND and laboratory XRD data, the background was fit with a 12-term Chebyshev polynomial. The lattice parameters and atomic positions were allowed to refine freely, aside from restraints on the Mn–O (*ca*. 1.85 Å) and Mg–O (*ca* = 2.00 Å) bond lengths. Separate sets of isotropic atomic displacement parameters were freely refined for Na, Mg, Mn, and O atoms, while the occupancies of Na, Mg, and Mn were refined using the symmetry modes of the irrep and under the constraint that the sample composition was Na_0.67_[Mg_0.28_Mn_0.72_]O_2_.

### Electrochemistry

Electrodes of NMMO were prepared as described in the SI. All electrochemical tests were conducted using NMMO/Na metal half-cells in 2032 stainless-steel coin cells. Each cell was assembled from a stack of one cathode, one glass fiber separator (Whatman, GF/B, 0.68 mm thick, 16 mm diameter, 1.0 μm pore size) soaked with 150 μL of electrolyte (1.0 M NaPF_6_ in propylene carbonate, PC), and one Na metal disc. The NMMO/Na cells were galvanostatically charged and discharged at a rate of 10 mA g^–1^ (corresponding to approximately *C*/19, for a theoretical *C* rate determined from the time elapsed and current applied, assuming that *x* in Na_*x*_[Mg_0.28_Mn_0.72_]O_2_ varies between 0 and 1 and that no parasitic reactions take place during cycling) over a voltage window of 1.5–4.5 V (vs Na/Na^+^). A slow cycling rate was chosen to minimize the effect of ionic concentration gradients and large overpotentials often seen at higher cycling rates.

### Solid-State Nuclear Magnetic Resonance Spectroscopy

Electrochemically cycled cathodes of NMMO were prepared by cycling a cathode to a given cutoff voltage and allowing the cell to rest for at least 1 h. The cell was opened inside an Ar-filled glovebox, and the cathode was extracted, washed in dimethyl carbonate (DMC; approximately 1 cm^3^; Sigma-Aldrich, 99%, anhydrous), and dried *in vacuo* for at least 20 min. The cathode was then scraped off the Al foil current collector and either packed into a 1.3 mm diameter ZrO_2_ magic angle spinning (MAS) rotor or center-packed into a 2.5 mm diameter ZrO_2_ MAS rotor, using poly(tetrafluoroethylene) (PTFE) tape to fill the gap at either end of the rotor and Vespel caps at each end. No rotor spent longer than 10 min outside of the glovebox before being inserted into the magnet under a protective atmosphere of flushing nitrogen gas.

The majority of the ^23^Na NMR spectra were recorded on a Bruker Avance III 11.7 T spectrometer by using a Bruker 2.5 mm MAS probe with an MAS frequency of 28 kHz or a Bruker 1.3 mm MAS probe at an MAS rate between 30 and 60 kHz. In all cases, a rotor-synchronized Hahn-echo pulse sequence was used for quantitative measurements, and the recycle delay (25 ms; at least 5*T*_1_) was set such that the bulk, paramagnetically shifted signal was recorded quantitatively, while the diamagnetic signal due to electrolyte decomposition products was suppressed. An effective  pulse length of 1.15 μs (for the 2.5 mm probe) or 0.67 μs (for the 1.3 mm probe) was used. This corresponds to , which is longer than the selective  pulse which would ensure all quadrupolar ^23^Na centers are in the quadrupolar liquid limit;^41^ a compromise was therefore selected between the linear quadrupolar regime and maximizing the signal intensity). The 2.5 mm ZrO_2_ MAS rotor was chosen so that a relatively wide range of temperatures could be accessed; the spinning speed chosen was 28 kHz, as this was sufficient to separate the spinning sideband manifold from its isotropic resonance while ensuring rotor stability. Spectra were also recorded on a Bruker Avance III 16.4 T spectrometer using a Bruker 1.3 mm MAS probe with an MAS frequency of 50 kHz and an effective  pulse length of 0.58 μs (again corresponding to ). Additional experiments were carried out using a Bruker Avance III 9.4 T spectrometer using a Bruker 1.3 mm MAS probe with an MAS frequency of 35 kHz and an effective  pulse length of 1.47 μs (again corresponding to ).

All spectra were referenced to solid NaCl at 7.21 ppm and scaled according to the sample mass and number of residuals recorded.

For all experiments, temperature calibration measurements were performed either *ex situ* using the ^207^Pb shift of Pb(NO_3_)_2_ (Alfa Aesar, 99%) or *in situ* by mixing pristine NMMO powder with KBr (Aldrich, 99% FTIR grade, dried at 473 K for 12 h under dynamic vacuum) and recording the ^79^Br spin–lattice *T*_1_ relaxation time. Calibrations were run under the same heater powers, nitrogen gas flow rates, and drive and bearing pressures used for ^23^Na NMR experiments; the calibration curves are given in the SI (Figures S2 and S3). Using the *in situ* calibration curves obtained at 11.7 T for KBr at probe temperatures between 300 and 325 K and MAS rates between 30 and 50 kHz, sets of probe temperatures were selected at each MAS speed so that, at each speed, the sample temperature remained constant (to within 2 K, corresponding to a change of approximately 15 ppm to the isotropic shifts of NMMO) and only the MAS rate changed.

### Continuous-Wave X-Band Electron Paramagnetic Resonance Spectroscopy

Continuous-wave X-band EPR measurements were performed on a Bruker E500 X-band spectrometer with an ER 4122SHQE cavity tuned to 9.373 GHz. The external magnetic field was modulated at 100 kHz with a modulation amplitude of 0.3 mT. The microwave power was set to 0.6325 mW, sufficient to avoid saturation. The as-synthesized powder (prepared as above) was loaded into a quartz EPR tube (Aldrich, Wilmad CFQ tubes, outer diameter 2 mm) inside an Ar-filled glovebox and then loaded inside an Oxford Instruments ESR900 cryostat with a temperature stability of 0.5 K. The sample was first cooled to base temperature (5 K), and spectra were recorded on heating, with a five-min equilibration period allowed once temperature stability was reached. All EPR spectra were fitted to a powder pattern line shape with isotropic g-tensors using the EasySpin toolbox for MATLAB.^42^

### First-Principles Calculations

Three model systems were examined for calculation of NMR and Na^+^ ion diffusion parameters: first, a (2 × 1 × 2) supercell of honeycomb-ordered P2–Na_2/3_[Mg_1/3_Mn_2/3_]O_2_ with 88 atoms (chosen to capture as many exchange interactions as possible; structures available as part of the SI) for NMR shifts in a Mn^4+^-only system; second, a (2 × 2 × 1) supercell of P2–Na_2/3_[Mg_1/3_Mn_2/3_]O_2_ for Na^+^ ion hopping barriers (chosen to minimize self-interactions between hopping Na^+^ centers), and last a (2 × 2 × 1) supercell of P2–Na[Mg_1/3_Mn_2/3_]O_2_ for NMR calculations in a mixed Mn^3+/4+^ system, in which there are equal number of Mn^3+^ and Mn^4+^ centers. While this composition differs from pristine NMMO, it is assumed that the Mn^3+^ and Mn^4+^ bond pathways are independent and, therefore, additive, based on previous studies.^24,26^

### NMR Shift Calculations

The ^23^Na NMR shifts of different local Na^+^ ion environments were calculated using methods outlined previously.^26−28,43^ An initial geometry optimization was performed using the Vienna *Ab Initio* Software Package (VASP) code (energy tolerance 10^–5^ eV; force tolerance 0.02 eV Å^–1^),^44−46^ employing the projector-augmented wave (PAW) method.^47,48^ Spin-polarized Perdew–Burke–Ernzerhof exchange–correlation functionals were used under the Hubbard *U* model^49−51^ within the rotationally invariant formalism proposed by Liechtenstein et al.^52^ to correct for known deficiencies of pure functionals in highly localized 3*d* states.^52^ A plane-wave energy cutoff of 520 eV was chosen alongside an effective Hubbard *U* parameter on the Mn *d* states of *U*_eff_ = *U* – *J* = 3.9 eV, where *U* and *J* are the effective on-site Coulomb and exchange parameters (*J* = 1 eV), respectively, in line with previous work on the parent material, Na_*x*_MnO_2_.^53^ The Brillouin zone was sampled with a Monkhorst–Pack^54^*k*-point mesh of density <0.5 Å^–1^.

Periodic spin-polarized DFT calculations of the hyperfine and quadrupole-induced shifts were performed in CRYSTAL17.^55^ Hyperfine parameters were calculated with B3LYP^56,57^ and a modified B3LYP hybrid functional containing 20 and 35% Hartree–Fock exchange, referred to as Hyb20 and Hyb35, respectively. These weights were chosen based on the success of these functionals in calculating the properties of *TM* compounds and have been previously reported to provide an upper and lower bound on experimental shifts.^26−28^ Additional computational details, including the number of Gaussian primitives and the contraction scheme used for each basis set, alongside details of convergence criteria used, are provided in the SI (Section S1).

### Climbing Image Nudged Elastic Band Calculations

The energy barriers to Na^+^ ion hopping were determined using the climbing image nudged elastic band (CI-NEB) double-ended transition state searching method implemented in VASP.^58^ In all cases, a geometry optimization of the initial and final states was carried out using PBE functionals, with the same *U*_eff_ parameter and plane-wave energy cutoff as above, but a γ-centered *k*-mesh sampling of <0.2 Å^–1^ and energy tolerance of 10^–7^ eV. To obtain the energy barriers, five linearly interpolated image states were generated between the initial and final states using the VTST packages,^59^ and the forces perpendicular to the path connecting them were minimized to <0.01 eV Å^–1^. Linear interpolation of the forces between these images was then carried out to obtain the energies of each pair of images. The composition of each cell is given in the SI (Section S9).

### Molecular Dynamics Simulations

Molecular dynamics (MD) simulations—used to assess Na^+^ ion mobility—were also carried out in VASP. To ensure that the calculations were affordable, the plane-wave kinetic energy cutoff was lowered to 400 eV and γ-centered *k*-point sampling was kept at <0.5 Å^–1^. The MD simulations were carried out in the NVT ensemble using a Nosé–Hoover thermostat with a time step of 2 fs. To ensure that diffusion could be observed over the time scales used, elevated temperatures were required,^60,61^ with simulations being carried out at a range of temperatures from 400 to 800 K. The systems were equilibrated for a period of 5 ps, and the simulations were then run for at least 160 ps. Final configurations of MD runs were visually inspected and then optimized to ensure that the *TM* framework had not changed. The self-diffusion coefficient, D*, could not be obtained owing to highly correlated Na^+^ ion motion (see Results).

## Results

### Synchrotron X-ray Diffraction, Neutron Diffraction, and Pair Distribution Function Analysis of Cation Ordering

**Figure 2:**
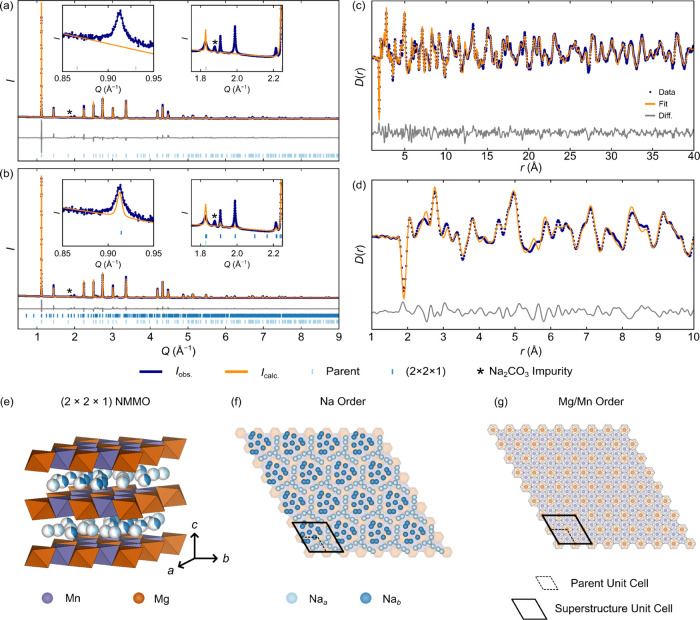
Rietveld refinement of the PXRD pattern collected for pristine NMMO at room temperature against (a) of the parent phase structure only (*R*_wp_ = 11.85%) and (b) of the parent and superstructure phases together (*R*_w.p._ = 3.93%). Insets show the fits in the regions *Q* = 0.85–0.95 Å^–1^ and *Q* = 1.75–2.25 Å^–1^, where superstructure reflections are prevalent. In (c), Rietveld refinements of neutron pair distribution function data recorded at 130 K over the region *r* = 1–40 Å, against, in the same model, both the parent (35 wt %) and superstructure (65 wt %) are shown; (d) shows the same refinement in the region 1–10 Å. In (e), (f), and (g), views of the Rietveld-derived (2 ×  2 × 1) superstructure and expanded view of the ordering in the Na and Mg/Mn layers, respectively, are shown. Note that the Mg and Mn occupancies have been set to be perfectly honeycomb ordered and that all Na occupancies have been set to 1 for clarity. For the true occupancies, please see site occupancies in Table 1.

NMMO was synthesized via a high-temperature solid-state reaction between Na_2_CO_3_, MgO, and Mn_2_O_3_ using the route reported by us^25^ and Maitra et al. Simultaneous Rietveld refinements^16,17^ of the synchrotron X-ray diffraction (PXRD) and powder neutron diffraction (PND) data showed that the majority of reflections could be indexed within the previously reported structure of NMMO (space group *P*6_3_/*mcm*),^16,32^ in which the Mg and Mn centers are randomly distributed across the *TM* sublattice and Na^+^ ions occupy the Na sublattice with a random occupancy (Figure 2a, Table 1). Additional reflections (for example, at *Q* = 0.91, 1.99, 2.21, and 2.83 Å^–1^, among others, see Figure 2 inset) were also present; these have been observed and qualitatively ascribed by several authors (studying both NMMO and other P2 NIB cathode materials)^16,62^ to ordering on the *TM* and/or Na sublattices, but the nature of the ordering was not determined in these previous studies. The low-intensity reflection at *Q* = 1.88 Å^–1^ was assigned to a Na_2_CO_3_ impurity phase (consistent with ^23^Na NMR; see later).

**Table 1:** Rietveld-Derived Lattice Parameters, Atomic Coordinates and Occupancies for the Parent Structure of P2-NMMO at Room Temperature, *R*_w.p._ 9.00%, and the (2 × 2 × 1) Superstructure, *R*_w.p._ 3.93%^a^

**Parent**						
space group		*P*6_3_/*mcm*				
***a*****(Å)**	5.02634(2)		***α*****(deg)**	90*		
***b*****(Å)**	5.02634(2)		***β*****(deg)**	90*		
***c*****(Å)**	11.1929(10)		***γ*****(deg)**	120*		
	**Site**	***x***	***y***	***z***	**Occupancy**	***B***_**eq**_**(Å**^**2**^**)**
Mg1	2*b*	0	0	0	0.49(2)	0.51(10)
Mn1	2*b*	0	0	0	0.51(2)	0.51(10)
Mg2	4*d*	1/3	2/3	0	0.174(2)	0.51(10)
Mn2	4*d*	1/3	2/3	0	0.826(2)	0.51(10)
O	12*k*	1/3	1/3	0.0880(3)	1*	0.57(10)
Na1	6*g* (P(2d))	0.301*	0	1/4	0.413(6)	0.26(6)
Na2	4*c* (P(2b))	1/3	2/3	1/4	0.386(6)	0.26(6)
						
**(2 × 2 × 1)**						
space group		*P*6_3_/*m*				
***a*****(Å)**	10.05266(5)		***α*****(deg)**	90*		
***b*****(Å)**	10.05266(5)		***β*****(deg)**	90*		
***c*****(Å)**	11.1929(10)		***γ*****(deg)**	120*		
	**Site**	***x***	***y***	***z***	**Occupancy**	***B***_**eq**_**(Å**^**2**^**)**
Mg1_1	2*b*	0	0	0	0.82(5)	0.51(10)
Mg1_2	6*g*	1/2	0	0	0.82(5)	0.51(10)
Mn1_1	2*b*	0	0	0	0.18(5)	0.51(10)
Mn1_2	6*g*	1/2	0	0	0.18(5)	0.51(10)
Mg2_1	12*i*	1/3	1/3	0	0.02(3)	0.51(10)
Mg2_2	4*f*	1/3	2/3	1/2	0.02(3)	0.51(10)
Mn2_1	12*i*	1/6	1/3	0	0.98(3)	0.51(10)
Mn2_2	4*f*	1/3	2/3	1/2	0.98(3)	0.51(10)
O1_1	12*i*	0.1529(5)	0	0.5962(7)	1*	0.57(10)
O1_2	12*i*	0.1529(5)	1/2	0.5962(7)	1*	0.57(10)
O1_3	12*i*	0.6529(5)	0*	0.5962(7)	1*	0.57(10)
O1_4	12*i*	0.6529(5)	1/2	0.5962(7)	1*	0.57(10)
Na1_1	6*h* (P(2d), Na_*a*_)	0.8648(12)	0.0227(12)	1/4	0.40(4)	0.26(6)
Na1_2	6*h* (P(2d), Na_*a*_)	0.8031(12)	1/2	1/4	0.53(14)	0.26(6)
Na1_3	6*h* (P(2d), Na_*b*_)	0.3421(12)	0.9773(12)	1/4	0.40(4)	0.26(6)
Na1_4	6*h* (P(2d), Na_*b*_)	0.4039(12)	1/2	1/4	0.26(13)	0.26(6)
Na2_1	6*h* (P(2b), Na_*b*_)	0.1348(4)	0.3652(4)	1/4	0.41(5)	0.26(6)
Na2_2	2*d* (P(2b), Na_*a*_)	2/3	1/3	1/4	0.41(5)	0.26(6)
Na2_3	6*h* (P(2b), Na_*a*_)	0.8970(4)	0.6985(4)	1/4	0.41(5)	0.26(6)
Na2_4	2*c* (P(2b), Na_*b*_)	1/3	2/3	1/4	0.41(5)	0.26(6)

a Standard Errors are shown in parentheses, while asterisks denote values that were not refined. The coordination of the Na^+^ site (P(2d) or P(2b)) and designated name in the superstructure (Na_*a*_ or Na_*b*_) is indicated next to the Wyckoff site label.

Further refinements of the PXRD and PND data to identify any potential superstructures proved challenging, as the additional reflections could be indexed to both (2 × 2 × 1) and (√3 × √3 × 1) ordering vectors. To test possible ordering schemes, several hundred candidate structures based on these vectors with different combinations of symmetry distortions of Na and/or O displacements, as well as Na, Mg, and Mn occupancies, were generated using the ISODISTORT software package.^40^ Careful consideration of the low-*Q* reflection at *Q* = 0.91 Å^–1^ and the maximal nonisomorphic subgroups of the parent *P*6_3_/*mcm* space group indicated that only a handful of space groups allowed this low-*Q* reflection (see the Supporting Information, SI). Of these, the best fit was obtained with both the parent structure (35 wt %) and the P3 mode under the *M*_2_^+^ irrep. (i.e., a (2 × 2 × 1) expansion of the parent structure with space group *P*6_3_/*m* indicated by the bold cell in Figure 1d, 65 wt %). This refinement produced a better match to the observed diffraction patterns than refinement to the parent alone, indicating that a superstructure is required to capture the “true” structure of NMMO (Figure 2b). Note that, in the refinements, the Na, Mg, and Mn occupancies were allowed to refine separately (within the constraints dictated by the symmetry mode distortions) such that each Mg/Mn site was fully occupied, and the overall stoichiometry of the sample was preserved.

The superstructure Mg/Mn ordering reflects the ordering seen in the parent material: Mg at the corners, center, and centers of edges of the unit cell, with Mn surrounding them (Figure 2g). This cell, however, has a more pronounced ordering than in the parent cell (see the site occupancies in Table 1). The superstructure generates eight different Na sites—four that are P(2d)- and four P(2b)-like. In Figure 2f, we have relabeled the Na sites as Na_a_ and Na_b_, the two different subgroups, each containing both P(2d) and P(2b) coordination, so as to illustrate the Na-ion ordering and its relationship to the LZZ ordering seen in Na_0.67_CoO_2_, with Na ions either in the “long zigzags” (to use Hinuma, Meng, and Ceder’s description^9,10^) or in the “islands” between the long zigzags. Some of these sites are clearly too close together to be simultaneously occupied, as discussed later.

Refinement of both the (2 × 2 × 1) superstructure and parent structure against, simultaneously, the synchrotron and neutron diffraction and the neutron pair distribution function (PDF) data gave an excellent match, suggesting that it also provides an accurate description of the local structure (Figure 2c,d). We note, however, that the fit to the long-range structure from PXRD and PND is not perfect, with some of the calculated reflections having intensities that are lower than the observed reflections, for example, (200), (21̅0), and (21̅2) at *Q* = 1.44, 1.99, and 2.21 Å^–1^, respectively (Figure 2b inset). To identify possible discrepancies in our structural model that caused these differences, a Fourier difference map was synthesized, which suggested that Mg and Mn partially occupy tetrahedral sites within the *TM* layer. Given the good fit of Mn and Mg *K*-edge EXAFS data published in the work by Boivin et al. and us to octahedral Mg and Mn, tetrahedral Mg/Mn was deemed unlikely.^17,20^ Additional refinements, including domains of O2- or OP4-NMMO, indicated that neither was present in the pristine material.

It therefore appears likely that the mismatched intensities arise from additional displacement or occupational distortion modes; the fit was not improved by incorporating simple preferred orientation models or without introducing a large number of additional degrees of freedom.

Overall, our refinement demonstrated the presence of both Mg/Mn ordering and Na ordering: the Mg and Mn centers show a strong tendency toward honeycomb order, with Mg ions preferentially occupying the center, corners, centers of edges, and faces of the unit cell and Mn centers occupying the remaining sites (Figure 2e,g).

The effect of Na^+^ ion ordering on the local structure is observed in the PDF data, where the peaks between *r* = 2.06 Å and *r* = 2.76 Å correspond to Na–Na distances in the (2 × 2 × 1) supercell; these are not captured by the parent only, as seen in additional refinements in the SI (Figure S7). Note that the total number of Na sites in each Na_a_/Na_b_ group is equal, with a 2:3 ratio of P(2b) and P(2d) sites, respectively (Table 1).

**Figure 3:**
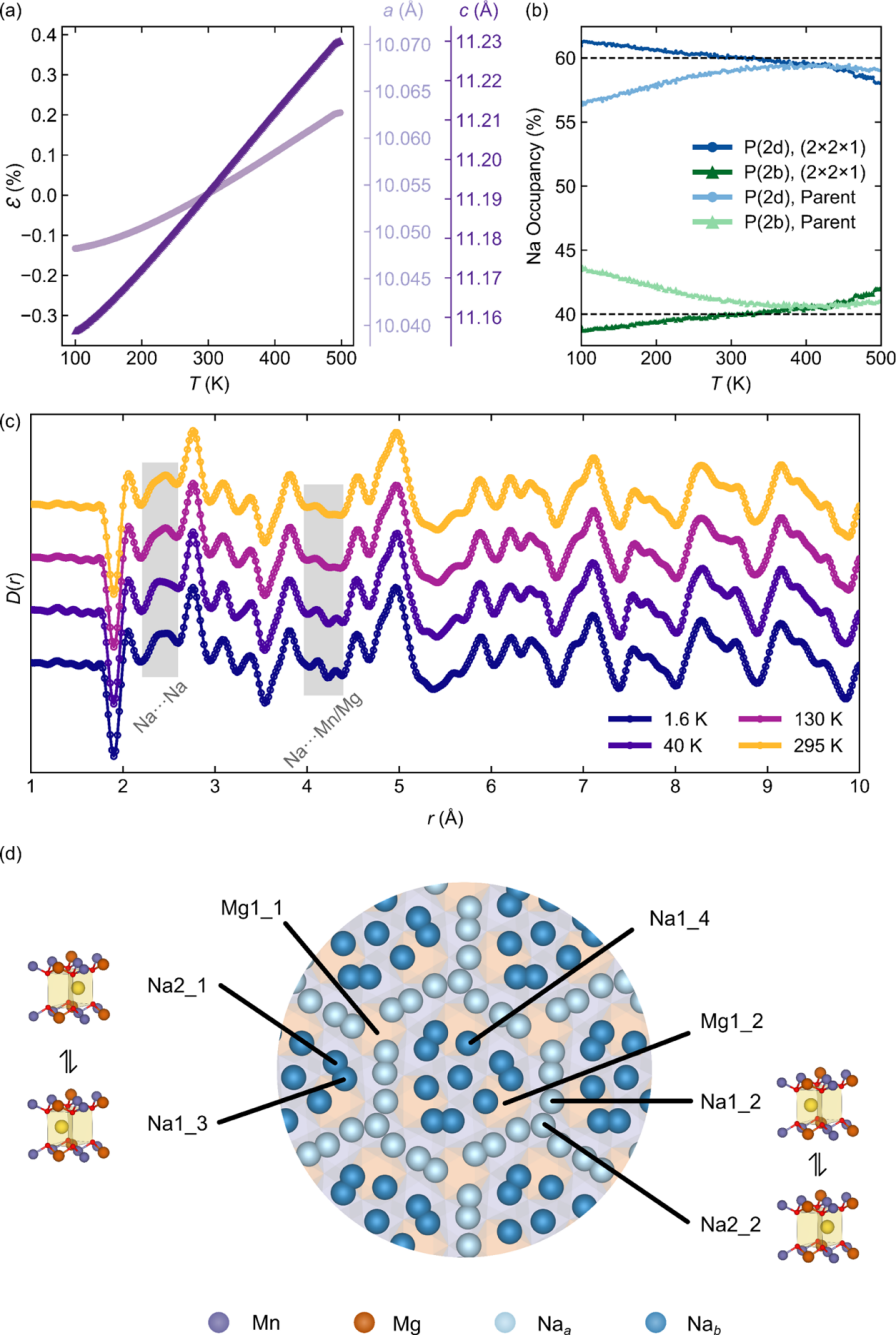
Variable-temperature PXRD and neutron PDF data: (a) shows the expansion, ε, in the lattice parameters, *a* and *c*, of the superstructure, and (b) Na site occupancies for both the parent and (2 × 2  ×  1) superstructure. Note that the *a* lattice parameter for the parent structure is one-half of that for the superstructure, while the *c*-axis is the same in both the parent and superstructures. The expansion is calculated in the following way: ε = (*x*–*x*_295 K_)/*x*_295 K,_ where *x* is either *a* or *c* and *x*_295 K_ is the value of the parameter at 295 K (to compare to lattice parameters under battery operating conditions). The errors for the lattice parameters and occupancies are given by the sizes of the markers. In (c), the variable-temperature neutron PDF data is indicated, with the *r* = 4.1 to 4.35 Å and *r* = 2.1 to 2.6 Å regions highlighted. In (d), an expansion of the Na layer of the (2 × 2  ×  1) superstructure is shown, with the positions of selected Na and Mg centers highlighted to show exemplar Na^+^ ions with short Na^+^–Na^+^ distances that cannot be simultaneously occupied and are likely undergoing rapid exchange.

Variable-temperature PXRD (between 100 and 500 K, on heating) and PND patterns and PDF data (between 1.6 and 295 K, again on heating) were collected (Figure 3) to explore Na mobility and changes in ordering with temperature. As expected, on increasing the sample temperature, the refined *a* and *c* lattice parameters increased, with *c* increasing approximately twice as much as *a* due to the greater flexibility in the *c*-axis (Figure 3a).

The variable-temperature PXRD also revealed changes in Na^+^ site occupancies; in P2-NMMO, the ratio of P(2b) to P(2d) sites is 2:3, and in a truly “random” occupation of the Na^+^ sublattice, this ratio should be reflected in the site occupancies. Thermodynamically, however, the P(2d) sites are anticipated to be lower in energy owing to the lower Coulombic repulsions (between Na^+^ and the Mg and Mn cations in the adjacent *TM*O_2_ layers). The trend observed for the P(2d) (Na1_1 to Na1_4 in Table 1) and P(2b) (Na2_1 to Na2_4 in Table 1) Na^+^ ions in the superstructure follows the expected trend: P(2d) has a higher occupancy at low temperature, and this occupancy decreases on increasing temperature due to thermally activated occupancy of the P(2b) sites (Figure 3b). Refinements carried out with the parent structure do not follow this trend, with occupancies tending to 1:1 at low temperatures (Figure 3b), further suggesting that the parent structure does not accurately capture Na^+^ ion ordering.

The most noticeable differences in the variable-temperature neutron PDF data occur in the region *r* = 4.1 to 4.3 Å: at 1.6 K, two distinct peaks at 4.12 and 4.31 Å are observed, while at 295 K, the intensity of these peaks drops and an additional (negative) shoulder peak at approximately 4.20 Å appears (Figure 3c). Based on the scattering factors of Mn and Mg (−3.73 and +5.375 fm^64^), these distances correspond to the distance between a Mg^2+^ cation in the *TM* layer and a next-nearest neighbor Na^+^ center (e.g., Mg1_1 and Na1_3 or Na2_1 or Mg1_2 and Na1_2 or Na2_2; Figure 3d), suggesting that the time-averaged distance between these sites is changing, likely due to Na^+^ ion motion and changes in Na site occupancies. Note that on increasing temperature, the lattice expansion would drive an increase in both distances (by approximately 0.005 Å) rather than an averaging of the two distances. A small decrease in the intensity of the peak at 2.40 Å is also seen from 1.6 to 295 K, alongside a more distinctive, better-resolved peak at 2.45 Å; both are consistent with Na^+^–Na^+^ distances (e.g., Na1_2 and Na2_3 to Na1_4; Figure 3d). The changes in intensity are consistent with Na^+^ ion hopping and occupancy changes.

Finally, it is noted that NMMO exhibits diffuse magnetic scattering in the neutron total scattering data below 40 K, but no new Bragg peaks appear, suggesting spin-glass-like behavior, as seen in other layered cathode materials (Figure S10).^63^ This highlights the absence of long-range magnetic ordering in NMMO, as explored below, using variable-temperature electron paramagnetic resonance (EPR) spectroscopy.

Overall, the PXRD, PND, and PDF results are consistent with the presence of ordering on both the *TM* and Na^+^ sublattices, as well as being consistent with a change in Na^+^ ion hopping rates/occupancies with temperature. To investigate the *TM* ordering scheme and local environments of the *TM* centers in NMMO further, variable-temperature and variable-frequency EPR spectroscopy of NMMO was carried out.

### Variable-Temperature X-Band EPR Spectroscopy

**Figure 4:**
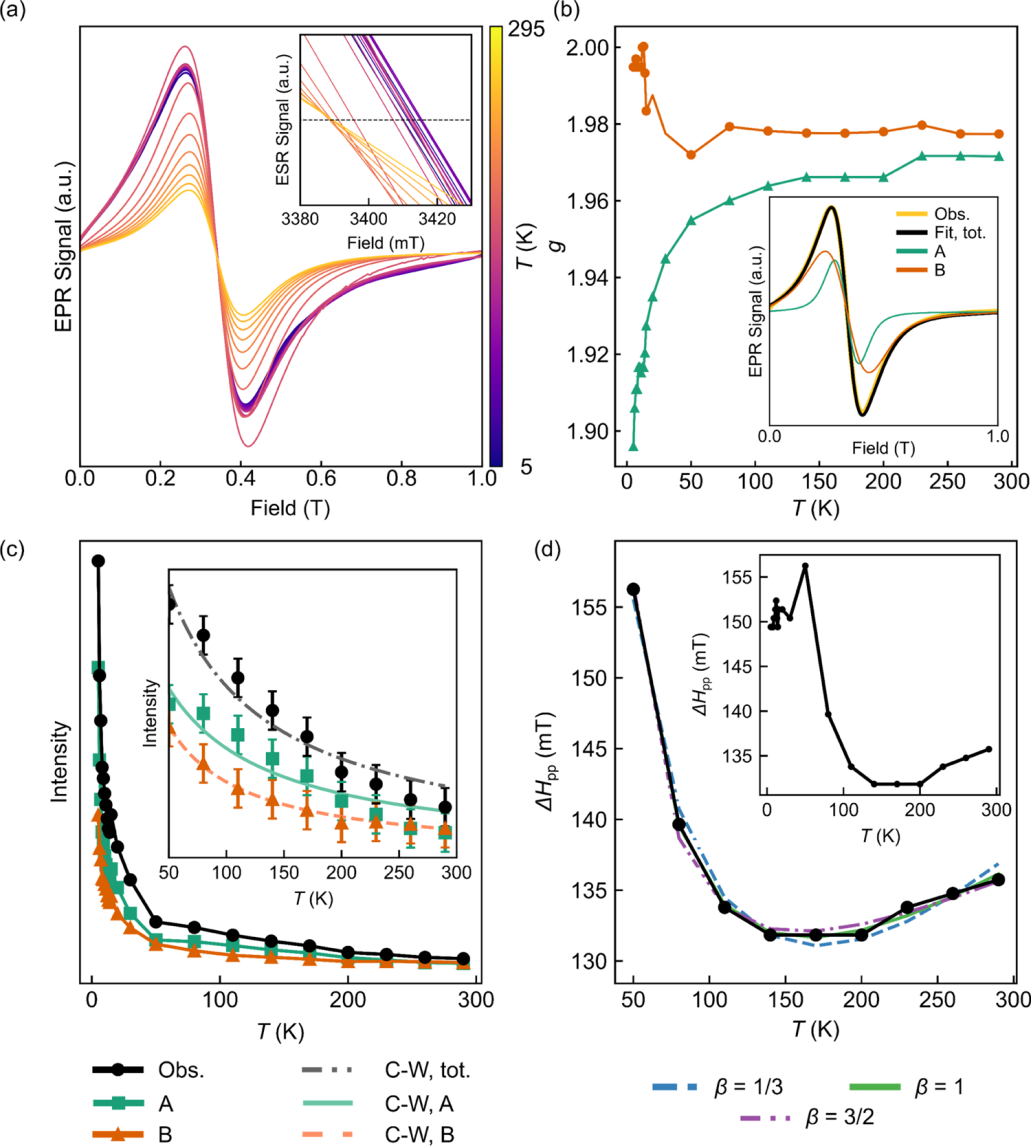
Variable-temperature X-band EPR of NMMO (a) shows the spectra acquired between 5 and 295 K, with the inset showing the subtle changes in the point at which the spectrum crosses the *x*-axis (indicating a change in *g* with temperature), while the fitted *g* values for the two resonances, A and B, are plotted in (b). The fit to the 295 K spectrum is displayed in the inset. The fitted variable-temperature intensities, (c), and line widths, (d), of the X-band EPR spectra for NMMO. Intensities are arbitrary, so no scale is shown. In (c), a line has been added between data points as a guide to the eye; the inset shows the paramagnetic region where the Curie–Weiss law was fit to the data. Error bars are shown in the inset but are omitted in the main plot for clarity. In (d), the line width is plotted against the model described in the text with three different curves, each with different critical exponents, β. The inset shows the overall variation in line width.

At X-band frequencies (*ca*. 9 GHz) at room temperature, pristine NMMO displays a broad resonance spanning the entire field sweep (0–1 T; Figure 4a). This peak-to-peak line width, Δ*H*_pp_, is dominated by the strong electron–electron dipolar interaction and also has contributions from a distribution of environments and exchange coupling (which at room temperature has a negligible effect). Only Mn^4+^ species are observed: transitions involving the electron spin microstates of Mn^3+^ cannot be seen due to the large zero-field splitting of Mn^3+^ (*S* = 2) centers.^66^

The simplest fit to the spectrum is to fit to two isotropic *g*-factors; fitting to a single isotropic *g*-factor yielded poorer results, while an anisotropic *g*-tensor or more than two resonances overfit the spectra with little improvement in the quality of the fit. The two resonances (labeled A and B, Figure 4b inset) most likely originate from Mn^4+^ ions with different numbers of Mn^3+^, Mn^4+^, and Mg^2+^ nearest neighbors. A more detailed assignment of these two features is presented later (see Discussion). We again note that, in reality, the spectrum is made up of several overlapping resonances, each corresponding to a unique local Mn^4+^ environment.

On cooling NMMO from 290 to 50 K, the X-band EPR spectrum increases in intensity, and the *g*-factors of both resonances decrease—this is more clearly seen on close inspection of the spectra and their fits (Figure 4a inset and b)—and Δ*H*_pp_ generally increases (i.e., the resonance initially narrows, then broadens; Figure 4a–d). The increase in intensity is ascribed to the increase in the static susceptibility of the sample, while the decrease in *g* and narrowing of signals on cooling can be ascribed to an exchange interaction effect.^65,66^ As temperature decreases further, spin fluctuations occur less frequently (due to less thermal randomization), the spatially anisotropic magnetic exchange interactions become poorly averaged, local magnetic environments with different anisotropic exchange interactions start to be observed, and spin clusters develop, causing a broadening of the resonances.^65^ Below 50 K, the observed signals arise from transitions between the microstates of spin clusters (these are a distinct set of spin microstates from the localized, single-center spin states seen at higher temperatures).^66^ Cooling the sample therefore enables new magnetic environments to be observed, whose resonant frequencies differ from those in the paramagnetic state and are thus difficult to detect.

The temperature variation in the maximum intensity of the overall spectrum (i.e., the combined intensity of resonances A and B together) was fit to a Curie–Weiss law (Figure 4c) between 50 and 300 K, yielding a localized mean-field Weiss constant, θ_*i*_, of −36 K, reflecting weak antiferromagnetic coupling; the measured value from bulk magnetic susceptibility measurements in the literature is also negative but is smaller (−9 K^25^). The EPR-derived Weiss constant probes only local interactions rather than the bulk average, likely accounting for the difference between the two values. Among the exchange interactions in pristine NMMO, the antiferromagnetic Mn^3+^–Mn^3+^ interaction (mediated *via e*_*g*_ orbitals and by the direct overlap between *t*_2g_ orbitals due to shortening of the JT-distorted Mn–O bonds) should be largest in magnitude but will likely have a low probability of occurrence (since, per formula unit, there are only *ca*. 0.11 equiv of Mn^3+^); the Mn^3+^–Mn^4+^ and Mn^4+^–Mn^4+^ interactions will be either weakly ferromagnetic or weakly antiferromagnetic, owing to the competing ferromagnetic superexchange (from *t*_2g_ to *t*_2g_ via intervening O 2p orbitals; see the SI, Figure S13) and antiferromagnetic direct exchange interactions; the former will have a slightly higher probability of occurrence than the Mn^3+^–Mn^3+^ interaction; the antiferromagnetic Mn^4+^–Mn^4+^ interaction will be the most abundant, accounting for the overall negative Weiss constant.

Extending the local Weiss constant method to the individual resonances, we obtain θ_A_ = −41 K and θ_B_ = −18 K. This also matches well with the assignment and expected magnitudes of the exchange interactions between Mn^3+^ and Mn^4+^. Plots of 1/intensity vs temperature revealed an approximately straight-line Curie–Weiss trend (Figure S14).

The variation in Δ*H*_pp_ as a function of temperature displays three regimes (Figure 4d): an initial, approximately linear, decrease from room temperature down to 200 K; an approximately constant line width between 200 and 140 K; finally, a rapid increase in the line width down to 50 K. Cooling below 50 K sharpens the signal, a consequence of exchange narrowing and the loss of severely broadened signals.

The temperature dependence of Δ*H*_pp_ was fit to the equation derived by Mori and Kawasaki to describe the temperature dependence of EPR line widths for low- and three-dimensional antiferromagnetic materials
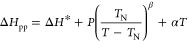
2where ΔH* is a parameter describing the exchange-narrowed line width, *P* and α are constants of proportionality, *T*_N_ is the Néel temperature (6.5 K from our previous work^20^), and β is the critical exponent. The second term in the equation describes the critical behavior between the paramagnetic regime and the (locally) ordered regime. The critical exponent, β, in eq 2 may be expressed in terms of a spin correlation length, η, its divergence ν and the divergence of the specific heat capacity of the material, ζ
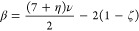
3For three-dimensional antiferromagnetic materials, β = 1/3, while two-dimensional antiferromagnets have β = 3/2.^68−73^ These quantities are in contrast to the conventional critical exponent obtained from magnetization data; the origin of this difference is complex but primarily stems from magnetization data requiring no wave-vector-dependent sums over the dipolar autocorrelation function; EPR data does require this. For a more detailed explanation, please see refs (69−71).

For NMMO, a value of β = 1/3 produces a relatively good fit (*r*^2^ = 0.6); β = 3/2 or 1 gives a poor fit (*r*^2^ = 0.1 or 0.2, respectively). However, all of these fits appear qualitatively similar, suggesting a behavior somewhere between a two- and three-dimensional antiferromagnet, as seen in other layered honeycomb-ordered materials.^71,72,74^ Overall, we may conclude that the variable-temperature X-band EPR data is consistent with Mn^4+^ centers surrounded by Mn^3+^, Mn^4+^, and Mg^2+^ neighbors in a honeycomb-ordered array.

### Room-Temperature ^23^Na NMR Spectroscopy

To investigate the consequences of cation ordering on the Na^+^ mobility in NMMO, variable-temperature ^23^Na NMR spectroscopy was carried out. We chose “intermediate” magnetic field strengths (11.7 and 16.4 T) in this work as a compromise between the strong quadrupolar and hyperfine interactions (see Figure S20 for spectra acquired at different field strengths).

**Figure 5:**
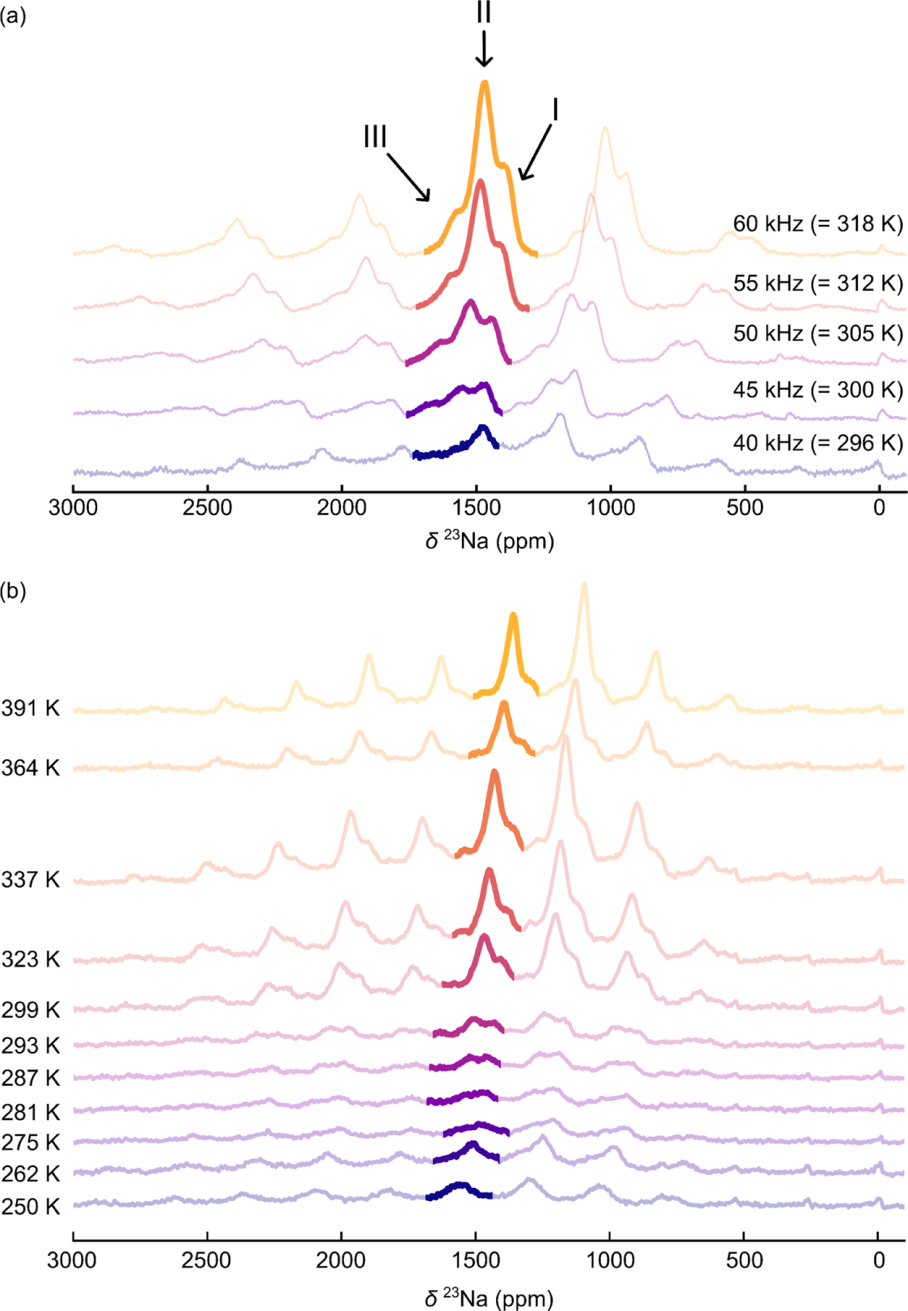
(a) “Room temperature” ^23^Na NMR spectra for P2-NMMO, acquired at different MAS rates under a field of 11.7 T; the isotropic resonances are highlighted in bold. All spectra are scaled by sample mass and number of scans. Note that “room temperature” here refers to the ambient sample temperature generated by frictional heating under MAS. The low-frequency, central, and high-frequency resonances are labeled I, II, and III, respectively, as shown in the 60 kHz spectrum. In (b), the variable-temperature 50 kHz MAS ^23^Na NMR Hahn-echo spectra at a field of 16.4 T are shown.

As shown in our previous work,^20^ the ^23^Na NMR spectrum of pristine NMMO contains four isotropic resonances: one at 0 ppm, corresponding to diamagnetic Na-containing phases (most likely surface species, such as carbonates, as seen in PXRD, Figure 2), and, at “room temperature” (approximately 318 K under 60 kHz magic angle spinning, MAS, due to frictional heating), three overlapping signals between approximately 1350 and 1550 ppm, denoted I, II, and III for the low-frequency, central, and high-frequency resonances, respectively (Figure 5a). The isotropic resonances were identified using pjMATPASS experiments (Figure S15).

The shifts of the resonances are controlled by the strong electron–nuclear hyperfine interaction between unpaired electrons on Mn and the nuclear spins of ^23^Na and, as such, are temperature-dependent due to the inherent Curie–Weiss paramagnetism of the sample (i.e., hyperfine shifts are inversely proportional to temperature).^22,26−28^ On decreasing the MAS frequency, which also decreases sample temperature, the shifts increase (Figures 5a, S18). The width of the sideband manifold is dominated by the dipolar coupling between the ^23^Na nuclei and the time-averaged magnetic moments centered on the Mn ions, which for nuclei within the layers of the α-NaMnO_2_-type structure gives rise to a characteristic asymmetric line shape.^75−78^

The breadth and intensity of the individual isotropic resonances vary with spinning: higher spinning speeds (and thus temperatures) give rise to sharper and more intense signals, while lower spinning speeds and thus temperatures yield broader, weaker signals (Figures 5a and S18).^31,79^ This, as discussed in our prior work, indicates that mobility of Na^+^ ions within the layers enters the “chemical exchange” time scale at temperatures below approximately 318 K.^31,79^ When the Na^+^ ion hopping rate between neighboring Na^+^ sites is high compared to the difference in frequencies of the sites, a sharp signal is seen, while slower hopping (compared to the site frequency difference) gives broader, less intense signals. The relative intensities of resonances I, II, and III change as a function of spinning speed/temperature. At 60 kHz, II is most intense, followed by I and then III, while at 40 and 45 kHz (296 and 300 K, respectively), I is most intense, while II and III have broadened out and decrease in intensity, again indicating that motion involving the ions that give rise to these resonances is entering the intermediate regime. Fixed-temperature, variable MAS speed experiments at 316 K and above are consistent with this (Figure S18). *T*_2_ experiments indicate that each resonance experiences a different *T*_2_ due to their unique local environments; sites experiencing shorter *T*_2_s will generally be less intense than those with longer *T*_2_s.

The most surprising observation that we need to rationalize is that there are three discrete peaks, and yet multiple Na-local environments are present in the static structure, even with a degree of cation ordering. While motion can potentially explain this, it is not immediately obvious why three peaks remain.

### Variable-Temperature ^23^Na NMR Spectroscopy

To investigate the motion further, we carried out variable-temperature ^23^Na NMR experiments at fixed spinning speeds of 50 kHz over a much wider temperature range (Figure 5b). Between 250 to 391 K, the same three observations as above are noted, i.e., an increase in the isotropic shifts with decreasing temperatures; a changeover in intensities between resonances I and II; and a broadening and overall decrease in the intensity of the resonances as temperature falls, although there are some key differences. These spectra were obtained at a field of 16.4 T, the higher field strength used to determine what (if any) effect changing the quadrupolar interaction strength would have on the spectrum, as well as to increase resolution and identify any additional peaks that may be hidden underneath the sideband manifolds. Additional variable-temperature data at 28 and 50 kHz at 11.7 T are presented in Figures S15 and S16; these results are consistent with those at 16.4 T, with no change in lineshapes from 11.7 to 16.4 T and no additional peaks were identified (Figure 5).

At 250 K, a relatively broad, low-intensity, featureless isotropic resonance is observed with a very broad spinning sideband manifold, suggesting slow Na^+^ hopping. Curie–Weiss scaling of the shifts of the resonance between 250 and 275 K to the shifts of resonances in the higher temperature spectra reveals that the low-temperature broad feature corresponds to resonance I (Figure 5b).

On heating the sample to 275 K, the peaks broaden; at 281 K, two peaks of approximately equal integrals become resolved (Figure S22). Curie–Weiss scaling indicates that this second signal corresponds to resonance II (Figure S21).

On further heating to 293 K, resonance II becomes slightly more intense than resonance I; further heating increases the intensity of II more than I (Figure 5b). At 299 K, resonance III becomes distinguishable from the baseline. Further heating increases the integral of peak II but decreases the integral of I; the integral for III remains approximately constant. I and II remain significantly sharper than III, suggesting that Na^+^ ions corresponding to III are in the intermediate regime, even at high temperatures—diagnostic of either slow hopping and/or a large difference in the exchanging sites’ shifts. The decrease in signal I’s integral (despite increasing signal height) suggests population transfer from the Na species in I to those in II. Additional attempts to reach higher temperatures than those in Figure 5b to see whether the three resonances would merge (suggesting exchange across all sites) proved unsuccessful, as the lower MAS speeds dictated by the larger rotor used to reach higher temperatures resulted in poorer resolution. Spectra acquired at lower temperatures (242 and 100 K) were also consistent with Na^+^ hopping between P(2d) and P(2b) environments; see the SI for further details (Figure S23).

### Modeling and Simulating ^23^Na NMR Spectra

To account for the effect of Na^+^ ion mobility on the observed NMR spectra, the variable-temperature spectra for NMMO were simulated using the Bloch–McConnell model described in the SI and assuming that Na^+^ ions undergo isolated hops (i.e., hops that are not influenced by other nearby Na^+^ ion hopping) between pairs of sites. Our model builds on that in previous work, where spectra were computed assuming a single hopping rate for all sites,^29^ or where shifts are computed in the high-temperature (fast-exchange) regime.^22,80^ Since only three peaks appear in the high-temperature regime, it is a reasonable first approximation to assume that three exchanging pairs will be sufficient to describe the system. For the sake of simplicity, we elected to model hops between sites with Mg^2+^ and Mn^4+^ neighbors only (i.e., no Mn^3+^ neighbors). Examining the probabilities of the different honeycomb-ordered environments reveals that the most probable site exchanges occur between these Mn^4+^/Mg^2+^-only sites (Tables S3–S5). The presence of a dynamic Jahn–Teller distortion, as well as the strong tendency of the system to order toward Na_2/3_[Mg_1/3_Mn_2/3_]O_2_ (a Mn^4+^-only system), makes modeling with Mn^3+^ challenging (see the SI, Section S8, for further details). Additional simulations that include Mn^3+^ are included in the SI to investigate what effect, if any, Mn^3+^ has on the simulations and resulting spectra [Figures S27 and S28].

**Figure 6:**
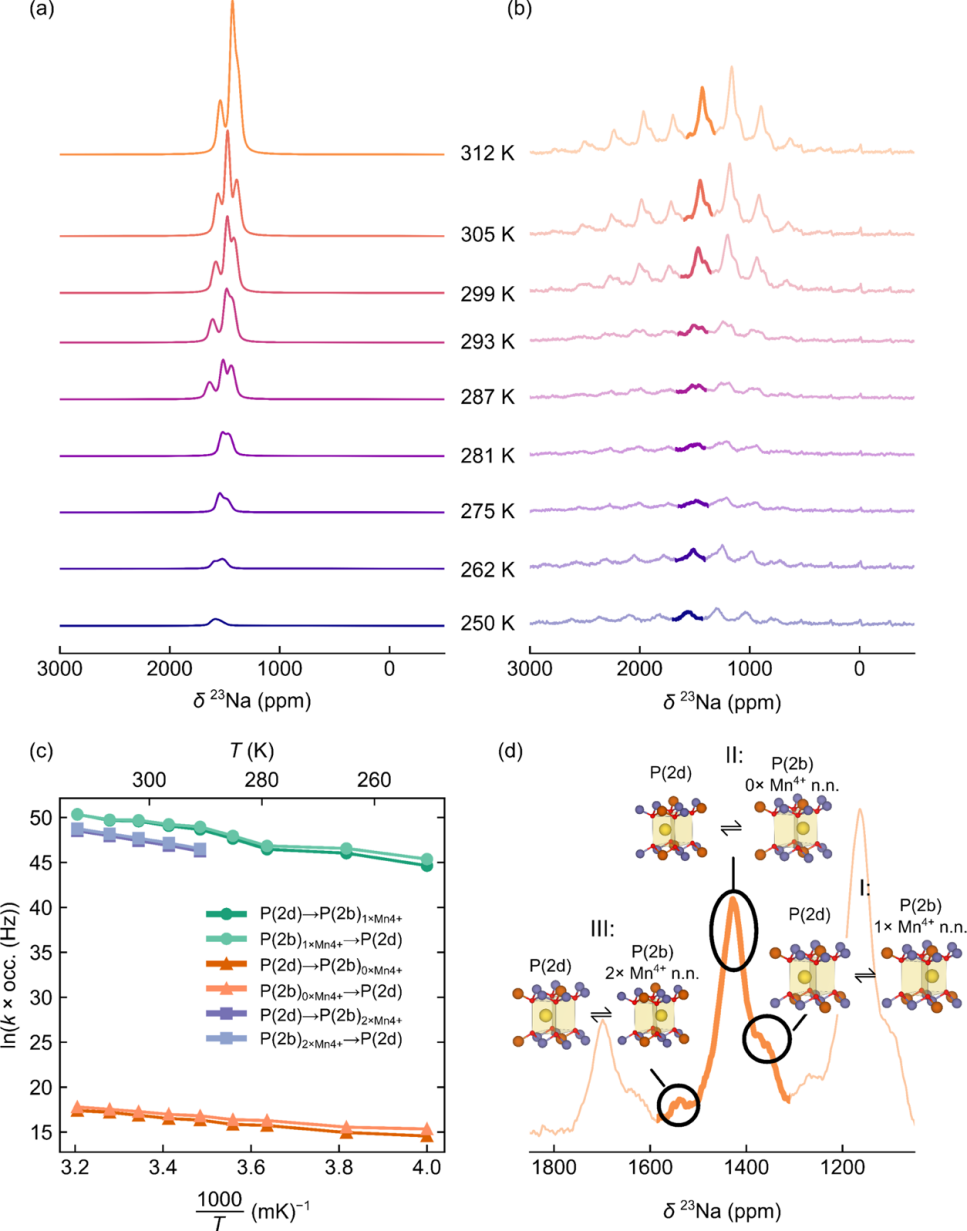
Simulations of variable-temperature ^23^Na NMR data obtained for NMMO. In (a), the simulated spectra (calculated from the Bloch–McConnell model; note that the simulations only recreate the isotropic peaks) are shown, with the experimental spectra in (b). The hopping rates for “forward” (P(2d) to P(2b)) and “reverse” (P(2b) to P(2d)) hops are shown in (c), and the final assignment of the spectra is shown in (d).

The simulation results and extracted hopping frequencies are listed in Figure 6. The simulations were iterated by varying the hopping frequencies of each site while keeping the hopping attempt rate constant and the shifts of static resonances fixed to those obtained from calculations (scaled to the experimental sample temperature using the Curie–Weiss law).

The spectra were modeled using all possible combinations of Na^+^ sites in the honeycomb Na_2/3_[Mg_1/3_Mn_2/3_]O_2_ model. Of these, only one model reproduced the observed data; resonance I corresponded to Na^+^ ions hopping between a P(2d) site and a P(2b) site with one Mg^2+^ nearest neighbor and one Mn^4+^ nearest neighbor; resonance II corresponded to Na^+^ ions hopping between a P(2d) site and a P(2b) site with two Mg^2+^ nearest neighbors; and III corresponded to Na^+^ ions hopping between a P(2d) site and a P(2b) site with two Mn^4+^ nearest neighbors (Figure 6d); see Discussion for the connection between each of the peaks and the environments in the supercell obtained from XRD. Simulations using the same parameters also provided a good fit to the lower field (11.7 T) spectra acquired at 28 and 50 kHz, further validating the model (Figures S19 and S20). Given the good fit to the data, alternative models with more than three pairs of exchanging ions do not appear justified, although much slower exchange between ions in different pairs will likely be occurring.

The hopping rates extracted from the simulations reveal an approximately linear trend in the log of the hopping rates, *k*, as a function of the inverse of sample temperature, *T*, between 250 and 312 K (Figure 6c), as expected for a constant activation energy barrier to hopping with respect to temperature. An Arrhenius fit of this line yielded energy barriers between 260 and 380 meV (Figure S26). Deviations from an ideal straight line likely arise from small changes to the barrier due to changes in bond angles and lengths (causing small changes to the bond pathways). It is also anticipated that the effect of the dipolar and quadrupolar interactions (not accounted for here) will influence the line shape; accounting for these contributions and the effect of correlated motion is beyond the scope of this work but presents an interesting avenue of future research.

### *Ab Initio* Calculations of the Na^+^ Ion Hopping Barriers

To compare the extracted hopping rates from ^23^Na NMR simulations, the energy barriers to Na^+^ ion hopping in NMMO were determined using a series of climbing image nudged elastic band (CI-NEB) calculations on a set of model systems, namely, P2–Na_1/2_MnO_2_, P2–Na_1/2_Mg_1/6_Mn_5/6_O_2_, and honeycomb-ordered P2–Na_2/3_Mg_1/3_Mn_2/3_O_2_; see Section S9 of the SI for further details and results. The gradual increase in Mg content was applied to see what effect Mg has on the energy barriers associated with hopping between the P(2d) and P(2b) sites. In each case, a (2 × 2 × 1) supercell was constructed; the P2–Na_1/2_MnO_2_ and P2–Na_1/2_Mg_1/6_Mn_5/6_O_2_ systems achieved good *k*-mesh and plane-wave energy cutoff convergence, but the P2–Na_2/3_Mg_1/3_Mn_2/3_O_2_ system could not be converged, regardless of cell size or distribution of Mg and Na ions. For all Na^+^ hops investigated, the hopping Na^+^ ion begins in a P(2d) site and ends up in a P(2b) site with a different local environment (i.e., a different number of Mg^2+^, Mn^3+^, and Mn^4+^ nearest neighbors); in all cases, the P(2b) site was higher in energy than the P(2d), as expected on Coulombic repulsion grounds.

In the case of Na_0.5_MnO_2_, the energy barrier to hopping was approximately 180 meV, while that for Na_1/2_Mg_1/6_Mn_5/6_O_2_ was approximately 270 meV (Figure S32). These barriers are consistent with those extracted from the Arrhenius fits from the NMR simulations. The increased hopping barrier to a site with Mg^2+^ nearest neighbors is perhaps unsurprising: to keep a net charge of +2 from the nearest neighbor cations around O^2–^ (i.e., to remain locally electroneutral), the oxidation state of Mn must increase from +3 to +4 when Mg^2+^ is introduced. The increase in the Mn oxidation state increases Coulomb repulsion with nearby Na^+^, raising the barrier to Na^+^ hopping.

Intriguingly, CI-NEB runs with Mg-doped into the *TM*O_2_ layer resulted in more cooperative Na^+^ ion motion, while in all systems, when one Na^+^ ion was set to hop, others also changed the position, and when Mg^2+^ was introduced, more Na^+^ ions nearby the hopping Na^+^ center moved and/or the distance moved by nearby Na^+^ ions increased. It is anticipated that this cooperative motion results in a relatively shallow potential energy surface, meaning that converging in a transition state becomes challenging. The cooperative motion can also be ascribed to a tendency toward electroneutrality around O^2–^: if the O^2–^ center loses a Na^+^ nearest neighbor, the nearby Na^+^ ions will hop toward the vacancy to minimize the change in local charge (removing one Na^+^ from the O^2–^ first coordination shell decreases the positive charge delivered to O^2–^ by +1/6, as each Na^+^ is bound to six O^2–^). As a result, a single Na^+^ ion hopping will encourage nearby ions to hop in such a way that it preserves electroneutrality and, therefore, the local ordering of Na^+^ ions.

**Figure 7:**
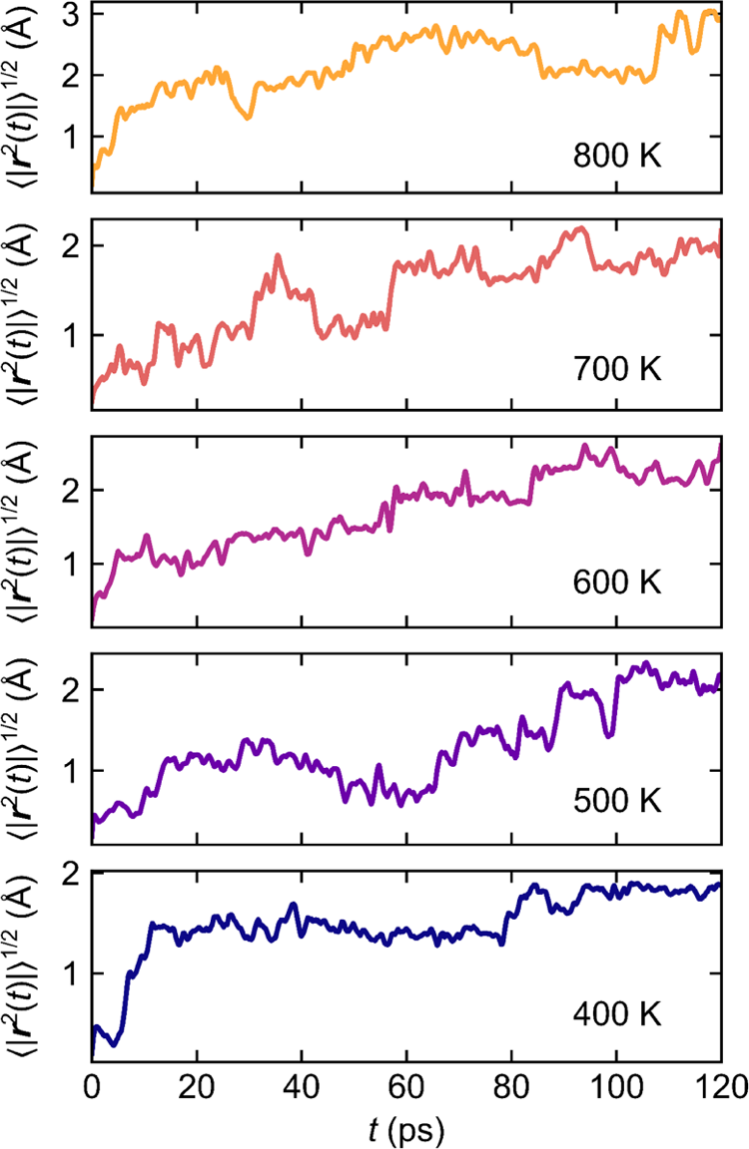
*Ab initio* molecular dynamics results for Na^+^ ions diffusing in P2–Na_2/3_[Mg_1/3_Mn_2/3_]O_2_ (*a* ≈ 10.2 Å, *b* ≈ 10.2 Å, *c* ≈ 11.4 Å), showing the root-mean-square displacement of Na^+^ ions, ⟨*r*^2^(*t*)⟩^1/2^, between 400 and 800 K.

This strongly cooperative motion is also reflected in *ab initio* molecular dynamics (AIMD) simulations of (2 × 2 × 1) supercells (*a* = *b* ∼ 10 Å, *c* ∼ 11 Å) of Na_2/3_Mg_1/3_Mn_2/3_O_2_ (Figure 7): hops from one Na^+^ to the next are not independent, but instead are cooperative, with one hopping Na^+^ causing its neighbors to also (begin to) hop. Instead of a linear trend in the root-mean-square (rms) displacement, ⟨Δ*r*(*t*)^2^⟩, with time, an initial sharp rise in ⟨Δ*r*(*t*)^2^⟩ is seen, followed by an oscillatory rms displacement about an approximately constant value (*ca*. 40 Å^2^), suggesting that, after an initial perturbation, Na^+^ ions do not necessarily displace in a way to explore a large region of space but instead remain constrained to a small region of space. It is acknowledged, however, that this picture is limited as the simulation time is relatively short, and only a few hundred Na^+^ hops are observed.

This variation in ⟨Δ*r*(*t*)^2^⟩ with time has been previously seen in similar cooperative motion systems, for example, in polymer-in-solution systems^81,82^ and other ionic systems,^83^ and has also been explored in other NIB cathodes, where Na^+^ ion motion was seen to be significantly faster at or near antiphase boundaries between Na^+^/vacancy ordered regions. Our results indicate that Na^+^ ion motion in NMMO is cooperative and likely driven by local electroneutrality.

## Discussion

The tendency of pristine NMMO to adopt an ordered structure in which Mg and Mn are approximately honeycomb ordered and the Na sublattice is also ordered can be understood by a tendency for local electroneutrality, as seen in other cathode systems.^84^ Ideally, each O^2–^ center would be coordinated by three species in the *TM* layer (Mg^2+^, Mn^3+^, or Mn^4+^) and zero, one, two, or three Na^+^ ions in the adjacent layer. From the perspective of an O anion (charge −2), there are always three *TM*s in the first coordination sphere (ignoring vacancy defects) and either zero, one, two, or three Na^+^ neighbors. Since each of the *TM* and Na^+^ centers shares their charge with six O centers (assuming no O^2–^ vacancies), each O receives one-sixth of a metal center’s charge.

Local electroneutrality can therefore be achieved only in six ways (assuming a random distribution of all cationic species; Table 2). Based on the 2/3 occupancy of the Na^+^ sublattice, the probability of an O^2–^ center having no Na^+^ nearest neighbors is approximately 4%, while the probability of having one Na^+^ nearest neighbor is 22%, two Na^+^ nearest neighbors is 44%, and three Na^+^ nearest neighbors is 30%. When combined with the probability of the occupancies of the *TM* sites, we find the most probable environments are environments (1), (3), and (5); O(1) has no Na^+^ neighbors, so it is very unlikely given the high Na^+^ content in NMMO, while O(3) and O(5) correspond to locally honeycomb-ordered environments and are closest in composition to the bulk stoichiometry. These two constitute over half of the electroneutral environments.

**Table 2:** Locally Electroneutral Sites Around the O^2–^ in Pristine NMMO, with Probabilities of Each Environment Based on the Occupancies of the Na and *TM* Sublattices

	no. of Na^+^ n.n.	no. of Mg^2+^ n.n.	no. of Mn^3+^ n.n.	no. of Mn^4+^ n.n.	probability (%)
(1)	0	0	0	3	28.4
(2)	1	0	1	2	15.4
(3)	2	1	0	2	39.1
(4)	2	0	2	1	2.8
(5)	3	1	1	1	14.1
(6)	3	0	3	0	0.2

The presence of (at least local) honeycomb order on the *TM* sublattice is also evident from X-band EPR, where two resonances, A and B, were observed. In previous work, Stoyanova et al. observed larger *g*-shifts (i.e., greater deviation of the *g*-factor from the free-electron *g*-value, *g*_e_) for centers in Na_*y*_[Co_1–2*x*_Ni_*x*_Mn_*x*_]O_2_ (*x* = 1/2, 1/3; *y* = 1/2, 1/3) with strong local exchange couplings, while species with weaker exchange couplings showed a *g*-value closer to *g*_e_.^78^ This may be rationalized by considering that strong exchange coupling augments the applied field significantly, resulting in a large apparent *g*-factor.^79,80^ In the case of Mn^4+^, the less-than-half-filled shell results in a *g* lower than *g*_e_.^22^ If these Mn^4+^ experience strong superexchange, the *g*-values will be even further from *g*_e_. Since Mn^3+^ typically has a stronger exchange than Mn^4+^ (as the Mn^3+^ exchange interactions are mediated by *e*_g_ orbitals), the more Mn^3+^ centers near Mn^4+^, the lower the *g*-factor is expected to be.^78^ It is also expected that more Mg^2+^ centers around a Mn^4+^ will move *g* closer to *g*_e_, as Mg has a smaller spin–orbit coupling constant than Mn and therefore a smaller influence on the *g*-factor of the unpaired electron.

**Figure 8:**
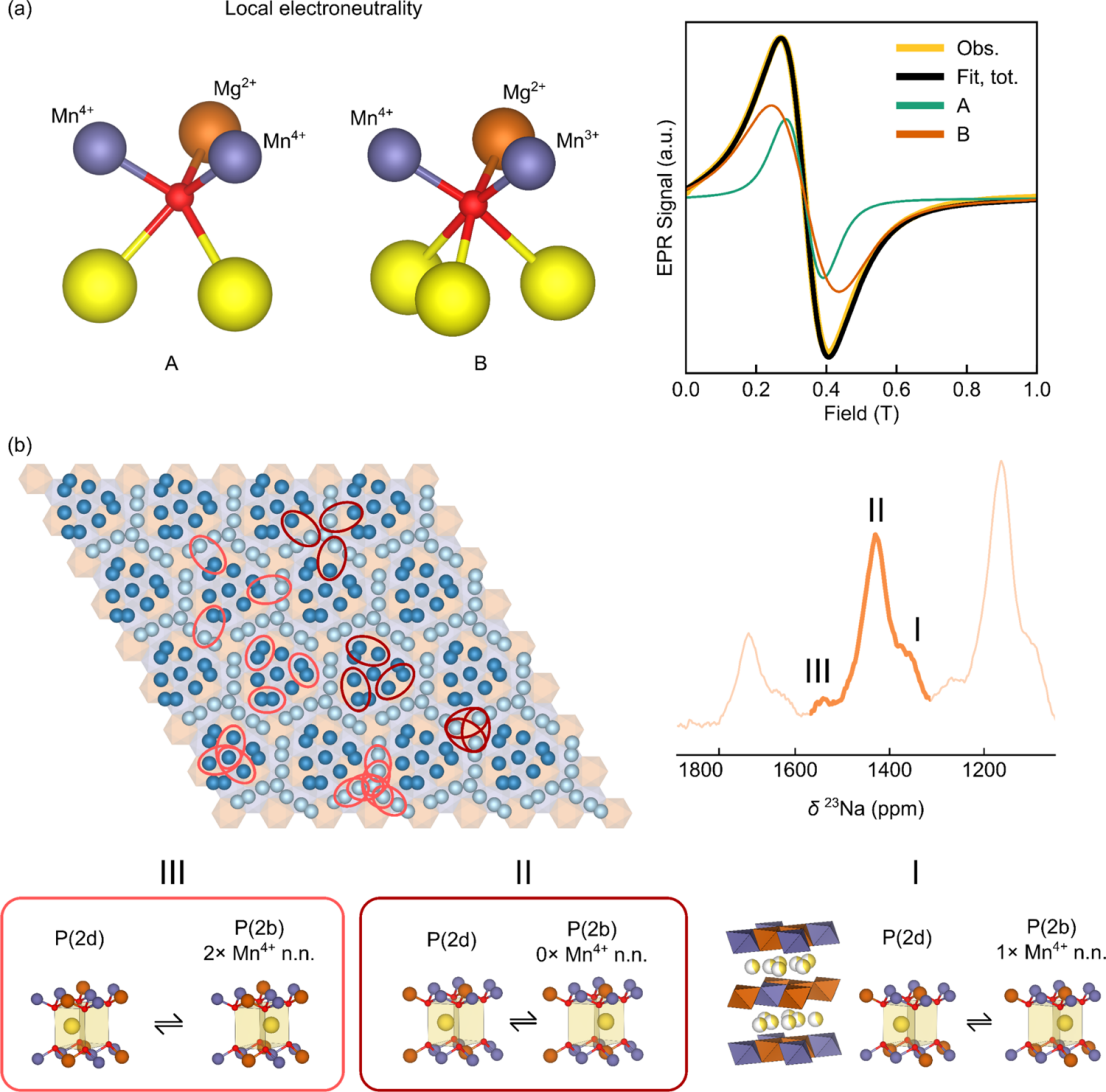
Identifying the superstructure with (a) EPR and (b) NMR observations. In (a), the two most likely local electroneutral environments around O are depicted and assigned to the two resonance classes seen in EPR, A and B. In (b), a view down the *c*-axis of the Na^+^/vacancy ordering obtained from PXRD refinements is shown, with loops drawn around pairs of Na^+^ ions which exchange between the local environments shown below, labeled as I, II, and III, corresponding to the three resonances seen in ^23^Na NMR, shown to the right.

Based on these observations and assumptions, resonance A (with a *g*-factor further from *g*_e_) can be tentatively assigned to Mn^4+^ with at least one Mn^3+^ nearest neighbor and resonance B to Mn^4+^ with only Mn^4+^ and Mg^2+^ nearest neighbors. The presence of JT-distorted Mn^3+^ near Mn^4+^ will likely also increase the spin–orbit coupling contribution to the *g*-factor.^22^ This is also consistent with observations that Mn^4+^ species in a Mn^4+^-only lattice have *g*-factors of approximately 1.98, as observed (Figure 4b).^67,81,82^ On the basis of empirical observations by Stoyanova et al.,^85−89^ in which species with stronger exchange interactions have larger *g*-shifts, A may be assigned to Mn^4+^ with one Mn^3+^ neighbor, two Mn^4+^ neighbors, and three Mg^2+^ nearest neighbors (i.e., electroneutral environment (5)) and B may be assigned to Mn^4+^ with three Mn^4+^ neighbors and three Mg^2+^ neighbors (electroneutral environment (3); Figure 8a). Additional high-frequency EPR results also indicated the formation of spin clusters below 50 K, consistent with the temperature at which the field-cooled and zero-field cooled susceptibility bifurcate^25^ and the point below which diffuse magnetic scattering in neutron diffraction is observed (Figure S10). These spin clusters likely develop through competing exchange interactions between domains of the lattice with different *TM* and Na^+^/vacancy orderings.

The effect of ordering on the *TM* sublattice has consequences for the Na^+^ sublattice: Na^+^ ions adopt a “long-zigzag” (LZZ)-like configuration, as seen in other cathode materials with a Na^+^ content *x* = 0.67.^9,10,64,90−93^ Here, Na^+^ ions can be grouped into those Na^+^ in the zigzag regions of the LZZ structure, Na_*a*_, or the island regions, Na_*b*_. Based solely on the vacancy concentration, the former is anticipated to be more mobile than the latter. This is also borne out in the observed and simulated variable-temperature ^23^Na NMR spectra (Figure 6): resonances II and III may be ascribed to Na^+^ ions in layers with LZZ-like ordering, where Na^+^ ion hopping occurs between P(2d) sites and P(2b) sites with two Mg^2+^ nearest neighbors (and no Mn^4+^ nearest neighbors) or between P(2d) and P(2b) with two Mn^4+^ nearest neighbors (and no Mg^2+^ nearest neighbors, Figure 8b), respectively. We see only three peaks because the exchange is a local rather than a long-range process (on the time scale probed by the NMR experiments) in which Na^+^ ions explore only two local environments (the initial and final states), such that local electroneutrality before and after the hop is preserved. The tendency of NMMO to order limits the total number of local environments, resulting in a spectrum with many fewer lines than expected.

Resonance II is assigned to Na^+^ ions hopping between P(2d) and P(2b) with two Mg^2+^ nearest neighbors. Of all of these local exchanges, one-third arise from Na_*a*_ ↔ Na_*a*_ hopping, one-third from Na_*b*_ ↔ Na_*b*_ hopping, and one-third from Na_*a*_ ↔ Na_*b*_ hopping. Here, the difference in the resonant frequency of the exchanging Na^+^ sites is small; since resonance II is sharp and high in intensity at most temperatures but rapidly decreases in intensity at lower temperatures, we anticipate the barriers to hopping to be small, such that these ions are (on the NMR time scale) in the fast (high *T*) or intermediate (lower *T*) regime of motion. The low barriers likely stem from the low Coulomb repulsion experienced in the P(2b) site coordinated by two Mg^2+^ neighbors, in contrast to the higher repulsion expected when either one or two Mn centers coordinate the P(2b) site. The relative energies of these pathways are also consistent with the bond valence energy landscapes obtained from the PXRD Rietveld-derived structures (Figure S12). We note, however, the difference in the barriers to hopping to a P(2b) site with only Mg^2+^ neighbors and to hopping to a P(2b) with Mn neighbors (according to bond valence energy landscapes) is approximately 100 meV, so not significantly larger.

When Na^+^ ions hop between P(2d) sites and P(2b) sites with two Mn^4+^ nearest neighbors, the difference in resonant frequency is large: approximately 1840 ppm at 318 K. We therefore suggest that, due to the large difference in site frequencies, these Na^+^ centers only enter the fast-hopping regime at the highest temperatures. On this basis, we assign these local hops to ^23^Na resonance III, consistent with simulations. These local environments are present in both Na_*a*_ and Na_*b*_ sites seen in PXRD (Figure 2f); approximately 40% of these local exchanges are from Na_*a*_ ↔ Na_*a*_ hopping, 40% is from Na_*b*_ ↔ Na_*b*_ exchanges, and 20% from Na_*a*_ ↔ Na_*b*_ exchange. Again, the barrier to this hopping is not expected to be significantly larger than that for the exchange in resonance II, as these hops were those seen most clearly in variable-temperature PXRD and PDF (Figure 3d).

Resonance I is assigned to Na^+^ ions in the parent-like layers, where the adjacent *TM*O_2_ layers have a honeycomb arrangement of Mg and Mn, but the relative lateral positions of Mg and Mn from one layer to the next are random. Such local environments may also be achieved in antiphase boundaries between different domains of the crystallites. The loss of order in these regions results in a loss of LZZ-like ordering of these layers, with Na^+^ ions instead randomly occupying sites in these parent material layers. It is expected that the energy barrier for hops between these sites lies between the barriers for the Na^+^ ions corresponding to resonances II and III, again based on the local vacancy concentration. Since the difference in the ^23^Na NMR frequencies of the island sites (either P(2d) and P(2b) with two Mg^2+^ nearest neighbors or P(2d) and P(2b) with two Mn^4+^ nearest neighbors) is, on average, lower than the difference for the honeycomb border sites (Figures 7, S29) but the rate of hopping is likely slower, it is expected that the island sites will behave in a fashion similar to the honeycomb border Na^+^ ions, i.e., resonances from these Na^+^ ions will only sharpen at high temperatures, where Na^+^ is in the fast-hopping regime; at lower temperatures, the resonance will remain broad and low in intensity, as observed.

Finally, it is noted that Na^+^ ion motion in NMMO (both in the parent phase and superstructure) can readily occur because all the Na sites are partially occupied; motion is, however, expected to be highly correlated based on CI-NEB and AIMD results. This cooperativity is likely a direct consequence of retaining local electroneutrality, with one Na^+^ hop resulting in different Na^+^–Na^+^ and Na^+^–*TM* Coulombic interactions; it explains, at least in part, the difficulty in obtaining a good Rietveld fit of the long-range structure to the observed PXRD and neutron diffraction (Figure 2b) while faithfully capturing the average local structure in both NMR and PDF. In other words, the cooperative Na^+^ ion motion results in several local ordering schemes, each of which could be described by a superposition of several symmetry modes describing Na^+^ displacement.

The superstructure adopted by layered NIB cathodes such as NMMO has important consequences for the electrochemical performance of the cathode. In general, the presence of ordering on the Na sublattice makes Na^+^ ion diffusion sluggish compared to disordered materials, owing to the increased (enthalpically driven) energy barrier to Na^+^ ion hopping;^7^ this will likely result in a large activation barrier to Na^+^ extraction at the early stages of desodiation to overcome the energy barrier to disrupting the Na/vacancy ordering.^6,8−10^ Indeed, the Na_0.67_Mg_*y*_Mn_1–*y*_O_2_ family (of which NMMO is a member) adopts multiple polymorphs depending on the stoichiometry of the material and synthetic conditions, with each member of the family having different electrochemical performances depending on the Mg content.^94,95^ The best performance was observed for Na_0.67_Mg_0.10_Mn_0.90_O_2_, where the random distribution of Mg in the *TM* layer disrupted Na^+^ ion ordering, leading to large capacities even at high cycling rates. In NMMO, the superstructure is expected to enable fast Na^+^ ion motion for the Na^+^ in the hexagonal borders but slower motion in the island sites, as seen from NMR simulations. The ordering on the *TM* site is anticipated to have implications for the electronic structure of NMMO: it has been proposed by several authors that the superstructure in layered NIB (and lithium-ion battery) cathodes can enable more reversible oxygen redox reactions by developing more delocalized states or by facilitating *TM* migration and pairing of oxidized O centers to form O_2_.^15^ Therefore, the superstructure present in the pristine material may be the cause of the high capacities at relatively fast cycling rates seen in NMMO.

## Conclusions

In conclusion, an ordering scheme accounting for the additional superstructure reflections in Na_0.67_[Mg_0.28_Mn_0.72_]O_2_ has been proposed using synchrotron X-ray and neutron diffraction, pair distribution function analysis, variable-temperature ^23^Na NMR, and variable-temperature and -frequency EPR. This ordering arises from a tendency for local electroneutrality.

By carefully modeling the PXRD, an average bulk structure was generated using distortion modes on the Na and *TM* sublattices to describe ordering on both the *TM* and Na sublattices. It is acknowledged that the true superstructure may be a combination of two different *k*-vectors (supercells), one for each sublattice, or that it contains more subtle distortions, with more modes active within the supercell. We note, however, that the peaks that are not well fit are small relative to the main peaks in the pattern.

At a local level, the superstructure presented in this work is consistent with neutron PDF, variable-temperature, variable-frequency EPR, and variable-temperature NMR. Analysis of the EPR data revealed at least two Mn^4+^ environments, which differ in the number of Mn^3+^ and Mn^4+^ nearest neighbors, rationalized by considering the magnetic exchange interactions for each unique resonance. A thorough analysis of the observed ^23^Na NMR spectra using Bloch–McConnell simulations enabled the mobilities of three distinct honeycomb Na^+^ environments to be identified, rationalized, and explained within the superstructure ordering model. It was determined that those Na^+^ closest to Mg^2+^ centers hop fastest, while those closer to Mn^4+^ centers hop at a much slower rate, presumably on account of the lower repulsion from Mg giving smaller activation barriers.

Our analysis shows that the relatively simple ^23^Na NMR spectrum at room temperature stems from the presence of short-range, rather than long-range, hopping of Na^+^ ions between sites. In other words, Na^+^ ions explore only a relatively small region of space. Furthermore, hops encourage nearby Na^+^ ions to move to preserve local electroneutrality, resulting in little change in the short- and long-range structures before and after hopping.

The methodology presented in this work—studying local and bulk structures using noninvasive scattering and magnetic resonance techniques in combination with *ab initio* calculations and simulations—is critical to understanding the local structures adopted by cathode materials and the consequence of superstructures on their electrochemical performance.

## Supporting Information Available

The Supporting Information contains a detailed experimental, additional variable-temperature ^23^Na NMR, NMR simulations, additional Rietveld refinements, details on calculations and assignments, and a discussion of the theory behind the Bloch–McConnell equations (PDF)
