# Supplementary material for: Superstructure and Correlated Na+ Hopping in a Layered Mg-Substituted Sodium Manganate Battery Cathode are Driven by Local Electroneutrality
Source: Chem Mater. 2023 Dec 7;35(24):10564–83. doi: 10.1021/acs.chemmater.3c02180 (PMC10753809; doi:10.1021/acs.chemmater.3c02180)
Supplement: Supplementary file 1 — cm3c02180_si_001.pdf [file cm3c02180_si_001.pdf]

Supporting Information

Superstructure and Correlated Na^+ Hopping in a Layered Mg-substituted Sodium Manganate Battery Cathode are driven by Local Electroneutrality

Euan N. Bassey,[1,a] Ieuan D. Seymour,[2,b] Joshua D. Bocarsly,[1,c] David A. Keen,[3] Guido Pintacuda,[4] Clare P. Grey[1,*]

[1]Yusuf Hamied Department of Chemistry, University of Cambridge, Lensfield Road, Cambridge, CB2 1EW, United Kingdom

[2]Department of Materials, Imperial College London, South Kensington Campus, London, SW7 2AZ, United Kingdom

[3]ISIS Facility, STFC Rutherford Appleton Laboratory, Harwell Oxford Campus, Didcot, OX11 0QX, United Kingdom

[4]Centre de RMN à Très Hauts Champs, UMR 5082 (CNRS/Université Claude Bernard Lyon 1/Ecole Normale Supérieure de Lyon), University of Lyon, 69100 Villeurbanne, France

[a]Present address: Materials Research Laboratory, University of California, Santa Barbara, Santa Barbara, CA 93106-5121, United States of America

[b]Advanced Centre for Energy and Sustainability (ACES), Department of Chemistry, School of Natural and Computing Sciences, University of Aberdeen, King's College, AB24 3UE Aberdeen, Scotland, UK

[c]Present address: University of Houston, Department of Chemistry, Lamar Fleming Jr. Building, 3585 Cullen Blvd, Room 112, Houston, Texas 77204-5003, United States of America

* To whom correspondence should be addressed:

E-mail: cpg27@cam.ac.uk

Contents

S1. Experimental 5

S2. Modelling the effect of Na^+ hopping on the NMR spectra 10

S3. Variable-temperature NMR temperature calibration 13

S4. Additional SXRD and Neutron Refinements 15

S5. Bond Valence Sum Analysis 23

S6. Magnetic Exchange interactions and EPR Weiss constant fits 24

S7. Additional variable-temperature ^{23}Na NMR results 26

S8. Bond pathways in NMMO, assigning the NMR spectrum and differences between models used 41

S9. Additional *ab initio* calculations of Na^+ hopping pathways 50

List of Figures

Figure S1: demonstration of the exchange simulation script. The distinct resonant frequencies were chosen as 500 ppm (henceforth site A) and 1500 ppm (henceforth site B), with forward and reverse energy barriers of hopping of 310 meV (chosen to be equal for the sake of simplicity). A hopping attempt frequency of 10^{12} Hz was chosen to represent that in a typical solid. The temperature ranges from 200 K (bottom) to 320 K (top). 12

Figure S2: *Ex situ* variable-temperature NMR temperature calibration curve, obtained from the ^{207}Pb shift of $Pb(NO_3)_2$ 13

Figure S3: *In situ* variable-temperature NMR temperature calibration curves, obtained from the ^{79}Br T_1 time or shift of KBr. **(a)** shows the curve obtained using the T_1 time as a metric for the temperature, using a field of 16.4 T and an MAS rate of 50 kHz; **(b)**, **(c)** and **(d)** show the curves for the ^{79}Br shift at 11.7 T under 30 kHz, 40 kHz and 50 kHz MAS, respectively. Black lines show the fitted straight lines to each set of data points (green). 14

Figure S4: Rietveld refinements of SXRD data for NMMO at room temperature, focussing on the superstructure peaks. **(a)** and **(b)** show the refinement in the regions Q = 0.70 – 0.95 Å^{-1} and Q = 1.75 – 2.25 Å^{-1}, respectively. In **(c)** and **(d)**, the structures of the superstructure from the main text and the refined superstructure from the refinement in **(a)** are shown, respectively. 15

Figure S5: Rietveld refinements of both the parent and (2 × 2 × 1) superstructure to **(a)** synchrotron X-ray diffraction and **(b)** to **(d)** powder neutron diffraction patterns banks 3 to 5 respectively; $R_{w.p.}$ 3.93 %. Insets in **(a)** show some of the superstructure reflections in the regions Q = 0.85 – 0.95 Å^{-1} and Q = 1.75 – 2.25 Å^{-1}. 16

Figure S6: Rietveld refinements of the parent structure against **(a)** the room temperature synchrotron X-ray diffraction data, and **(b)**, **(c)** and **(d)** the room temperature neutron diffraction data for banks 3 – 5; $R_{w.p.}$ = 14.7% 17

Figure S7: Neutron pair distribution function data for NMMO at room temperature, with fit to the parent structure only. 18

Figure S8: Rietveld refinements of the parent and (2×2×1) superstructure with Mn and Mg occupancies swapped relative to the superstructure presented in the main text against **(a)** the room temperature synchrotron X-ray diffraction data, and **(b)**, **(c)** and **(d)** the room temperature neutron diffraction data for banks 3 – 5; $R_{w.p.}$ = 8.9% 19

Figure S9: Neutron pair distribution function data for NMMO at room temperature, with Rietveld fit to the superstructure with Mg and Mn swapped. 20

Figure S10: Difference in the total neutron scattering, ΔI(Q), of NMMO at 1.6 K and 40 K, relative to the scattering at 130 K, revealing diffuse magnetic scattering near Q = 0.5 Å^{-1} (arrowed). 21

Figure S11: **(a)** shows an expansion of the variable-temperature SXRD patterns between Q = 2.59 Å^{-1} and Q = 2.72 Å^{-1}, corresponding to the (204) reflection, as indicated. In **(b)**, the simulated effect of the GM_1^+ and M_2^+ displacive modes on the diffraction pattern is highlighted: when the arrowed Na^+ ions hop away from the plane from a P(2b) site with two Mn^{4+} nearest neighbours to a P(2d) site, the (204) reflection decreases in intensity (right). Note that the shading of the (204) plane indicates Na^+ centres which are above (lighter colour) and below (darker colour) the (204) plane. 22

Figure S12: Computed bond valence sum map for NMMO. For clarity, only the Na layer is shown. 23

Figure S13: Schematics showing the exchange interactions between Mn^{3+} and Mn^{4+} centres in NMMO, labelled ferromagnetic (F) and antiferromagnetic (AF). 24

Figure S14: Observed and fitted (resonances A and B) inverses of EPR intensities as a function of temperature. Inset shows the fit to the paramagnetic regime, above 80 K. 25

Figure S15: Hahn-echo and isotropic projection of pjMATPASS spectra acquired at 60 kHz and 40 kHz MAS speed, under an applied field of 11.7 T 26

Figure S16: Variable-temperature ^{23}Na NMR spectra for NMMO recorded at 11.7 T under 50 kHz MAS rate. Isotropic resonances are highlighted in bold. 27

Figure S17: Variable-temperature ^{23}Na NMR spectra for NMMO recorded at 11.7 T under 28 kHz MAS rate. Isotropic resonances are highlighted in bold. 28

Figure S18: Fixed-sample-temperature, variable MAS frequency ^{23}Na NMR spectra acquired for P2-NMMO at 11.7 T, with isotropic resonances highlighted in bold. Spectra are scaled according to number of scans and mass of sample. 28

Figure S19: T_2-filtered Hahn-echo experiments; the echo delay (time between the 90° and 180° pulses) was varied and a spectrum recorded. Inset shows a zoom-in on the isotropic region of the spectrum. Spectra were recorded under 50 kHz MAS (sample temperature 305 K) and an applied field of 11.7 T. 29

Figure S20: Hahn-echo spectra of NMMO acquired at a spinning speed of 60 kHz and under magnetic fields of 4.7 T and 11.7 T. 30

Figure S21: Observed ^{23}Na NMR shifts as a function of temperature for the data acquired at 16.4 T, with Curie-Weiss law fits to the inverse of the shift as a function of temperature. Shaded region represents the estimated error in the observed isotropic shift. 31

Figure S22: **(a)** example fit of the Hahn-echo spectrum of NMMO at 337 K (50 kHz, 16.4 T). In **(b)**, the temperature variation in T_2-weighted integral (isotropic and sidebands) of each component in the fit. Fill along the lines indicates the approximate error in the integral. 32

Figure S23: Low-temperature ^{23}Na NMR spectra for pristine NMMO and comparison to calculated shifts, which were obtained from bond pathways assuming a random distribution of Mg, Mn^{3+} and Mn^{4+} centres or a honeycomb distribution of Mg and Mn^{4+} cations. In **(a)**, the 242 K data at 12.5 kHz MAS and 11.7 T is shown, whilst **(b)** shows the 100 K data at 30 kHz MAS and 9.4 T; both spectra were obtained *via* variable offset cumulative spectroscopy. The asterisk denotes the resonance from Cu in the coil. Note that the simulated spectra contain isotropic resonances only. Arrows above the experimental spectra indicate the centre-of-mass shift for P(2d) and P(2b) sites, in each model. Note that the multiple peaks in the simulated spectra correspond to different local environments. We have not included the sidebands that would result from these multiple resonances as this would result in even more complex spectra. 33

Figure S24: Comparison of **(a)** simulated and **(b)** observed variable-temperature ^{23}Na NMR spectra for NMMO recorded at 11.7 T under 50 kHz MAS rate. Isotropic resonances in **(b)** are highlighted in bold. 35

Figure S25: Comparison of **(a)** observed and **(b)** simulated variable-temperature ^{23}Na NMR spectra for NMMO recorded at 11.7 T under 28 kHz MAS rate. Isotropic resonances are highlighted in bold. 36

Figure S26: Arrhenius fit of the natural logarithm of the hopping rate, *k*, versus 1000/*T*, to obtain activation energy barriers for Na^+ ion hopping between: **(a)** and **(b)** a P(2d) site and a P(2b) site with one Mn^{4+} and one Mg^{2+} nearest neighbour; **(c)** and **(d)** a P(2d) site and a P(2b) site with two Mn^{4+} nearest neighbours and **(e)** and **(f)** a P(2d) site and a P(2b) site with two Mg^{2+} nearest neighbours. 37

Figure S27: Comparison of **(a)** simulated and **(b)** observed variable-temperature ^{23}Na NMR spectra for NMMO recorded at 16.4 T under 50 kHz MAS rate. Isotropic resonances in **(b)** are highlighted in bold. The simulations include Mn^{3+} bond pathways for the central resonance exchanging sites. 39

Figure S28: Arrhenius fit of the natural logarithm of the hopping rate, *k*, versus 1000/*T*, to obtain activation energy barriers for Na^+ ion hopping between: **(a)** and **(b)** a P(2d) site and a P(2b) site with one Mn^{4+} and one Mg^{2+} nearest neighbour; **(c)** and **(d)** a P(2d) site and a P(2b) site with two Mn^{4+} nearest neighbours and **(e)** and **(f)** a P(2d) site and a P(2b) site with two Mg^{2+} nearest neighbours. 40

Figure S29: Computed ^{23}Na NMR hyperfine (Fermi contact) bond pathways for Mn^{3+} and Mn^{4+} ions in the nearest or next-nearest coordination shell of Na^+ in **(a)** the P(2d) sites and **(b)** the P(2b) sites. Note that Mn^{3+} ions are Jahn-Teller distorted, so pathways may involve individual or combinations of long (L) and short (S) Mn^{3+}–O pathways. Shifts shown are calculated at 318 K, corresponding to a 60 kHz MAS speed. Model used was $Na_{1/2}[Mg_{1/6}Mn^{3+}_{1/6}Mn^{4+}_{2/3}]O_2$ (*a* ≈ 10.20 Å, *b* ≈ 5.10 Å, *c* ≈ 22.86 Å). 41

Figure S30: Comparison between the projected magic angle turning phase adjusted sideband suppression (pjMATPASS) spectrum recorded at 11.7 T and 318 K (60 kHz MAS speed) for NMMO, and bond pathway-based models. In **(a)** and **(b)**, the random and honeycomb-ordered models are compared to the observed spectrum, with a static (middle) and dynamic (bottom) Jahn-Teller (JT) distortion at Mn^{3+}. 47

Figure S31: Comparison between the projected magic angle turning phase adjusted sideband suppression (pjMATPASS) spectrum recorded at 11.7 T and 318 K (60 kHz MAS speed) for NMMO, and bond pathway-based models, zoomed in on the experimentally observed region. In **(a)** and **(b)**, the random and honeycomb-ordered models are compared to the observed spectrum, with a static (middle) and dynamic (bottom) Jahn-Teller (JT) distortion at Mn^{3+}. . 48

Figure S32: Climbing-image NEB results. In **(a)**, the energy profiles for Na^+ ion hops in $Na_{1/2}MnO_2$ and $Na_{1/2}[Mg_{1/6}Mn_{5/6}]O_2$ are shown, with 0% corresponding to the P(2d) site and 100% the P(2b) site. The arrow indicates that, at approximately 80% along the path, Na^+ is already in a P(2b) site; the remainder of the path involves moving directly on top of Mg/Mn. In **(b)**, the local environments of Na^+ at the beginning and end of the hop are shown. 50

S1. Experimental

Synthesis. The synthetic route used was based on previous reports.[1,2] Stoichiometric quantities of Na_2CO_3 (Sigma-Aldrich, 99%), Mn_2O_3 (Aldrich, 99%) and MgO (Acros Organics, 99.99%) were weighed inside an Ar-filled glovebox, removed from the glovebox and ball-milled (Retsch PM 100 planetary ball mill; a zirconia jar with approximately one hundred 3 mm diameter zirconia balls was used whilst milling at 400 rpm) for two hours—consisting of 15-minute milling periods interspersed with 15-minute rests. Batches (approximately 1 – 2 g) of this mixture were heated, in an alumina crucible, at a rate of 10 K min^{-1} to 1073 K under flowing O_2 gas (flow rate approximately 30 mL min^{-1}) and maintained at 1073 K for 10 hours. The powder cooled naturally in the tube furnace for eight hours and was transferred to an Ar-filled glovebox within one minute of removal from the furnace. The black powder was ground by hand in an agate pestle and mortar, removed from the glovebox and returned to the tube furnace under flowing Ar gas (approximately 30 mL min^{-1}). The powder was heated to 973 K at a rate of 10 K min^{-1} under Ar flow and then immediately quenched by bodily shifting the tube so that the crucible was no longer inside the furnace but remained under Ar flow. Once the furnace had cooled to room temperature, the powder was transferred to an Ar-filled glovebox and the powder ground by hand using an agate pestle and mortar. $Na_{0.67}[Mg_{0.28}Mn_{0.72}]O_2$ was collected as a dark brown microcrystalline powder. Throughout this work, all samples were handled and prepared in an Ar-filled glovebox (MBraun, Germany; H_2O and O_2 < 0.1 ppm).

Powder Synchrotron X-ray Diffraction. A powder sample of NMMO was sealed in a borosilicate glass capillary using two-component epoxy resin inside the glovebox. Synchrotron X-ray diffraction (SXRD) patterns were acquired on beamline I11 at the Diamond Light Source.[3,4] The pattern was collected with an exposure time of 1 minute using a position sensitive detector (PSD, Mythen2; X-ray wavelength = 0. 826866 Å) over the range 2θ **=** 2.0 – 92°. Variable-temperature SXRD patterns were also recorded on heating from 100 K to 500 K (heating rate 3 K min^{-1}), with an exposure time of 12 s per pattern (i.e., 0.6 K temperature difference between the start and end of acquisition), using the same PSD and 2θ range.

Powder Total Neutron Scattering. Powder neutron diffraction (PND) measurements were carried out at the ISIS Pulsed Neutron and Muon Source using the GEM instrument.[5] A powder sample of pristine $Na_{0.67}[Mg_{0.28}Mn_{0.72}]O_2$ (3.44 g) was loaded into a thin-walled vanadium canister (8 mm diameter) to a height of 59 mm, to ensure the full beam illuminated the sample. The sample was initially cooled to 130 K, where total scattering data was collected, followed by further cooling to base temperature (1.6 K), another total scattering data set acquired and then a series of diffraction patterns recorded from 2 K up to 7 K in 1 K steps. All data were corrected for absorption effects using the Mantid software package (see main text for data processing methods using GudrunN).[6]

The nuclear structure was determined by Rietveld refinement against powder neutron diffraction data collected above the Néel temperature, T_N, using a model derived from the previously reported powder diffraction structure of $Na_{0.67}[Mg_{0.28}Mn_{0.72}]O_2$. All refinements were carried out using TOPAS Academic 6.0.[7]

All refinements were carried out by simultaneously refining against the pair distribution function data, the diffraction data collected on detector banks 2 to 5 and the SXRD data collected and previously reported.

For refinement against the PND and synchrotron XRD data, the background was fit with a 12-term Chebychev polynomial. The lattice parameters and atomic positions were allowed to refine freely, aside from restraints on the Mn–O (*ca.* 1.85 Å) and Mg–O (*ca.* 2.00 Å) bond lengths. Separate sets of isotropic atomic displacement parameters were freely refined for Na, Mg, Mn and O atoms, whilst the occupancies of Na, Mg and Mn were refined using the

symmetry modes of the irrep and under the constraint that the sample composition was $Na_{0.67}[Mg_{0.28}Mn_{0.72}]O_2$.

Electrochemistry. Electrodes of NMMO were prepared by casting a slurry of NMMO powder, carbon super P (TIMCAL) and Kynar polyvinylidene difluoride (PVDF, Akerma), dispersed in *N*-methyl-2-pyrrolidone (NMP; Sigma-Aldrich, anhydrous, 99.5%), with an active material:carbon:binder mass ratio of 8:1:1. The slurry was blade-coated at a wet film thickness of 500 μm on an aluminium foil current collector. The films were dried at ambient temperature under dynamic vacuum for at least 12 hours. Circular electrodes (13 mm diameter) were punched out and then dried at 393 K for 12 hours under dynamic vacuum; the active material loading was 1.5 – 9.5 mg cm^{-2}.

In all experiments, a 1.0 M $NaPF_6$ (Acros Organics, 98.5+%; dried at 393 K for 12 hours under dynamic vacuum) in propylene carbonate (PC; Solvionic, < 50 ppm H_2O) electrolyte was used. All cells in this work were half-cells, using a Na metal disc as the anode; these discs were punched out from Na metal (Sigma-Aldrich, 99.0%, 13 mm diameter).

All electrochemical tests were conducted using NMMO/Na metal half-cells in 2032 stainless-steel coin cells. Each cell was assembled from a stack of one cathode, one glass fibre separator (Whatman, GF/B, 0.68 mm thick, 16 mm diameter, 1.0 μm pore size) soaked with 150 μL of electrolyte, and one Na metal disc.

Electrochemical measurements were performed using a BioLogic MPG2 potentiostat/galvanostat instrument running EC-Lab software. The NMMO/Na cells were galvanostatically charged and discharged at a rate of 10 mA g^{-1} (corresponding to approximately *C*/19, for a theoretical *C* rate determined from the time elapsed and current applied, assuming that *x* in $Na_x[Mg_{0.28}Mn_{0.72}]O_2$ runs between 0 and 1 and that no parasitic reactions take place during cycling) over a voltage window of 1.5–4.5 V (*vs.* Na/Na^+). A slow cycling rate was chosen to minimise the effect of ionic concentration gradients and large overpotentials often seen at higher cycling rates.

Solid-state Nuclear Magnetic Resonance Spectroscopy. Electrochemically cycled cathodes of NMMO were prepared by cycling a cathode to a given cutoff voltage and allowing the cell to rest for at least one hour. The cell was opened inside an Ar-filled glovebox and the cathode extracted, washed in dimethyl carbonate (DMC; approximately 1 cm^3; Sigma-Aldrich, 99%, anhydrous) and dried *in vacuo* for at least 20 minutes. The cathode was then scraped off the Al foil current collector and either packed into a 1.3 mm diameter ZrO_2 MAS rotor or centre-packed into a 2.5 mm diameter ZrO_2 MAS rotor, using polytetrafluoroethylene (PTFE) tape to fill the gap at either end of the rotor. No rotor spent longer than 10 minutes outside of the glovebox before being inserted into the magnet under a protective atmosphere of flushing nitrogen gas.

The majority of the ^{23}Na NMR spectra were recorded on a Bruker Avance III 11.7 T spectrometer using a Bruker 2.5 mm magic angle spinning (MAS) probe with MAS frequencies of 28 kHz or a Bruker 1.3 mm MAS probe at an MAS rate of between 30 and 60 kHz. In all cases, a rotor-synchronised Hahn-echo pulse sequence (90°–τ–180°–τ–acquire) was used for quantitative measurements and the recycle delay (25 ms; at least $5T_1$) was set such that the bulk, paramagnetically shifted signal was recorded quantitatively, while the diamagnetic signal due to electrolyte decomposition products was suppressed. An effective $\frac{\pi}{2}$ pulse length of 1.15 μs (for the 2.5 mm probe) or 0.67 μs (for the 1.3 mm probe; this corresponds to $\frac{\pi}{6}$, which is longer than the selective $\frac{\pi}{8}$ pulse to ensure all quadrupolar ^{23}Na centres are in the quadrupolar liquid limit;[8] we therefore selected a compromise between the linear quadrupolar regime and maximising the signal intensity). The 2.5 mm ZrO_2 MAS rotor was chosen so that a relatively wide range of temperatures could be accessed; the spinning speed chosen was 28 kHz, as this was sufficient to separate the spinning sideband manifold from its isotropic resonance whilst ensuring rotor stability. Spectra were also recorded on a Bruker Avance III 16.4 T

spectrometer using a Bruker 1.3 mm MAS probe with an MAS frequency of 50 kHz and an effective $\frac{\pi}{2}$ pulse length of 0.58 μs (again corresponding to $\frac{\pi}{6}$). Additional experiments were carried out using a Bruker Avance III 9.4 T spectrometer using a Bruker 1.3 mm MAS probe with an MAS frequency of 35 kHz and an effective $\frac{\pi}{2}$ pulse length of 1.47 μs (again corresponding to $\frac{\pi}{6}$).

All spectra were referenced to solid NaCl at 7.21 ppm and scaled according to the sample mass and number of residuals recorded.

For all experiments, temperature calibration measurements were performed both *ex situ* using the ^{207}Pb shift of $Pb(NO_3)_2$ (Alfa Aesar, 99%) or *in situ* by mixing pristine NMMO powder with KBr (Aldrich, 99% FTIR grade; dried at 473 K for 12 hours under dynamic vacuum) and recording the ^{79}Br spin-lattice T_1 relaxation time. Calibrations were run under the same heater powers, nitrogen gas flow rates, and driving and bearing pressures used for ^{23}Na NMR experiments.

Continuous Wave X-Band Electron Spin Resonance Spectroscopy. Continuous-wave X-band EPR measurements were performed on a Bruker E500 X-band spectrometer with an ER 4122SHQE cavity, tuned to 9.373 GHz. The external magnetic field was modulated to 100 kHz with a modulation amplitude of 0.3 mT. The microwave power was set to 0.6325 mW, sufficient to avoid saturation. The as-synthesised powder (prepared as above) was loaded into a quartz EPR tube (Aldrich, Wilmad® CFQ tubes, outer diameter 2 mm) inside an Ar-filled glovebox and then loaded inside an Oxford Instruments ESR900 cryostat with a temperature stability better than 1.0 K. The sample was first cooled to base temperature (5 K) and spectra recorded on heating at 5 K, 6 K, 7 K, 8 K, 9 K, 10 K, 11 K, 12 K, 13 K, 14 K, 15 K, 20 K, 30 K, 50 K, 80 K, 110 K, 140 K, 170 K, 200 K, 230 K, 260 K and 290 K, with a five-minute equilibration period allowed once temperature stability was reached. EPR spectra were fitted to powder pattern lineshapes with isotropic *g* using the EasySpin Toolbox for MATLAB.[9]

First Principles Calculations. Three model systems were examined for calculation of NMR and Na^+ ion diffusion parameters: firstly, a (2 × 1 × 2) supercell of P2-$Na_{2/3}[Mg_{1/3}Mn_{2/3}]O_2$ (chosen to capture as many exchange interactions as possible) for NMR shifts in a Mn^{4+}-only system; secondly, a (2 × 2 × 1) supercell of P2-$Na_{2/3}[Mg_{1/3}Mn_{2/3}]O_2$ for Na^+ ion hopping barriers (chosen to minimise self-interactions between hopping Na^+ centres) and lastly a (2 × 2 × 1) supercell of P2-$Na[Mg_{1/3}Mn_{2/3}]O_2$ for NMR calculations in a mixed $Mn^{3+/4+}$ system, in which there are equal number of Mn^{3+} and Mn^{4+} centres. Whilst this composition differs from pristine NMMO, it is assumed that the Mn^{3+} and Mn^{4+} bond pathways are independent and therefore additive, based on previous studies.[10,11]

The shifts of each Na site were calculated using methods described previously.[1–4] An initial geometry optimisation was performed using the VASP code,[5–7] employing the projector-augmented wave (PAW) method.[8,9] These calculations used the spin-polarised Perdew-Burke-Ernzerhof (PBE) exchange-correlation functionals, applying the Hubbard *U* model[10,11] within the rotationally invariant formalism proposed by Liechstenstein *et al.*,[12] to correct for known deficiencies of pure functionals for highly localised 3*d* states.[13] The plane-wave energy cutoff was set to 520 eV and an effective Hubbard *U* parameter for the Mn *d* states of $U_{eff} = U - J = 3.9$ eV, where *U* and *J* are the effective on-site Coulomb and exchange parameters (J = 1 eV), respectively, was chosen in line with previous work on the parent material, Na_xMnO_2.[14] SCF cycles were converged with an energy tolerance of 10^{-5} eV. Monkhorst-Pack[15] *k*-point sampling about the gamma point of < 0.5 Å was used in the Brillouin zone.

Periodic spin-polarised DFT calculations were performed in CRYSTAL17.[16] Hyperfine parameters were calculated with B3LYP[17,18] hybrid functionals containing 20% and 35% Hartree-Fock exchange, referred to as Hyb20 and Hyb35, respectively. These weights were chosen based on the success of these functionals in calculating the properties of *TM*

compounds and have been previously reported to provide an upper and lower bound on experimental shifts.[1–3]

The calculations employed two basis sets: a smaller basis set for geometry optimisations (denoted BS-I) and a more extended set for the single-point hyperfine calculations (BS-II). The BS-I sets were taken—without modification—from solid-state studies by Catti *et al.*,[19–22] whilst the BS-II sets comprised bases from the Ahlrichs set for metal ions[23] and IGLO-III basis set for O.[24] The BS-I sets are of the form (15*s*7*p*)/[1*s*3*sp*] for Na, (15*s*3*p*)/[1*s*3*sp*] for Mg, (20*s*12*p*5*d*)/[1*s*4*sp*2*d*] for Mn and (14*s*6*p*)/[1*s*3*sp*] for O. The BS-II sets are of the form (11*s*7*p*)/[7*s*3*p*] for Na, (11*s*7*p*)/[6*s*3*p*] for Mg, (13*s*10*p*5*d*)/[7*s*6*p*3*d*] for Mn and (10*s*6*p*2*d*)/[6*s*5*p*2*d*] for O. In both cases, the values in parentheses denote the number of Gaussian primitives and the values in square brackets denote the contraction scheme.

An initial geometry optimisation on a spin-locked ferromagnetic configuration was run using the BS-I set, with each cell well-converged with an energy tolerance of 10^{-5} AU, a root-mean-square force gradient tolerance of 3×10^{-4} AU and integral thresholds of 10^{-8}, 10^{-8}, 10^{-8}, 10^{-8} and 10^{-16} for the Coulomb overlap, Coulomb penetration, exchange overlap and *g* and *n* series exchange penetration. A subsequent geometry optimisation in which the spins were unconstrained was run, using the spin-locked wavefunction and structure as initial guesses and the same energy tolerances and integral thresholds as the spin-locked calculation. This spin-relaxed geometry-optimised structure was then used in subsequent single-point hyperfine calculations, run using the BS-II basis sets.

A ferromagnetic spin-locked single-point calculation was then carried out; the total energy of each cell was well-converged with a tolerance of 10^{-7} AU and integral thresholds of 10^{-7}, 10^{-7}, 10^{-7}, 10^{-7} and 10^{-14} for the Coulomb overlap, Coulomb penetration, exchange overlap and *g* and *n* series exchange penetration. Finally, another single-point hyperfine calculation was run, starting from the converged spin-locked wavefunction, but this time allowing the spins to relax. The energy tolerances and integral thresholds were the same as in the spin-locked case.

To determine the bond pathways, Mn^{3+} and Mn^{4+} spins were flipped and a single-point hyperfine calculation was then run with the total magnetisation of the cell fixed to give a ferromagnetic state for the first ten SCF cycles, after which no spin constraints were imposed. These runs generated well-converged spin states, again using the same tolerances as before.

Climbing Image Nudged Elastic Band Calculations. The energy barriers to Na^+ ion hopping were determined using the climbing image nudged elastic band (CI-NEB) double-ended transition state searching method implemented in VASP,[34] again employing the PAW method.[18,19] In all cases, a geometry optimisation of the initial and final states was carried out using PBE functionals, with the same U_{eff} parameter and plane-wave energy cutoff as above, but a Gamma-centred *k*-mesh sampling of < 0.2 Å^{-1} and energy tolerance of 10^{-7} eV. To obtain the energy barriers, five linearly interpolated image states were generated between the initial and final states using the VTST packages,[35] and the forces perpendicular to the path connecting them were minimised. Linear interpolation of the forces between these images was then carried out to obtain the energies between each pair of images.

For the P2 and O2 structures, (2 × 2 × 1) supercells were constructed (P2 composition $Na_{16}Mg_8Mn_{16}O_{48}$; O2 composition $Na_{12}Mg_6Mn_{12}O_{36}$). To examine the effect of different Na^+ contents, Na^+ ions were moved from one layer into the other, such that the composition of the cell remained fixed, but the local composition of an individual Na^+ layer varied.

Molecular Dynamics Simulations. The diffusivity of Na^+ ions was obtained from molecular dynamics (MD) simulations, also carried out in VASP. P2 structural models (composition $Na_{16}Mg_8Mn_{16}O_{48}$) obtained from ref. [36] were constructed. To ensure the calculations were affordable, the plane wave kinetic energy cutoff was lowered to 400 eV and Gamma-centred

k-point sampling was kept at < 0.5 $Å^{-1}$. The MD simulations were carried out in the *NVT* ensemble using a Nosé-Hoover thermostat, with a time step of 2 fs. To ensure diffusion could be observed over the timescales used, elevated temperatures were required,[37,38] with simulations being carried out at a range of temperatures from 400 to 800 K. The systems were equilibrated for a period of 5 ps and the simulations then run for 100 ps. Final configurations of MD runs were visually inspected and then optimised to ensure the structure had not changed. The self-diffusion coefficient, D^*, could not be obtained, owing to highly correlated Na^+ ion motion (see main text).

S2. Modelling the effect of Na^+ hopping on the NMR spectra

In P2-type cathodes, Na^+ ions can only hop from P(2d) to P(2b) or *vice versa*—i.e., never from P(2d) to P(2d) or P(2b) to P(2b). As a hop results in a change in the local environment of Na^+ (due to the change in bond pathway between Na^+ and the nearby *TM* cations), the resonant frequency a Na^+ changes during hopping.

The frequency with which Na^+ hops from a P(2d) site to a P(2b) site, $k_{\mathrm{D\rightarrow B}}$, can be written as:

$$k_{\mathrm{d\rightarrow b}} = \omega_0 \exp\left(-\frac{E_{\mathrm{a,D\rightarrow B}}}{k_{\mathrm{B}}T}\right), \tag{1}$$

where ω_0 is the attempt frequency of jumping, (typically ~10^{12} Hz in the solid state[39]), $E_{\mathrm{a,D\rightarrow B}}$ is the activation energy barrier for hopping from the P(2d) site to the P(2b) site, k_{B} is the Boltzmann constant and T the temperature. Similarly, the hop frequency rate for a Na^+ ion going from a P(2b) site to a P(2d) site, $k_{\mathrm{B\rightarrow D}}$, is:

$$k_{\mathrm{b\rightarrow d}} = \omega_0 \exp\left(-\frac{E_{\mathrm{a,B\rightarrow D}}}{k_{\mathrm{B}}T}\right), \tag{2}$$

where $E_{\mathrm{a,B\rightarrow D}}$ is the activation energy barrier for hopping from a P(2b) site to a P(2d) site. These hopping rates may be used in a rate equation to describe the flux of Na^+ ions moving into each of the P(2d) and P(2b) sites:

$$\frac{\mathrm{d[D]}}{\mathrm{d}t} = -k_{\mathrm{D\rightarrow B}}[\mathrm{D}] + k_{\mathrm{B\rightarrow D}}[\mathrm{B}], \tag{3}$$

$$\frac{\mathrm{d[B]}}{\mathrm{d}t} = k_{\mathrm{D\rightarrow B}}[\mathrm{D}] - k_{\mathrm{B\rightarrow D}}[\mathrm{B}], \tag{4}$$

where $[\mathrm{D}]$ and $[\mathrm{B}]$ denote the concentrations of Na^+ ions in the P(2d) and P(2b) sites, respectively, and t is time. Eqs. (3) and (4) may be written in matrix form as:

$$\frac{\mathrm{d}}{\mathrm{d}t}\begin{bmatrix}[\mathrm{D}]\\ [\mathrm{B}]\end{bmatrix} = \begin{bmatrix}-k_{\mathrm{D\rightarrow B}} & k_{\mathrm{B\rightarrow D}}\\ k_{\mathrm{D\rightarrow B}} & -k_{\mathrm{B\rightarrow D}}\end{bmatrix}\begin{bmatrix}[\mathrm{D}]\\ [\mathrm{B}]\end{bmatrix}. \tag{5}$$

The observed NMR lineshape may be computed from the transverse magnetisation, $\widehat{M}^+$, which in the absence of chemical exchange is given by one of the Bloch equations:

$$\frac{\mathrm{d}\widehat{M}^+}{\mathrm{d}t} = (-R_2 + \mathrm{i}\omega)\widehat{M}^+, \tag{6}$$

whose solution is:

$$\widehat{M}^+(t) = \widehat{M}_0^+ \exp\big((-R_2 + \mathrm{i}\omega)t\big), \tag{7}$$

where $\widehat{M}_0^+$ is the initial magnetisation (which is proportional to the number of sites), R_2 is the transverse relaxation rate and ω is the resonant frequency of the nucleus.

On introducing multiple sites, the total magnetisation function of the system, $\widehat{M}_{\mathrm{tot.}}^+ = \widehat{M}_{\mathrm{D}}^+ + \widehat{M}_{\mathrm{B}}^+$, must be considered. To account for chemical exchange, the Bloch equation (eq. (6)) must also be modified, so that in the absence of chemical exchange:

$$\frac{\mathrm{d}}{\mathrm{d}t}\begin{bmatrix}\widehat{M}_{\mathrm{D}}^+(t)\\ \widehat{M}_{\mathrm{B}}^+(t)\end{bmatrix} = \begin{bmatrix}-R_{2,\mathrm{D}} + \mathrm{i}\omega_{\mathrm{D}} & 0\\ 0 & -R_{2,\mathrm{B}} + \mathrm{i}\omega_{\mathrm{B}}\end{bmatrix}\begin{bmatrix}\widehat{M}_{\mathrm{D}}^+\\ \widehat{M}_{\mathrm{B}}^+\end{bmatrix}, \tag{8}$$

where $\widehat{M}_{\mathrm{D}}^{+}$ and $\widehat{M}_{\mathrm{B}}^{+}$ are the magnetisations of Na^{+} ions in the P(2d) and P(2b) sites.

On introducing chemical exchange to the system, the Bloch equations become the Bloch-McConnell equations:[40]

$$\frac{\mathrm{d}}{\mathrm{d}t}\begin{bmatrix}\widehat{M}_{\mathrm{D}}^{+}(t)\\ \widehat{M}_{\mathrm{B}}^{+}(t)\end{bmatrix} = \left(\begin{bmatrix}-R_{2,\mathrm{D}}+\mathrm{i}\omega_{\mathrm{D}} & 0\\ 0 & -R_{2,\mathrm{B}}+\mathrm{i}\omega_{\mathrm{B}}\end{bmatrix} + \begin{bmatrix}-k_{\mathrm{D}\to\mathrm{B}} & k_{\mathrm{B}\to\mathrm{D}}\\ k_{\mathrm{D}\to\mathrm{B}} & -k_{\mathrm{B}\to\mathrm{D}}\end{bmatrix}\right)\begin{bmatrix}\widehat{M}_{\mathrm{D}}^{+}(t)\\ \widehat{M}_{\mathrm{B}}^{+}(t)\end{bmatrix}, \quad (9)$$

the solution to which is:

$$\begin{bmatrix}\widehat{M}_{\mathrm{D}}^{+}(t)\\ \widehat{M}_{\mathrm{B}}^{+}(t)\end{bmatrix} = \begin{bmatrix}a_{11}(t) & a_{12}(t)\\ a_{21}(t) & a_{22}(t)\end{bmatrix}\begin{bmatrix}\widehat{M}_{\mathrm{D}}^{+}(0)\\ \widehat{M}_{\mathrm{B}}^{+}(0)\end{bmatrix}, \quad (10)$$

where $\widehat{M}_{\mathrm{D}}^{+}(0)$ and $\widehat{M}_{\mathrm{B}}^{+}(0)$ are the initial magnetisations of the P(2d) and P(2b) sites, respectively. These quantities are directly proportional to the probability of occupying these sites; in the present model, this is determined from the relevant thermodynamic (Boltzmann) probabilities, using the differences in energy levels as the relative energies of each state.

The elements of the matrix $\boldsymbol{a}$ are:

$$a_{11} = \frac{1}{2}\left[\left(1-\frac{\mathrm{i}\Delta\omega-\Delta R_2+k_1-k_{-1}}{\lambda_+-\lambda_-}\right)\exp(-\lambda_- t)+\left(1+\frac{\mathrm{i}\Delta\omega-\Delta R_2+k_1-k_{-1}}{\lambda_+-\lambda_-}\right)\exp(-\lambda_+ t)\right],$$

$$a_{12} = \frac{k_{-1}}{(\lambda_+-\lambda_-)}[\exp(-\lambda_- t)-\exp(-\lambda_+ t)],$$

$$a_{21} = \frac{k_{1}}{(\lambda_+-\lambda_-)}[\exp(-\lambda_- t)-\exp(-\lambda_+ t)],$$

$$a_{22} = \frac{1}{2}\left[\left(1+\frac{\mathrm{i}\Delta\omega-\Delta R_2+k_1-k_{-1}}{\lambda_+-\lambda_-}\right)\exp(-\lambda_- t)+\left(1-\frac{\mathrm{i}\Delta\omega-\Delta R_2+k_1-k_{-1}}{\lambda_+-\lambda_-}\right)\exp(-\lambda_+ t)\right],$$

where $\Delta\omega = \omega_{\mathrm{B}}-\omega_{\mathrm{D}}$, $\Delta R_2 = R_{2,\mathrm{B}}-R_{2,\mathrm{D}}$ and λ_+ and λ_- are the eigenvalues of eq. (9):

$$\lambda_{\pm} = \frac{1}{2}\left[\left(-\mathrm{i}\omega_{\mathrm{D}}-\mathrm{i}\omega_{\mathrm{B}}+R_{2,\mathrm{D}}+R_{2,\mathrm{B}}+k_1+k_{-1}\right)\pm\sqrt{(\mathrm{i}\Delta\omega-\Delta R_2+k_1-k_{-1})^2+4k_1k_{-1}}\right].$$

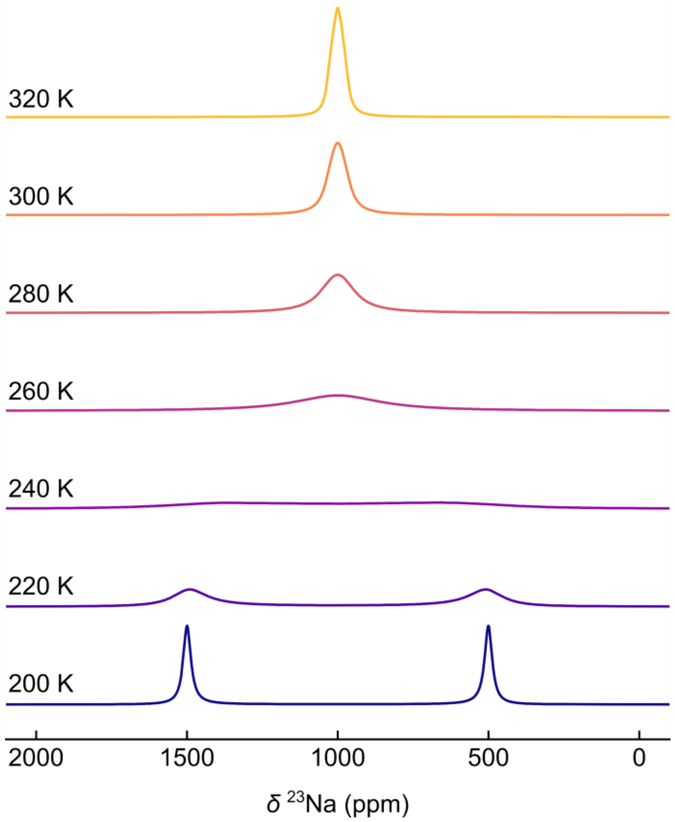

Figure S1: demonstration of the exchange simulation script. The distinct resonant frequencies were chosen as 500 ppm (henceforth site A) and 1500 ppm (henceforth site B), with forward and reverse energy barriers of hopping of 310 meV (chosen to be equal for the sake of simplicity). A hopping attempt frequency of 10^{12} Hz was chosen to represent that in a typical solid. The temperature ranges from 200 K (bottom) to 320 K (top).

Therefore, a spectrum may be extracted *via*:

$$\mathrm{Spectrum} = \mathrm{FT}\left[\left(a_{11}(t) + a_{21}(t)\right)\widehat{M}_{\mathrm{D}}^{+}(t) + \left(a_{12}(t) + a_{22}(t)\right)\widehat{M}_{\mathrm{B}}^{+}(t)\right]. \qquad (11)$$

In other words, a spectrum can be obtained by providing (to this model) a pair of sites whose chemical shifts are known and the hopping barrier between them is also known. This produces the expected results for each regime of ionic hopping, as seen in Figure S1. Here, eqs. (10) and (11) are used to simulate the ^{23}Na NMR spectra of NMMO at a range of temperatures between 250 K and 376 K and compare our simulations to the experimentally observed spectra. Note that the above spectrum yields only the isotropic shift and neglects the effect of motion or of the quadrupolar interaction on the sideband manifold; such a treatment is beyond the scope of this work. We note also that Curie-Weiss scaling used in paramagnetic NMR holds in the fast, slow and intermediate exchange regimes. In the fast intermediate exchange regime, the isotropic shift will still be centred at the thermodynamic average of the two sites between which Na^+ hops, albeit with a much faster T_2. In the slow intermediate exchange regime, the isotropic shifts will remain approximately equal to the shifts of the individual sites. In each case, the ^{23}Na shift is still Curie-Weiss scaled as this scaling encapsulates the magnitude of the time-averaged electron spin moment that interacts with the nuclear spins. The Curie-Weiss scaling will break down as the sample temperature approaches the size of the net exchange interaction (i.e., $T \sim \theta$), as the Curie-Weiss law only models the paramagnetic regime. For NMMO, this regime only occurs below around 50 K.

S3. Variable-temperature NMR temperature calibration

To determine the sample temperature for variable-temperature ^{23}Na NMR experiments, the ^{207}Pb shift in $Pb(NO_3)_2$, the ^{79}Br T_1 relaxation time in KBr and the ^{79}Br shift in KBr was determined, as these materials are known "NMR thermometers".[41,42] The choice of which thermometer was (somewhat) arbitrary, but choosing whether the shift or relaxation time was based on the length of T_1 and how quickly good signal-to-noise could be acquired: long T_1s are seen at low temperature, so a measurement of T_1 was chosen rather than a Hahn-echo, whilst short T_1s occur at higher temperatures, making the Hahn-echo more appropriate and a faster sequence. In all cases, a calibration curve was constructed to convert the probe temperature to a true sample temperature. These calibration curves are given in Figure S2 and Figure S3. Note that the temperature of the sample measured at 100 K was obtained from a single measurement of the ^{79}Br T_1 *in situ*.

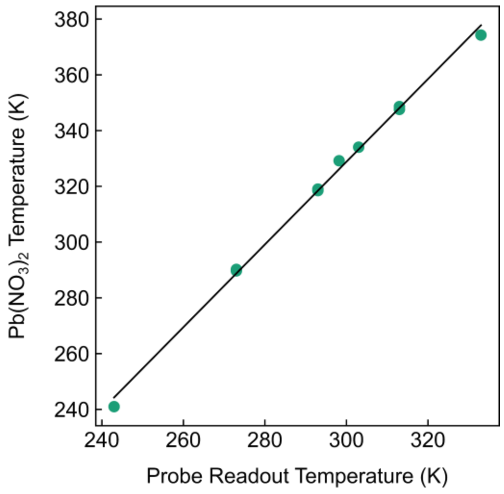

Figure S2: *Ex situ* variable-temperature NMR temperature calibration curve, obtained from the ^{207}Pb shift of $Pb(NO_3)_2$

.

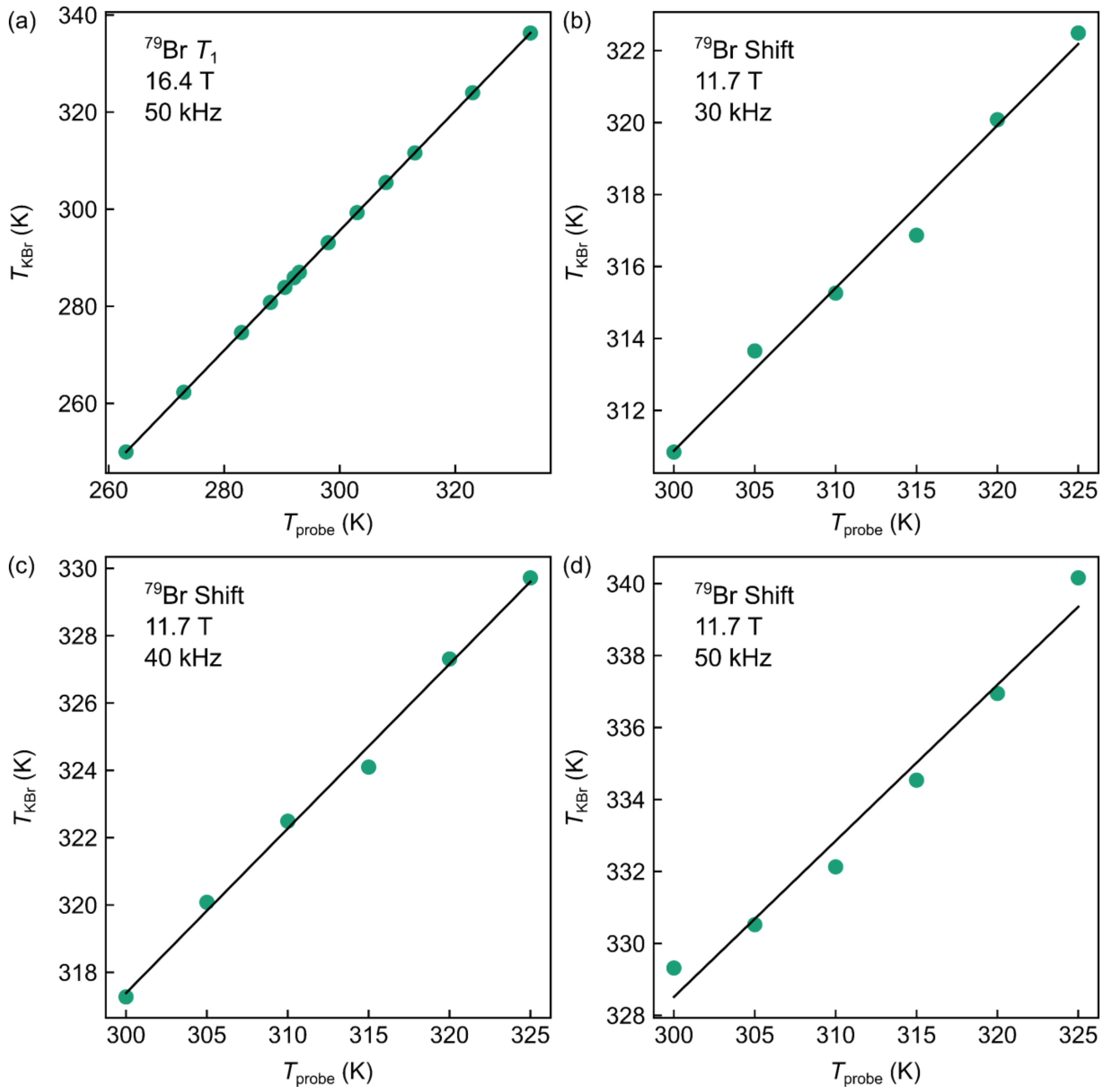

Figure S3: *In situ* variable-temperature NMR temperature calibration curves, obtained from the ^{79}Br T_1 time or shift of KBr. **(a)** shows the curve obtained using the T_1 time as a metric for the temperature, using a field of 16.4 T and an MAS rate of 50 kHz; **(b)**, **(c)** and **(d)** show the curves for the ^{79}Br shift at 11.7 T under 30 kHz, 40 kHz and 50 kHz MAS, respectively. Black lines show the fitted straight lines to each set of data points (green).

S4. Additional SXRD and Neutron Refinements

Refining for Superstructure Peaks

To assess the effect of Na^+ displacement modes on the diffraction pattern observed, a Rietveld refinement on only the superstructure regions Q = 0.70 – 0.95 $Å^{-1}$ and Q = 1.75 – 2.25 $Å^{-1}$ was carried out [Figure S4(a) and (b)]. These show a good fit to the superstructure peaks in these regions, but a poorer fit to the rest of the pattern, with some peaks acquiring too much intensity. It is therefore likely that more than one irrep can be used to describe the structural distortion: perhaps one for the *TM* sublattice and one for the Na sublattice. Attempts to generate structures with two irreps active using ISODISTORT were unsuccessful, due to the large number of possible combinations of displacements.

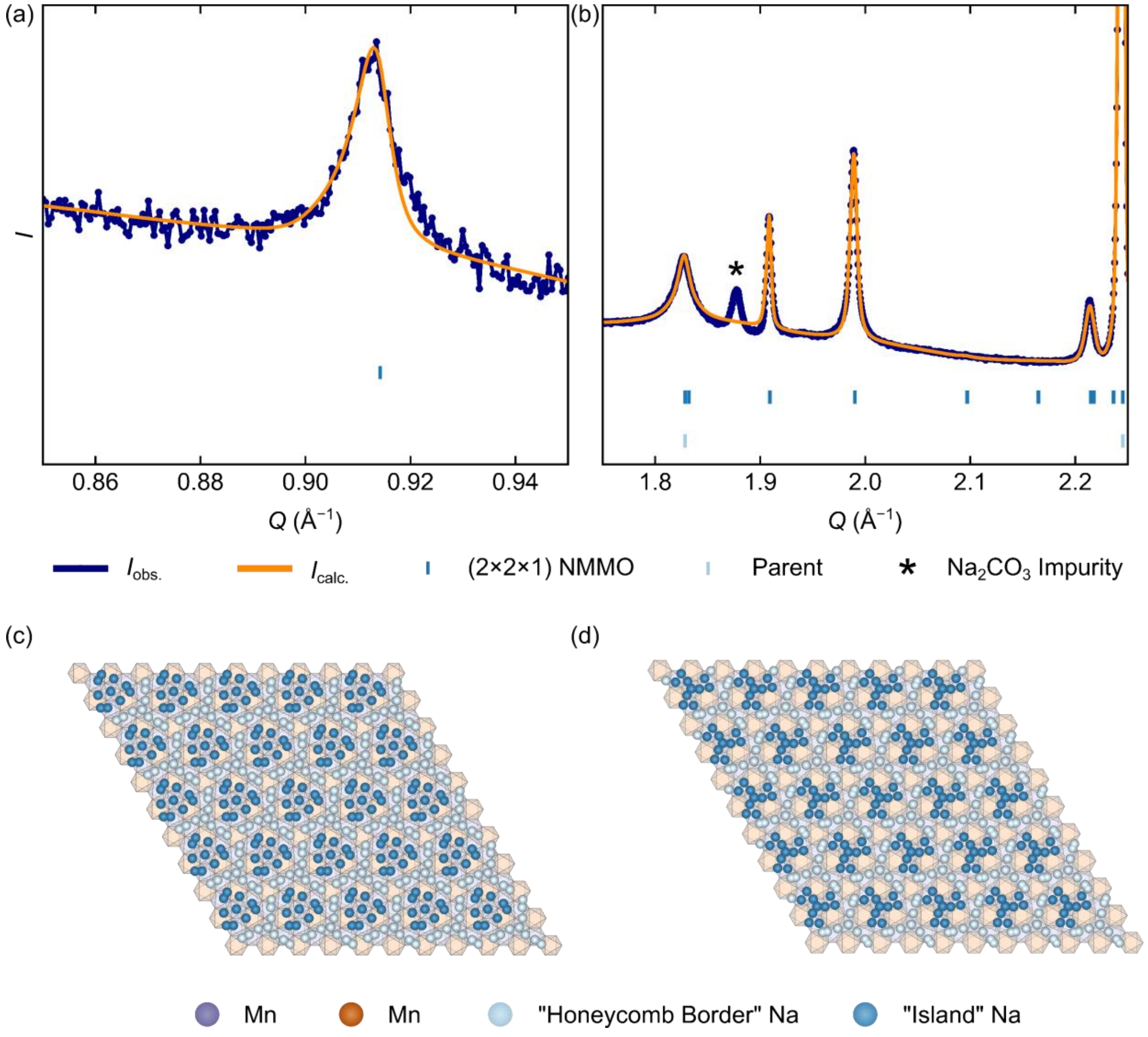

Figure S4: Rietveld refinements of SXRD data for NMMO at room temperature, focussing on the superstructure peaks. **(a)** and **(b)** show the refinement in the regions Q = 0.70 – 0.95 $Å^{-1}$ and Q = 1.75 – 2.25 $Å^{-1}$, respectively. In **(c)** and **(d)**, the structures of the superstructure from the main text and the refined superstructure from the refinement in **(a)** are shown, respectively.

Variable-Temperature PND Refinements

Additional powder neutron diffraction (PND) patterns were acquired at: 1.6 K, 2 K, 3 K, 4 K, 5 K, 6 K, 7 K, 10 K, 20 K, 40 K, 130 K and 295 K. The patterns change very little with temperature, save small changes in the peak positions. Refinements to these patterns are available on request. The refinement to the room temperature data in detector banks 3 to 5 is shown below [Figure S5(b) to (d)].

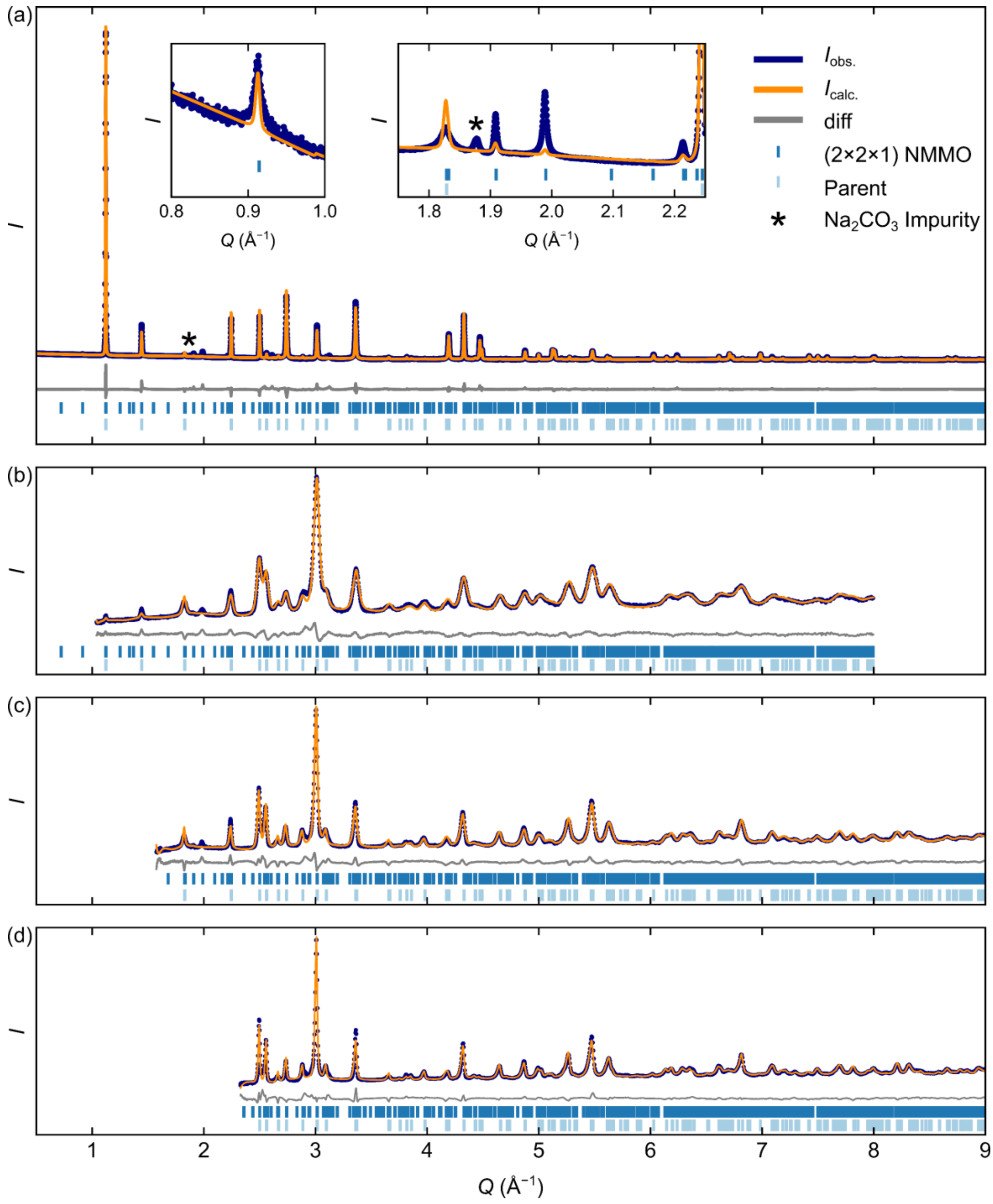

Figure S5: Rietveld refinements of both the parent and (2 × 2 × 1) superstructure to **(a)** synchrotron X-ray diffraction and **(b)** to **(d)** powder neutron diffraction patterns banks 3 to 5 respectively; $R_{w.p.}$ 3.93 %. Insets in **(a)** show some of the superstructure reflections in the regions Q = 0.85 – 0.95 Å^{-1} and Q = 1.75 – 2.25 Å^{-1}.

Refinement of the parent structure only

To further show that the superstructure presented in the main text is required to capture the structure of NMMO, we performed refinements to only the parent (previously reported) structure, as shown in Figure S6 and Figure S7. These results indeed show a poorer fit to the experimental data when only the parent is included.

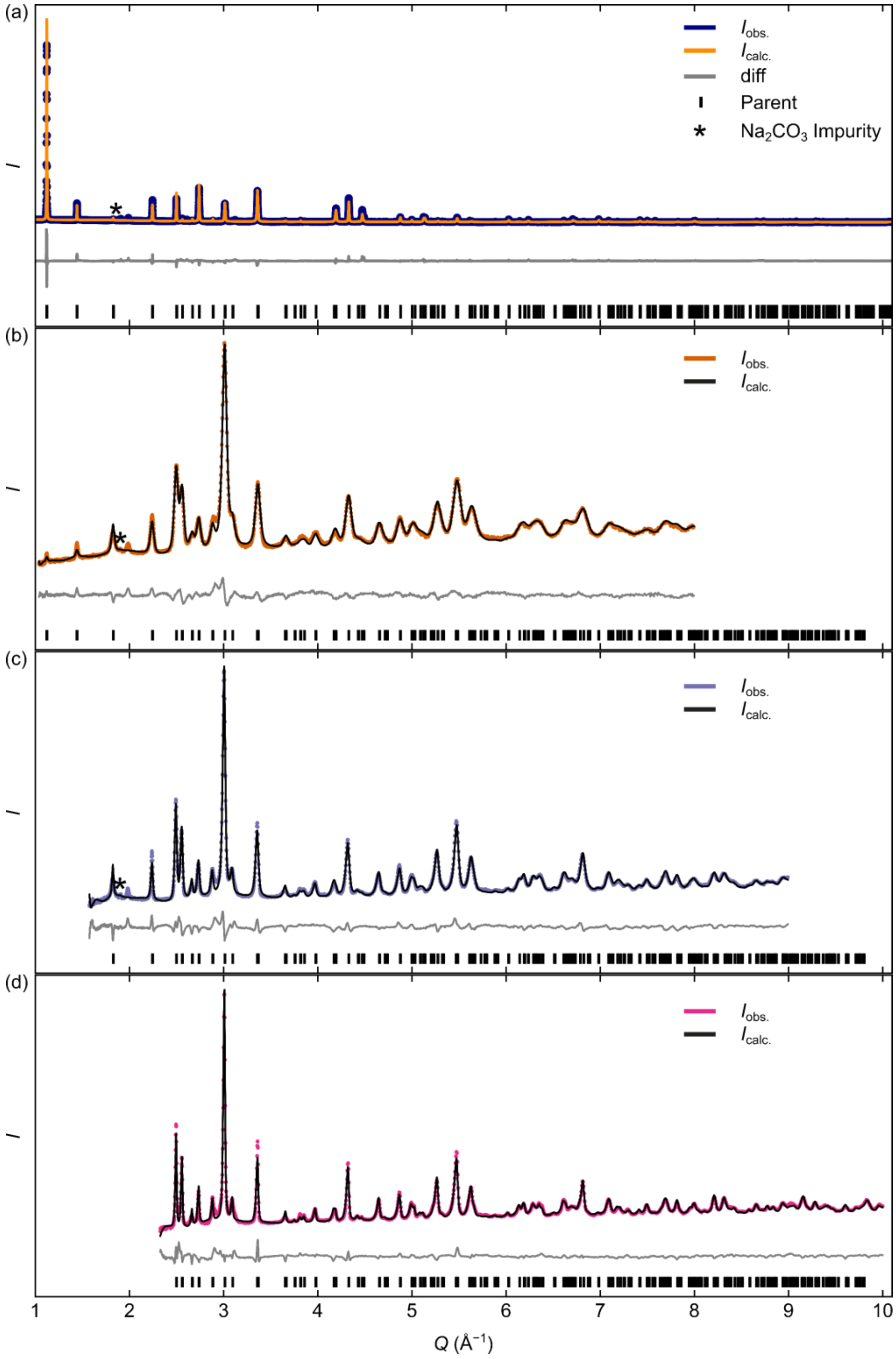

Figure S6: Rietveld refinements of the parent structure against **(a)** the room temperature synchrotron X-ray diffraction data, and **(b)**, **(c)** and **(d)** the room temperature neutron diffraction data for banks 3 – 5; $R_{w.p.}$ = 14.7%

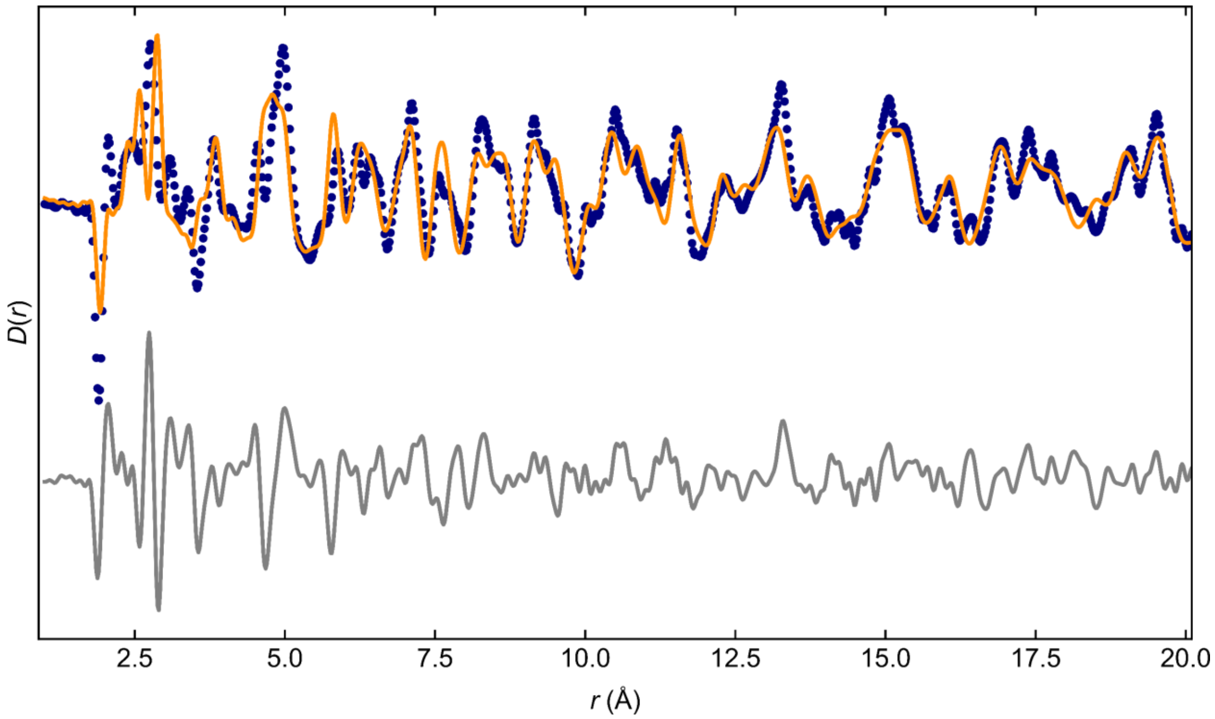

Figure S7: Neutron pair distribution function data for NMMO at room temperature, with fit to the parent structure only.

Changing the Mg/Mn ordering

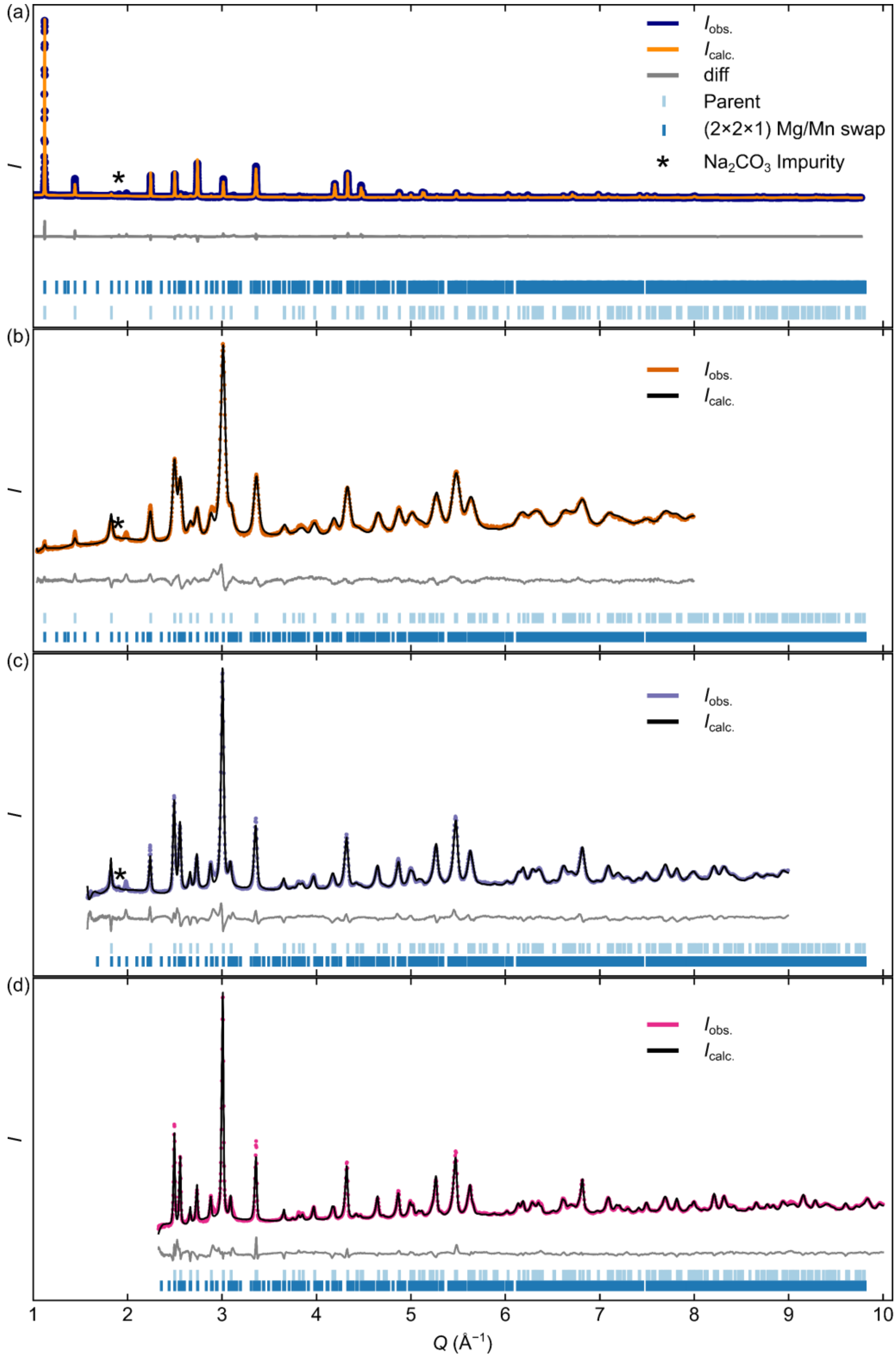

Figure S8: Rietveld refinements of the parent and (2×2×1) superstructure with Mn and Mg occupancies swapped relative to the superstructure presented in the main text against **(a)** the room temperature synchrotron X-ray diffraction data, and **(b)**, **(c)** and **(d)** the room temperature neutron diffraction data for banks 3 – 5; $R_{w.p.}$ = 8.9%

In addition, we carried out refinements to a (2 × 2 × 1) superstructure in which the Mg and Mn positions are swapped, to indicate whether the positions of Mg and Mn in the *TM* sublattice were consistent with Mg at the corners, centres of faces and centres of edges of the unit cell and Mn in the remaining positions (as described in the main text) or if Mn at the corners, centres of faces and centres of edges of the unit cell and Mg in the remaining sites gave similar or better results. As shown in Figure S8, the fit to swapping Mn and Mg is poorer, suggesting the reported ordering in this work is consistent with the observed data.

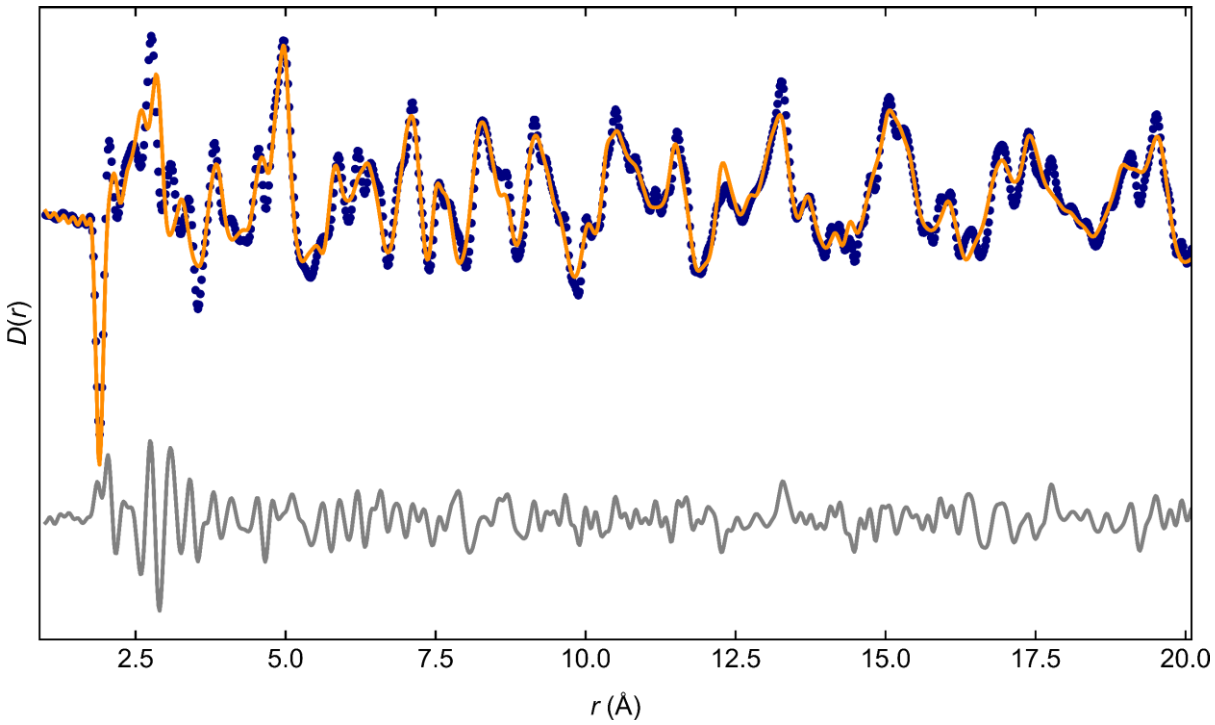

Figure S9: Neutron pair distribution function data for NMMO at room temperature, with Rietveld fit to the superstructure with Mg and Mn swapped.

Diffuse Magnetic Neutron Scattering

Since neutrons are sensitive to the magnetic moments in materials, neutron scattering offers a chance to observe the magnetic ground state of a material. If the ground state has an ordered structure, magnetic Bragg diffraction is seen, but if the magnetic ground state is disordered, magnetic diffuse scattering is seen. In the case of NMMO, diffuse scattering was seen near Q = 0.5 Å^{-1} below 40 K, suggesting that this excitation occurs over a number of spins ($d \rightarrow$ 12.5 Å) and involves little energy to achieve and excitation [Figure S10]; we note also that the Neél temperature, T_N, is 6.5 K (from previous work[25]). The d-spacing for this excitation corresponds to approximately five Mn–Mn distances in NMMO, indicating that spin clusters over five Mn centres are generated at low temperatures in NMMO. These observations are consistent with spin glass materials, where several states lie very close in energy and the spin orientation have only short-range order, but no long-range order.[43–45]

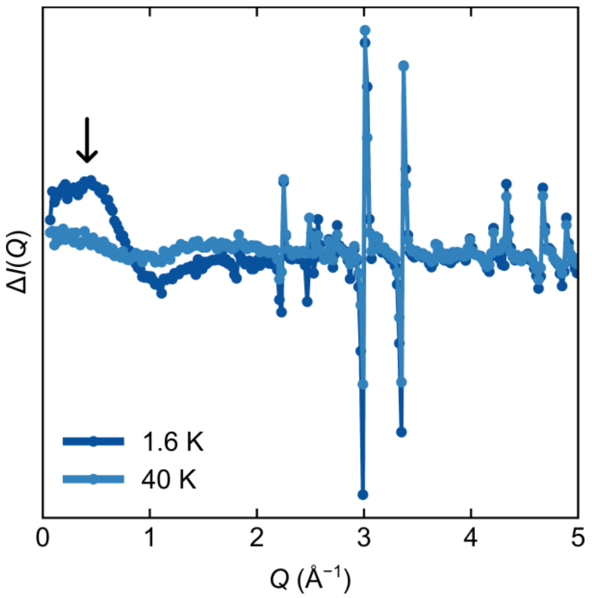

Figure S10: Difference in the total neutron scattering, $\Delta I(Q)$, of NMMO at 1.6 K and 40 K, relative to the scattering at 130 K, revealing diffuse magnetic scattering near Q = 0.5 Å^{-1} (arrowed).

Additional information about the Na^+ ion hopping rates may be extracted from assessing the subtle changes to the SXRD patterns acquired at variable temperature [Figure S11]. Tentatively, the following may be concluded: on average, Na^+ ions spend marginally more time in the P(2d) environments at low temperatures than at high temperatures, consistent with the idea that P(2d) sites are lower in energy than P(2b) due to the lower Coulombic repulsion in these sites. This trend was not seen in the parent structure, perhaps suggesting a higher average energy barrier to Na^+ ion hopping in the parent structure than in the (2 × 2 × 1) superstructure, resulting in more kinetic trapping of Na^+ ions in the parent structure. Despite providing only tentative conclusions about the Na^+ ion site preferences and relative mobilities, the variable- temperature refinements can provide useful information about Na^+ ion mobility from the displacement parameters for Na^+. The SXRD patterns show only minor changes with temperature; the most distinctive change to the pattern, however, is the decrease in intensity of the (204) reflection at Q = 2.67 Å^{-1} above approximately 350 K [Figure S11(a)]. Simulations of the diffraction pattern in ISOVISQ [Figure S11(b)] revealed that this reflection is overwhelmingly dominated by P(2d) Na centres in the hexagonal border of the Na^+ ion array and is primarily affected by the GM_1^+ and M_2^+ displacement modes, in which some P(2b) Na^+ ions (specifically Na2_2 and Na2_3) with two Mn nearest neighbours hop towards P(2d) sites (Na1_1 and Na1_2, Figure S11(b)). The disappearance of this reflection is illustrative of how Na^+ ordering schemes and mobility affect the intensities in SXRD.

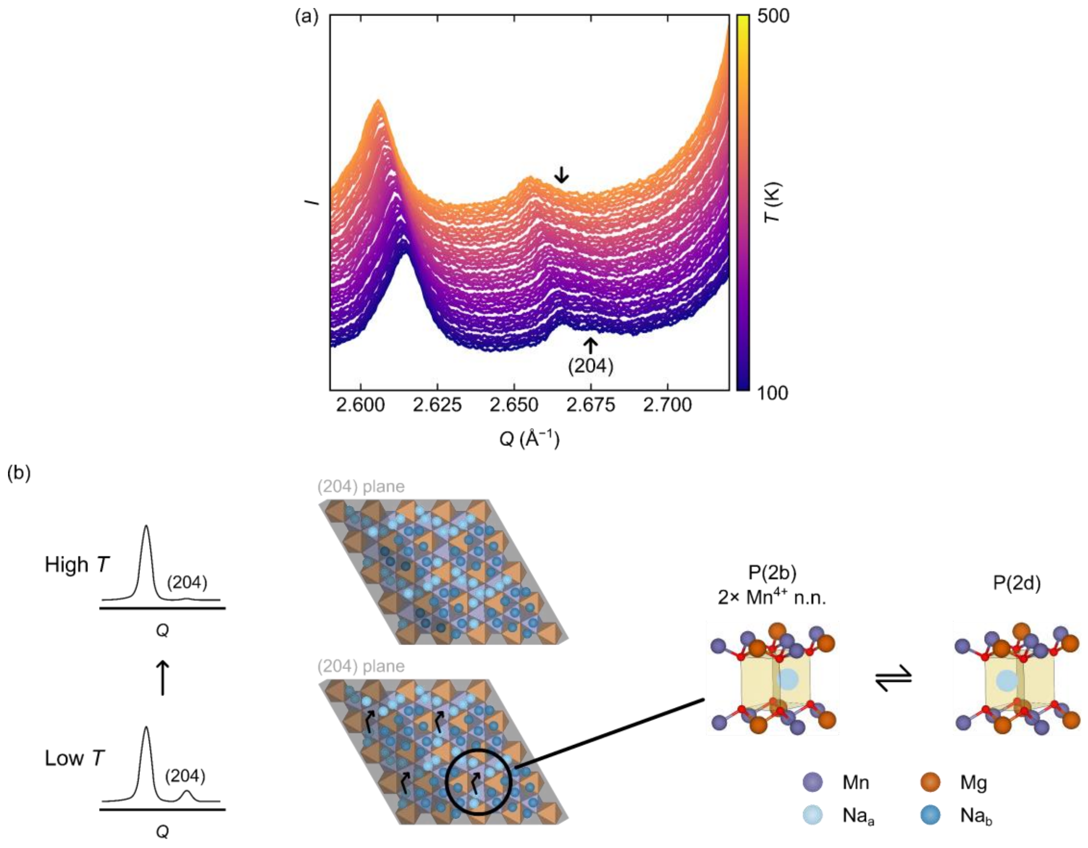

Figure S11: (a) shows an expansion of the variable-temperature SXRD patterns between Q = 2.59 Å^{-1} and Q = 2.72 Å^{-1}, corresponding to the (204) reflection, as indicated. In **(b)**, the simulated effect of the GM_1^+ and M_2^+ displacive modes on the diffraction pattern is highlighted: when the arrowed Na^+ ions hop away from the plane from a P(2b) site with two Mn^{4+} nearest neighbours to a P(2d) site, the (204) reflection decreases in intensity (right). Note that the shading of the (204) plane indicates Na^+ centres which are above (lighter colour) and below (darker colour) the (204) plane.

S5. Bond Valence Sum Analysis

To assist the understanding of Na^+ mobility in NMMO, we computed the bond valence sum maps using the refined crystal structures obtained from SXRD [Figure S12]. This indicates a higher mobility for the Na_a-type sites compared to the Na_b-type sites, as anticipated; it also indicates s small amount of exchange between the outermost sites of Na_b regions with Na_a.

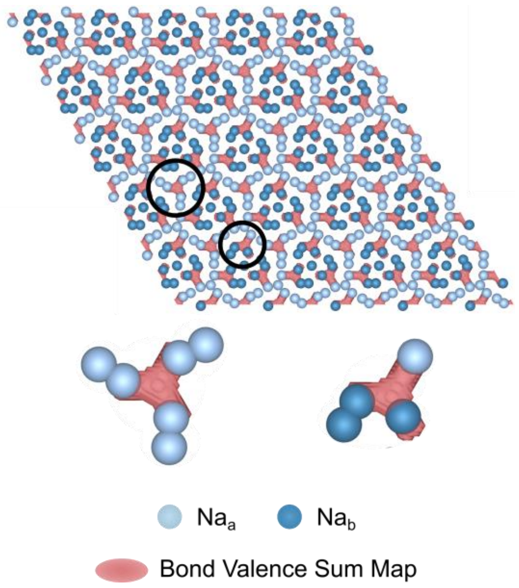

Figure S12: Computed bond valence sum map for NMMO. For clarity, only the Na layer is shown.

S6. Magnetic Exchange interactions and EPR Weiss constant fits

The superexchange and direct exchange interactions are generally spatially isotropic and can be ferro- or antiferromagnetic, depending on the orbital overlap between two magnetic centres. The signs and strengths of these exchange interactions were first rationalised and predicted by Goodenough[129] and Kanamori[130,131] by considering virtual electron hops between orbitals, such that electrons were delocalised from one orbital to another (if there is at least one vacant orbital in the overlap pathway; Figure S13, middle) and/or polarised according to Hund's rules (if the orbitals in the overlap pathway are filled; Figure S13, top and bottom).

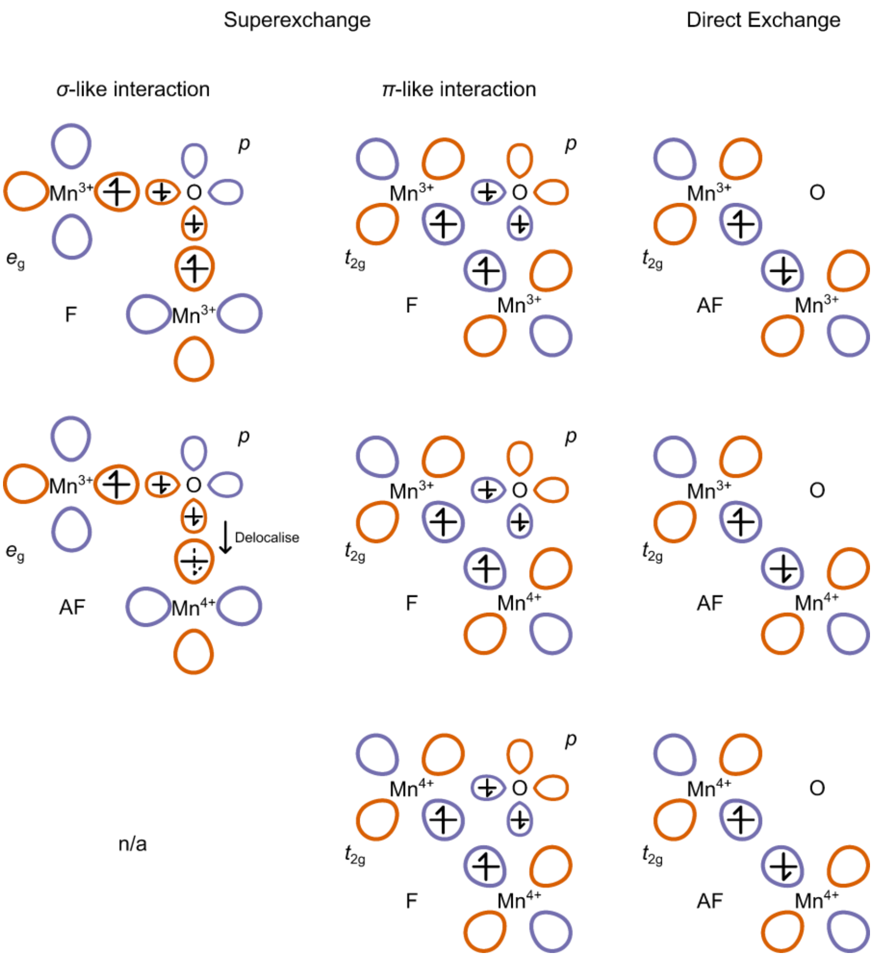

Figure S13: Schematics showing the exchange interactions between Mn^{3+} and Mn^{4+} centres in NMMO, labelled ferromagnetic (F) and antiferromagnetic (AF).

As stated in the main text, the variation in EPR signal intensity as a function of temperature may be modelled using the Curie-Weiss law, as the EPR signal intensity is directly proportional to the static magnetic susceptibility of the sample. Fits of the total signal, and of the separate intensities of the fitted resonances, A and B, are shown in Figure S14. The Weiss constant obtained for the overall intensity was −36 K, while A and B gave Weiss constants of −41 K and −18 K, respectively.

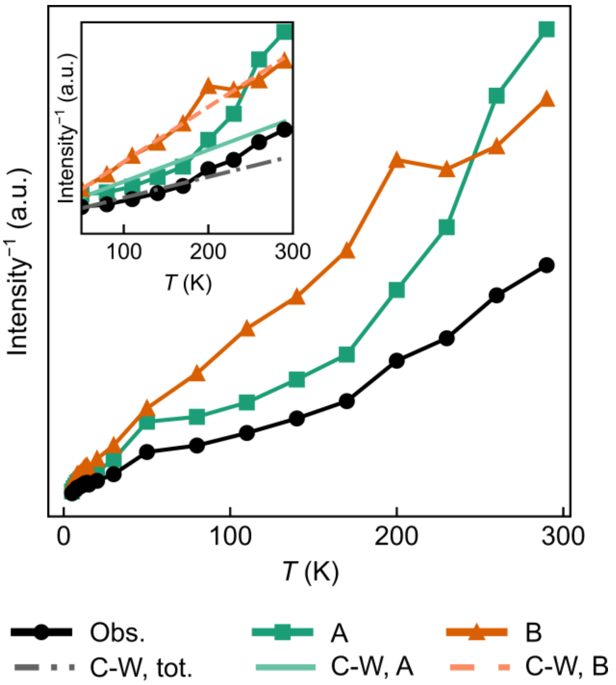

Figure S14: Observed and fitted (resonances A and B) inverses of EPR intensities as a function of temperature. Inset shows the fit to the paramagnetic regime, above 80 K.

S7. Additional variable-temperature ^{23}Na NMR results

Determining the isotropic resonance(s)

To identify the isotropic resonances present in the Hahn-echo spectra, we carried out projected magic-angle-turning phase-adjusted sideband-separation (pjMATPASS) experiments at both 40 kHz (sample temperature 296 K) and 60 kHz (318 K); the projection of the isotropic slice is shown in Figure S15 below, on top of the Hahn-echo spectra acquired. Note that the pjMATPASS and Hahn-echo spectra have been scaled to match the intensities of the isotropic resonances to make identification of these signals more straightforward. Knowing these, we performed a simple linear interpolation of the shifts using Curie-Weiss scaling to estimate the shifts at all other temperatures. These predicted shifts aligned with observed peaks, which were in turn assigned as the isotropic components.

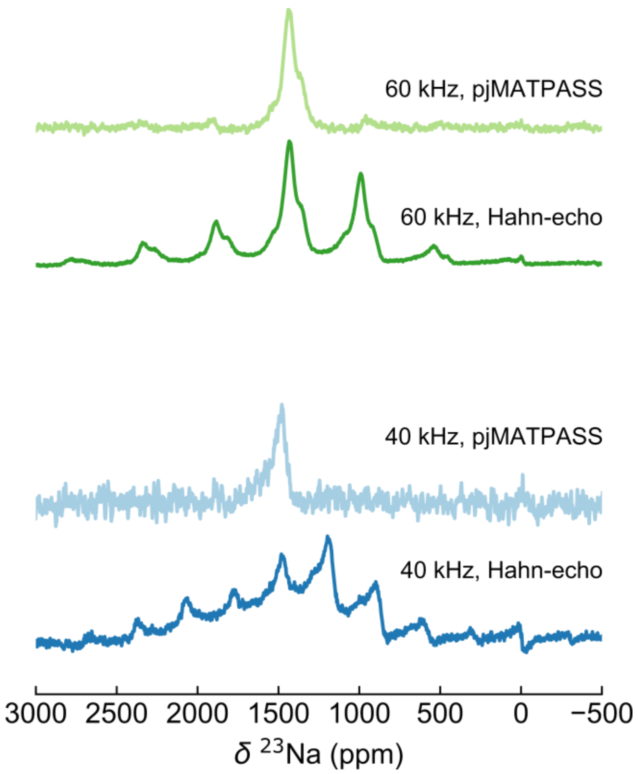

Figure S15: Hahn-echo and isotropic projection of pjMATPASS spectra acquired at 60 kHz and 40 kHz MAS speed, under an applied field of 11.7 T

Data at 11.7 T under 50 kHz MAS

In addition to the variable-temperature ^{23}Na NMR spectra acquired at 16.4 T, variable-temperature data was also acquired at 11.7 T, both at 50 kHz [Figure S16] and at 28 kHz MAS [Figure S17]. These data showed the same trends as that seen at 16.4 T: a decrease in isotropic shift and the gradual sharpening and increase in intensity of resonances at high temperatures.

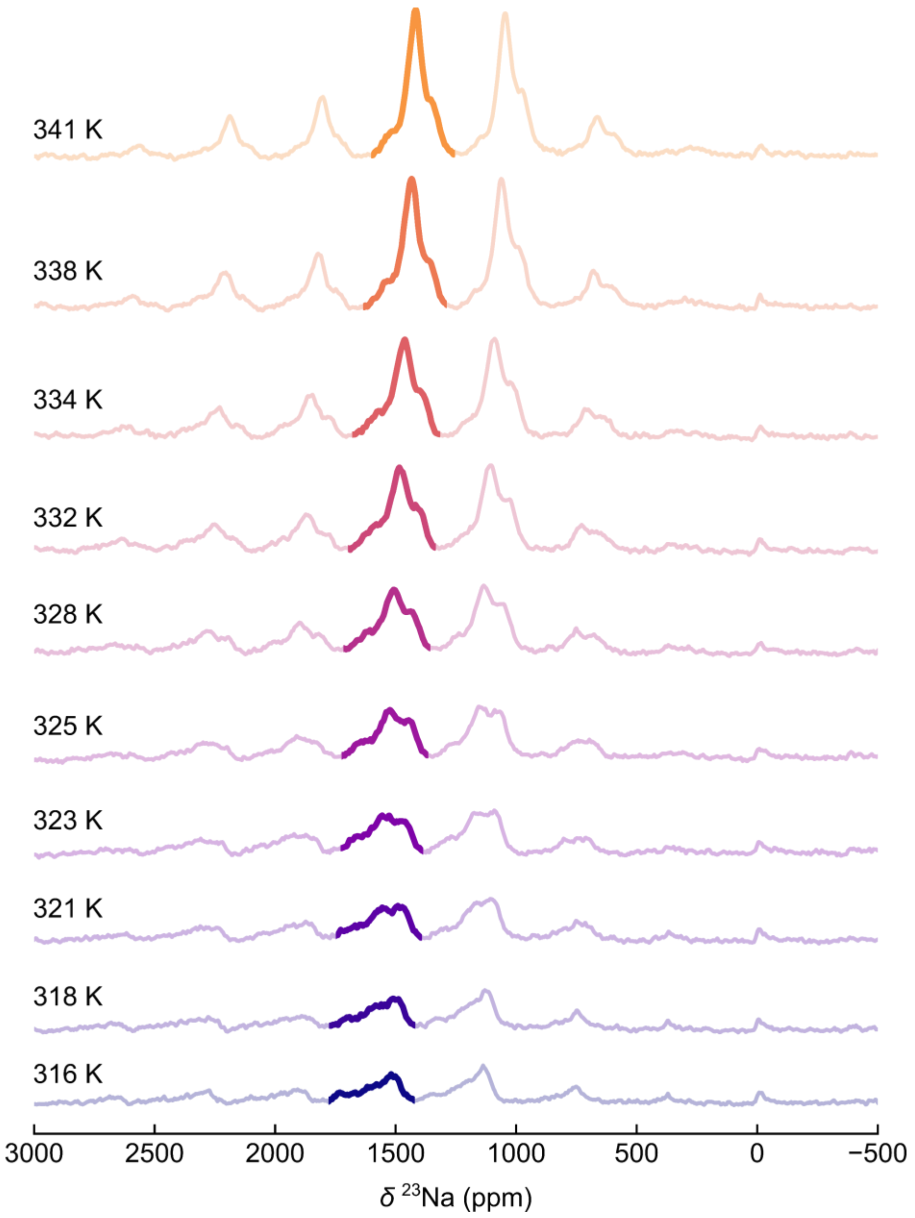

Figure S16: Variable-temperature ^{23}Na NMR spectra for NMMO recorded at 11.7 T under 50 kHz MAS rate. Isotropic resonances are highlighted in bold.

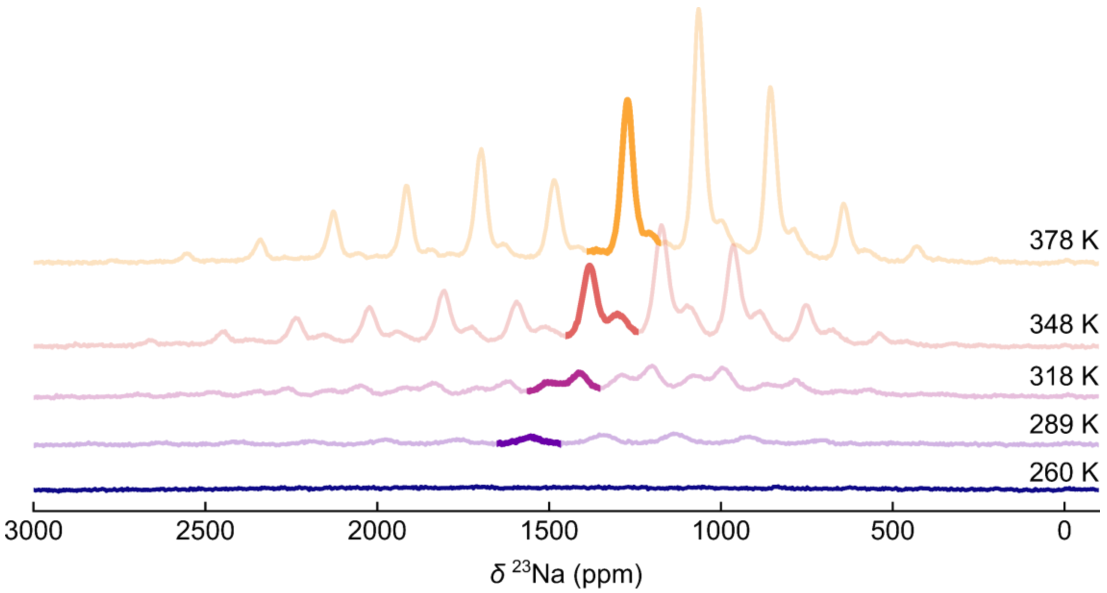

Figure S17: Variable-temperature ^{23}Na NMR spectra for NMMO recorded at 11.7 T under 28 kHz MAS rate. Isotropic resonances are highlighted in bold.

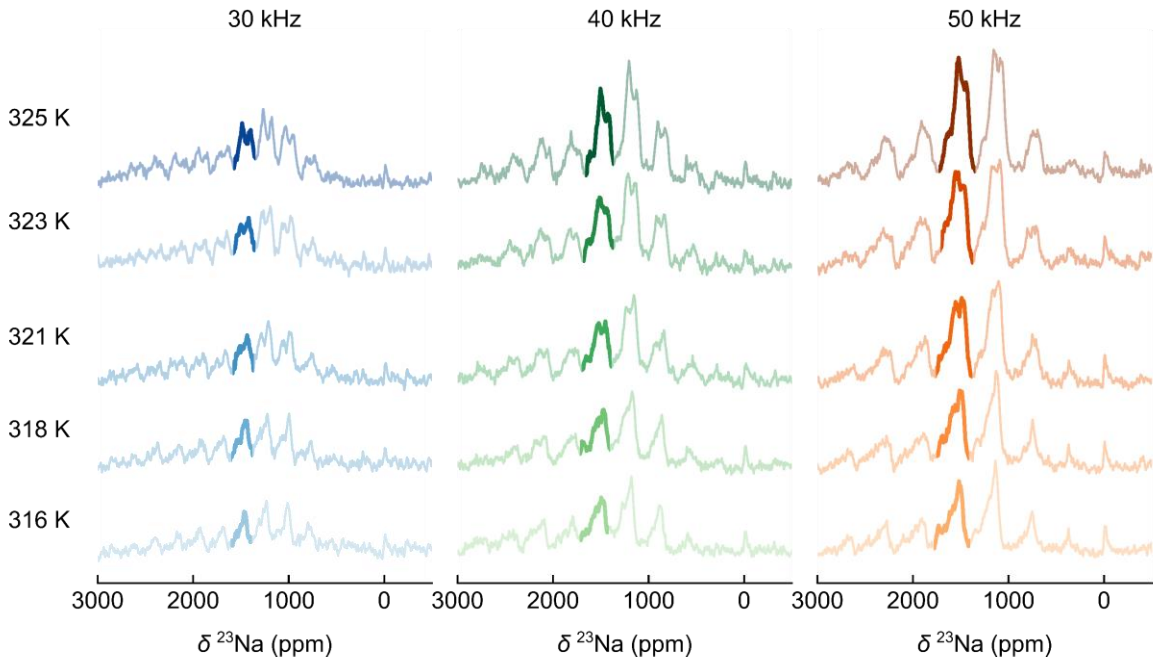

Figure S18: Fixed-sample-temperature, variable MAS frequency ^{23}Na NMR spectra acquired for P2-NMMO at 11.7 T, with isotropic resonances highlighted in bold. Spectra are scaled according to number of scans and mass of sample.

Effect of T_2 on spectra

Individual slices from T_2 experiments, in which the echo delay incremented along the indirect dimension, were recorded to identify differences in T_2s of the three isotropic peaks [Figure S19]. This highlights the shortened T_2 for resonance II compared to I and III.

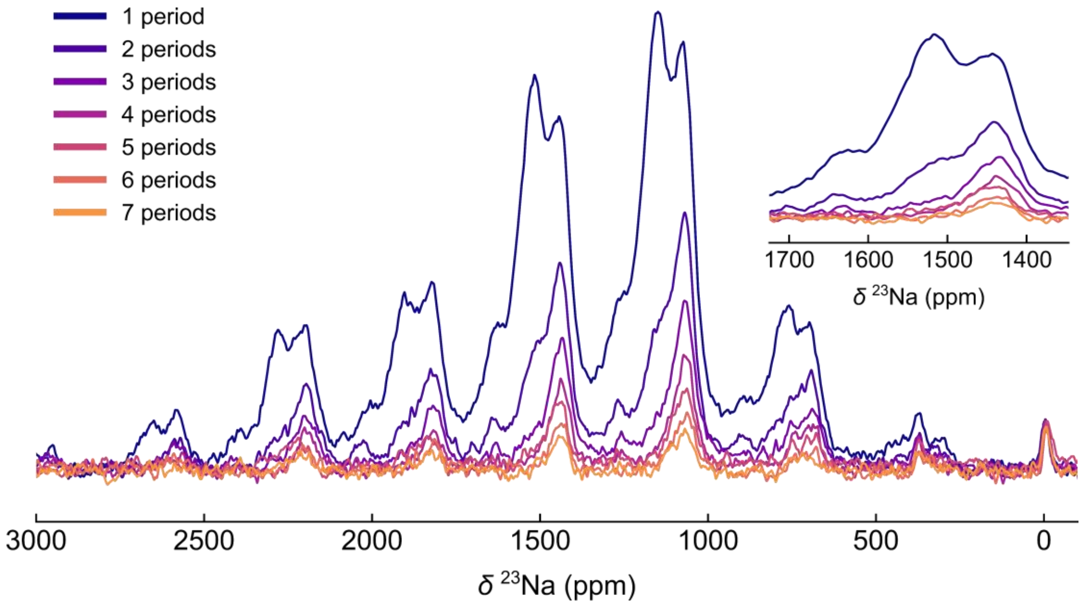

Figure S19: T_2-filtered Hahn-echo experiments; the echo delay (time between the 90° and 180° pulses) was varied and a spectrum recorded. Inset shows a zoom-in on the isotropic region of the spectrum. Spectra were recorded under 50 kHz MAS (sample temperature 305 K) and an applied field of 11.7 T.

Comparison of Hahn-echo spectra at different magnetic field strengths

To decide which magnetic field is most appropriate for variable-temperature NMR experiments, Hahn-echoes (under 60 kHz MAS speed) at different magnetic fields strengths were recorded [Figure S20]. The spectra acquired at 4.7 T has a lower resolution than at 11.7 T, presumably on account of the increased line broadening from the quadrupolar interaction at low fields. Whilst low fields limit the extent of signal broadening from paramagnetic relaxation enhancement, the effect of the quadrupolar interaction is exacerbated. As a compromise between these interactions, we selected primarily fields of an intermediate strength.

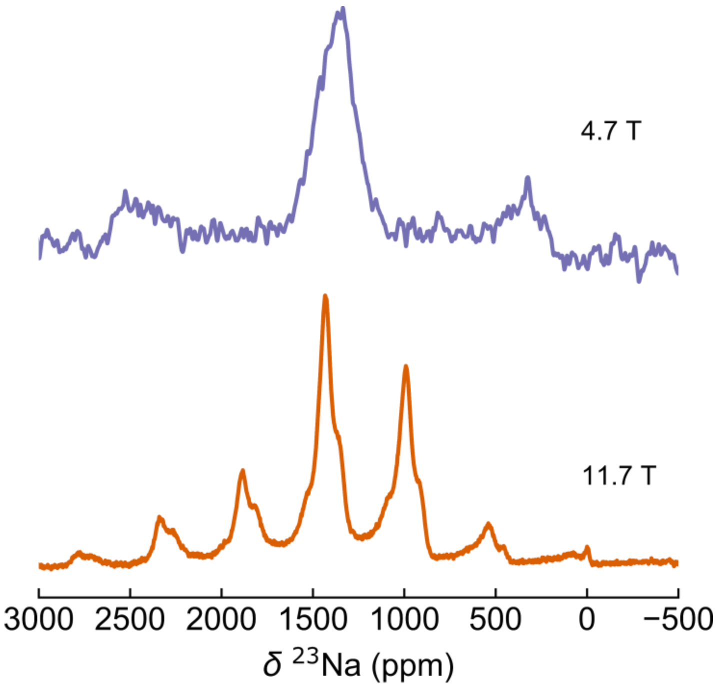

Figure S20: Hahn-echo spectra of NMMO acquired at a spinning speed of 60 kHz and under magnetic fields of 4.7 T and 11.7 T.

Curie-Weiss fits to experimental shifts as a function of temperature

The observed hyperfine shift, δ, may be related to the experimental sample temperature, T_s, using the Curie-Weiss law:

$$\delta = \frac{\alpha}{T - \theta},$$

where α is a quantity which is proportional the amount of unpaired electron spin density at the nuclear position and θ is the Weiss constant, which reports the mean magnetic exchange interaction field. The observed shifts for NMMO can be fit as a function of temperature; such fits reveal Weiss constants of: +0.220 K, 0.285 K and 0.294 K for resonances I, II and III, respectively.

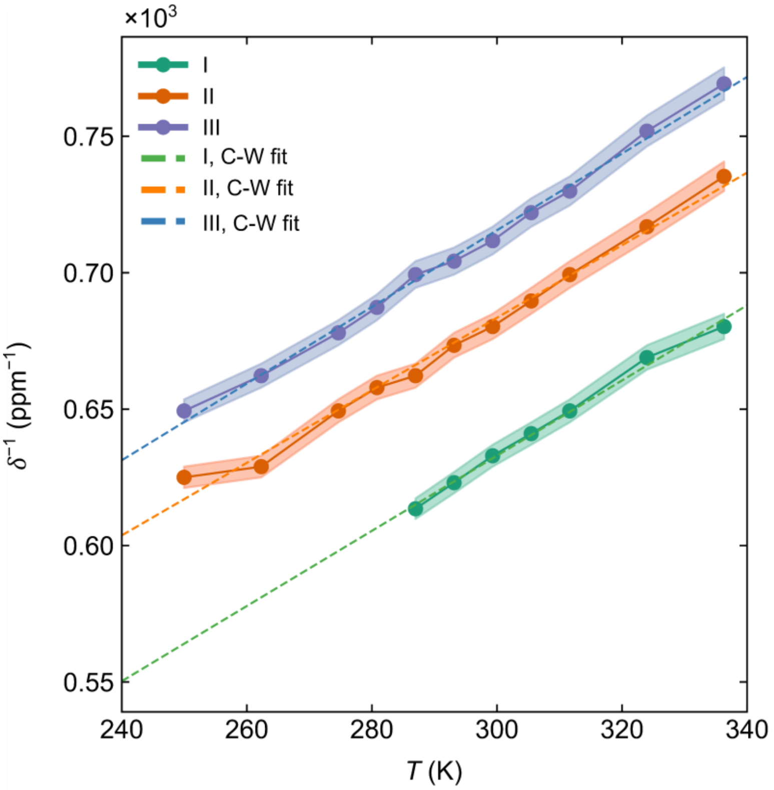

Figure S21: Observed ^{23}Na NMR shifts as a function of temperature for the data acquired at 16.4 T, with Curie-Weiss law fits to the inverse of the shift as a function of temperature. Shaded region represents the estimated error in the observed isotropic shift.

Variation in signal integrals as a function of temperature

To track the changes in individual signal intensity as a function of temperature, fits of the Hahn-echoes as a function of temperature were obtained for the data acquired at 16.4 T and a spinning speed of 50 kHz. The spectra were fit using a chemical shift anisotropy model, as this acts as a surrogate to the anisotropic hyperfine interaction. The quadrupolar interaction was omitted from the fit to avoid overfitting: it was found that adding the quadrupole to the fit made negligible improvement, and, since the quadrupolar interaction is effectively suppressed at high fields, we assume it makes little or no contribution on the appearance of these spectra.

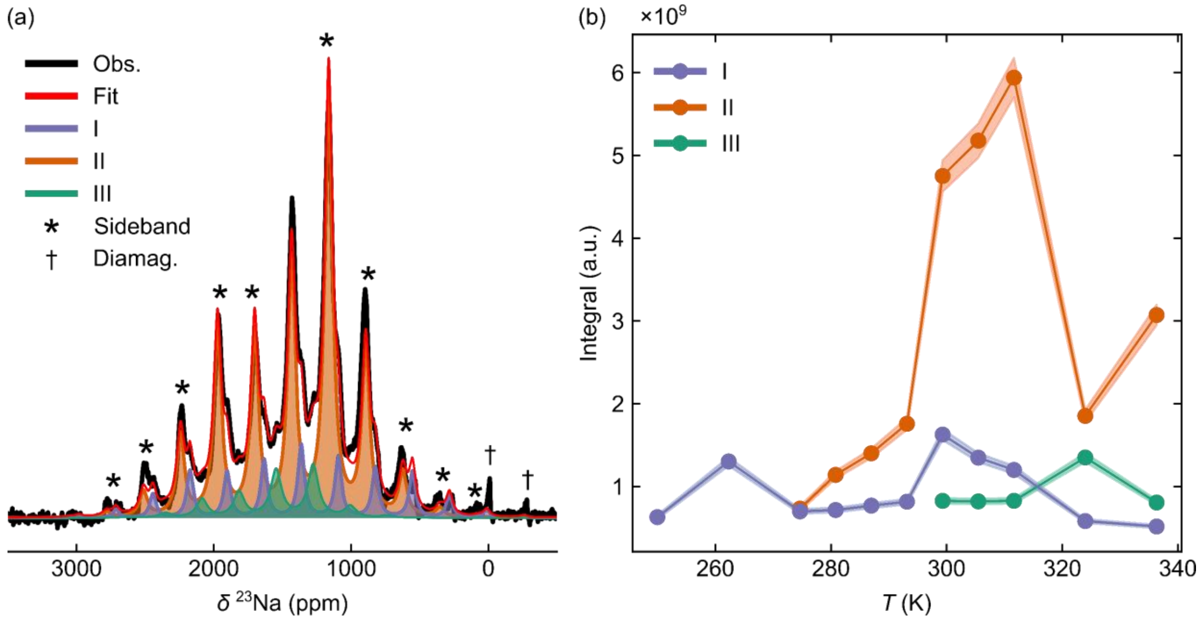

Figure S22: (a) example fit of the Hahn-echo spectrum of NMMO at 337 K (50 kHz, 16.4 T). In **(b)**, the temperature variation in T_2-weighted integral (isotropic and sidebands) of each component in the fit. Fill along the lines indicates the approximate error in the integral.

Low-Temperature ^{23}Na NMR Spectroscopy.

Even lower temperature ^{23}Na NMR spectroscopy was used to (attempt to) eliminate any Na^+ ion motion and further investigate cation ordering on the *TM* sublattice. On cooling NMMO to 242 K in a 4 mm rotor with MAS 12.5 kHz, at least two broad, overlapping lineshapes are observed, spanning from −1500 ppm to +4500 ppm, suggesting a wide distribution of overlapping resonances from different paramagnetic environments [Figure S23(a)]; these low-temperature spectra were acquired *via* variable offset cumulative spectroscopy (VOCS) to excite the full spectrum. Superimposed on these broad resonances is a set of sharp resonances centred on 0 ppm with a large overall width (span). These arise from nuclei in diamagnetic environments, the large span being a consequence of both the strong quadrupolar interaction, and large dipolar couplings to paramagnetic centres in nearby particles, and also because these diamagnetic environments are likely on the surface of the particles. Further cooling to 100 K (30 kHz, 9.4 T) results in a severely broadened resonance spanning −2000 ppm to +7000 ppm [Figure S23(b)]. Note that the sharp resonance at around

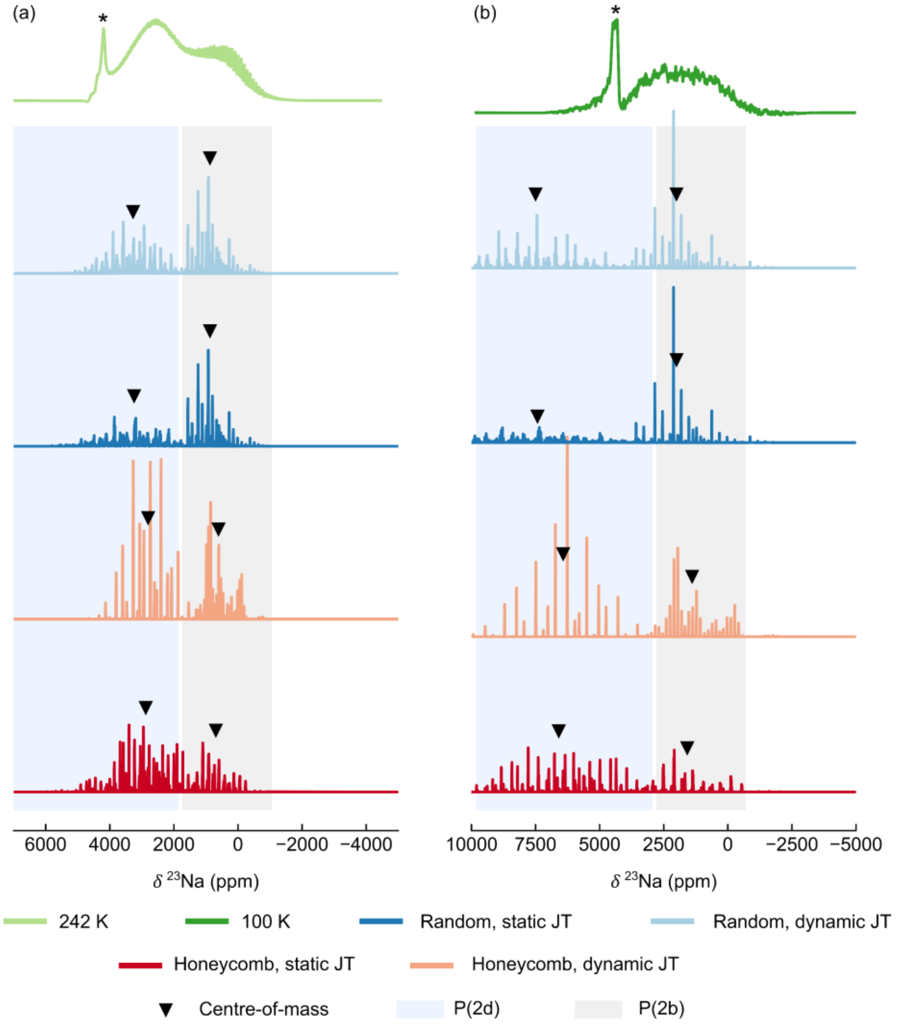

Figure S23: Low-temperature ^{23}Na NMR spectra for pristine NMMO and comparison to calculated shifts, which were obtained from bond pathways assuming a random distribution of Mg, Mn^{3+} and Mn^{4+} centres or a honeycomb distribution of Mg and Mn^{4+} cations. In **(a)**, the 242 K data at 12.5 kHz MAS and 11.7 T is shown, whilst **(b)** shows the 100 K data at 30 kHz MAS and 9.4 T; both spectra were obtained *via* variable offset cumulative spectroscopy. The asterisk denotes the resonance from Cu in the coil. Note that the simulated spectra contain isotropic resonances only. Arrows above the experimental spectra indicate the centre-of-mass shift for P(2d) and P(2b) sites, in each model. Note that the multiple peaks in the simulated spectra correspond to different local environments. We have not included the sidebands that would result from these multiple resonances as this would result in even more complex spectra.

4400 ppm is Cu from the NMR probe coil; this was seen in the spectra recorded at 11.7 T [Figure 5(a)] but omitted for the sake of clarity. The increase of the overall breadth of the NMMO resonances from 242 to 100 K likely stems from the increased dipolar couplings between the ^{23}Na ions and nearby paramagnets.

The no/slower hopping at low temperatures means that Na signals from all the different local environments will, in principle, be observed. In reality, we anticipate that, due to the larger time-averaged electron spin moment at lower temperatures, the T_1s and T_2s of each Na environment will be shortened substantially, resulting in broader, less intense lines (both isotropic and sideband manifold) than might be expected from the slow hopping regime [Figure S1]. Furthermore, the increased strength of the anisotropic hyperfine (dipolar) interaction will reduce the intensity of individual components in the sideband manifold. This, in combination with the slower spinning speeds, results in featureless lineshapes from which it is difficult to extract the number and positions of the isotropic resonances. Unfortunately, pjMATPASS experiments to isolate the isotropic resonances were unsuccessful.

The broad spectra were compared to simulated spectra of models in which: the Mg^{2+}, Mn^{3+} and Mn^{4+} are randomly distributed over the TMO_2 layer; and Mg^{2+} and $Mn^{3+/4+}$ are honeycomb-ordered in the TMO_2 layer [Figure S23]. As expected, the random model contains more resonances and extends over a slightly wider range of frequencies at both temperatures than the honeycomb ordered model. The random model comprises 9786 unique environments—7056 of which, or 72%, are P(2d), and 2730, or 28%, are P(2b)—while the honeycomb ordered model comprises 495 unique environments—225, or 45%, of which are P(2d) and 270, or 55%, are P(2b)—the change in proportions reflecting the greater number of unique combinations local honeycomb ordering can be achieved at the P(2b) site than P(2d). In agreement with our previous work,[20] the P(2d) sites dominate the high-frequency portion of the spectrum, while the P(2b) sites dominate the low-frequency portion.

We note that the centre-of-mass of the computed spectra are approximately: 2295 and 5260 ppm for the random *TM* distribution at 242 K and 100 K, respectively; or, for the honeycomb distribution, 2000 and 4590 ppm at 242 K and 100 K, respectively. In both cases, the centre-of-mass shifts are a better match in the honeycomb model than the random *TM* distribution model. In both *TM* distributions, there is negligible difference in the centre-of-mass shifts between the static and a dynamic JT distortion models.

The 242 K spectrum contains resonances in both the P(2d) and P(2b) regions, the majority of the intensity is in the P(2d) region, suggesting a small fraction of ions in the (thermodynamically less preferred) P(2b) sites and reflecting the honeycomb-ordered model proportions. Integration of the 242 K spectrum gives approximately 40% of signal in the P(2b) region and 60% in P(2d), though it is acknowledged that, without accurate T_2 measurements (challenging on such broad spectra) and presence of the superimposed diamagnetic peaks, these integrals are approximate.

We also note that the 242 K spectrum was recorded using an MAS rate of 12.5 kHz, which we expect to be insufficient to average the anisotropic hyperfine interactions, leading to the severe broadening. Comparing this to the spectrum at 250 K under 50 kHz MAS rate, we see the isotropic resonance at 250 K occurs at a similar shift to that at 242 K. Since the weighted average of the static shifts of the P(2d) and P(2b) environments straddle the isotropic resonance seen at 250 K, we expect both spectra exhibit exchange between the P(2d) and P(2b) sites.

At 100 K, however, signals are only seen in the P(2b) region of the spectrum. This is surprising given the expected thermodynamic preference of P(2d) sites. The match between experiment

and the honeycomb-ordered and random models is similar at 100 K: it is not possible to conclude ordering from this spectrum alone. The origin of the occupation of only the P(2b) region is unclear: one possibility is that P(2d) Na^{+} ions relax much faster than those in the P(2b) sites, resulting in a broader signal which is much harder to excite, leaving only the P(2b) environments observable.

Simulation of 11.7 T Variable-Temperature ^{23}Na NMR Spectra

To test the simulation model used in the main text, additional simulations using linearly interpolated and extrapolated hopping rates from the fits in Figure 7. on the variable temperature ^{23}Na NMR spectra at 11.7 T were carried out [Figure S24 and Figure S25]. As with the simulations of the 16.4 T data, the simulations reproduce the observed spectra fairly well. Small differences in the lineshapes and relative intensities can be ascribed to the effects of the quadrupolar and dipolar hyperfine interactions which were not accounted for in this

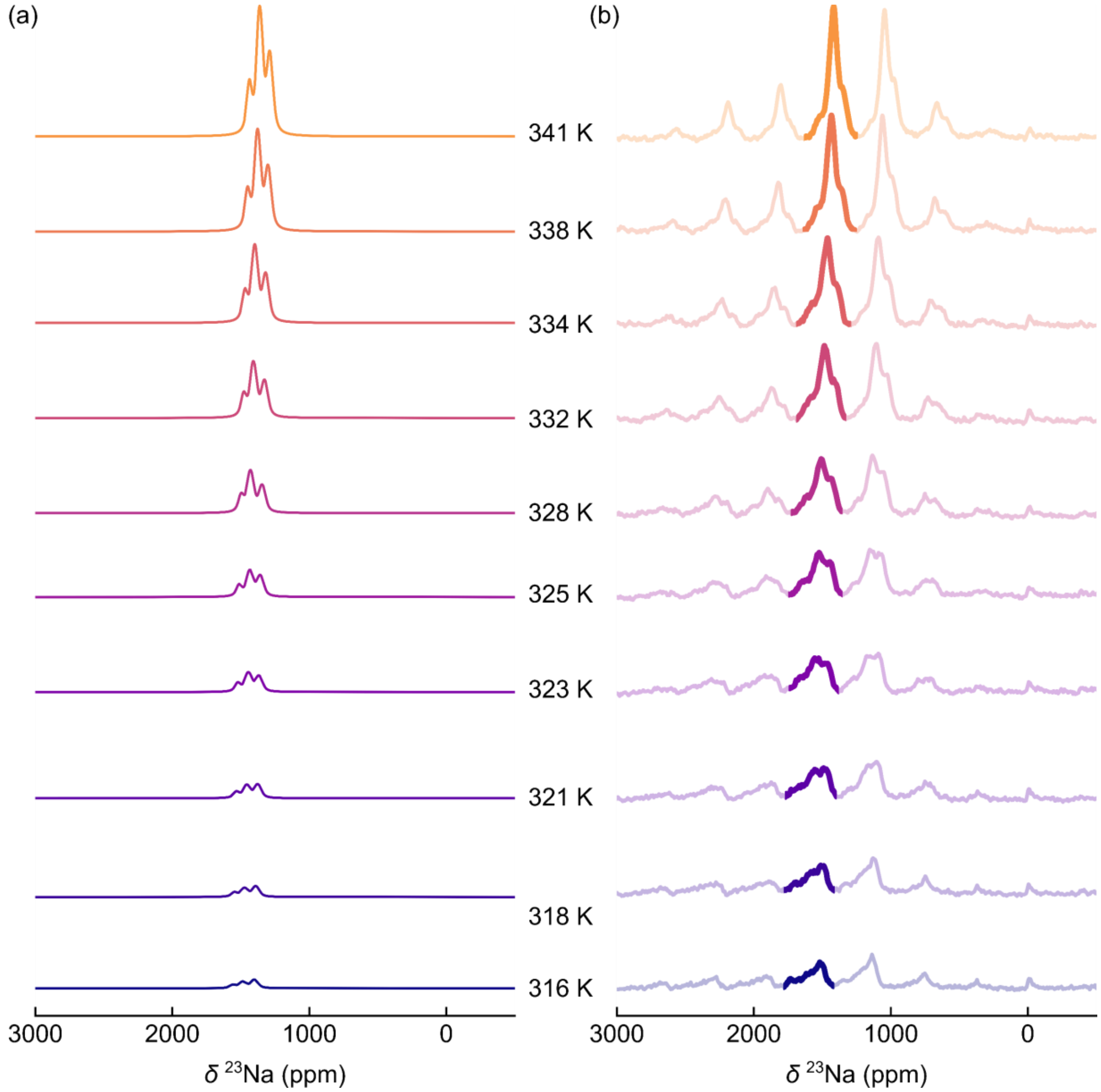

Figure S24: Comparison of **(a)** simulated and **(b)** observed variable-temperature ^{23}Na NMR spectra for NMMO recorded at 11.7 T under 50 kHz MAS rate. Isotropic resonances in **(b)** are highlighted in bold.

simulation model. The results nevertheless indicate the effect of Na^+ ion motion on the observed spectra.

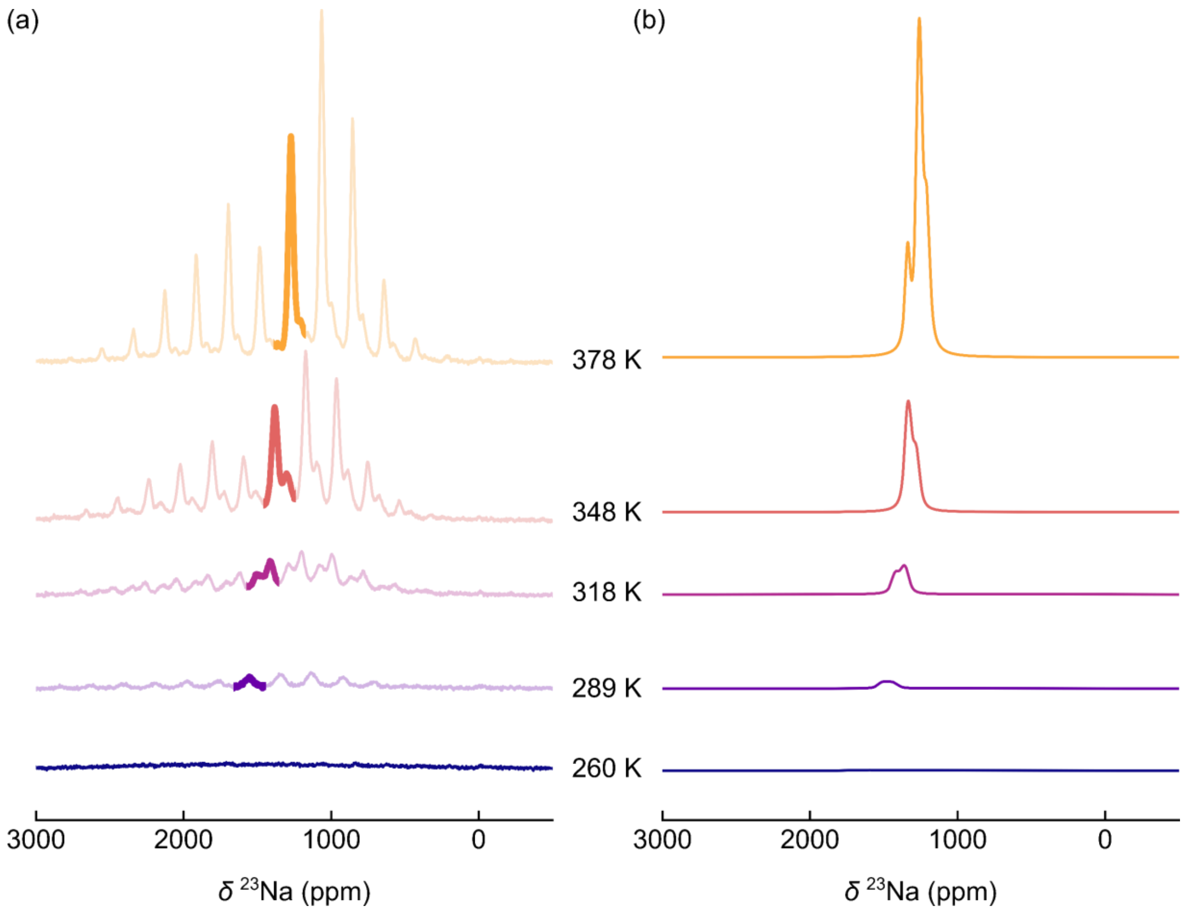

Figure S25: Comparison of **(a)** observed and **(b)** simulated variable-temperature ^{23}Na NMR spectra for NMMO recorded at 11.7 T under 28 kHz MAS rate. Isotropic resonances are highlighted in bold.

Arrhenius Fitting of Hopping Rates

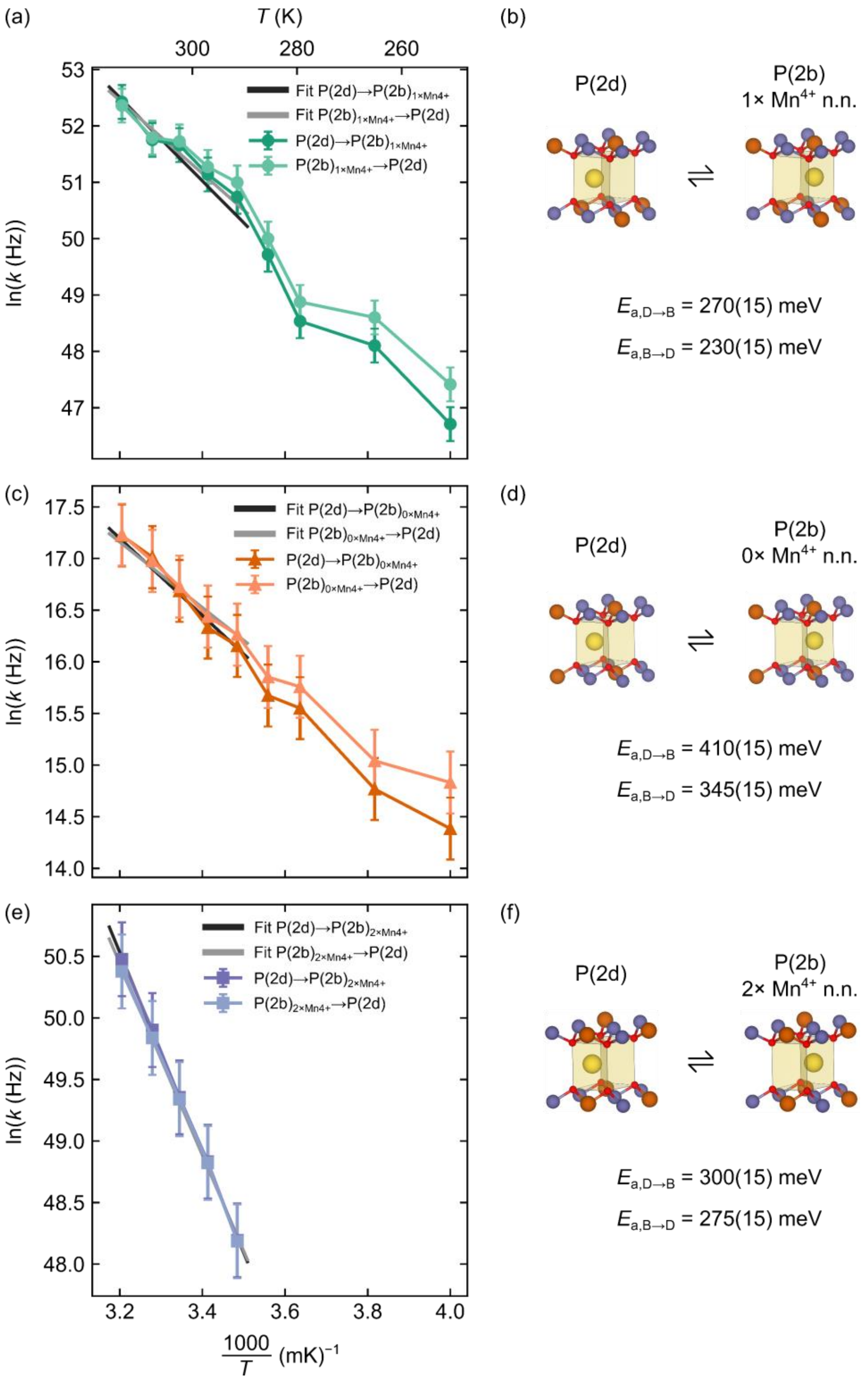

Figure S26: Arrhenius fit of the natural logarithm of the hopping rate, *k*, versus 1000/*T*, to obtain activation energy barriers for Na^+ ion hopping between: **(a)** and **(b)** a P(2d) site and a P(2b) site with one Mn^{4+} and one Mg^{2+} nearest neighbour; **(c)** and **(d)** a P(2d) site and a P(2b) site with two Mn^{4+} nearest neighbours and **(e)** and **(f)** a P(2d) site and a P(2b) site with two Mg^{2+} nearest neighbours.

The variation in the natural logarithm of the hopping rate constants, *k*, from the variable-temperature ^{23}Na NMR simulations were fit to an Arrhenius equation, from which the barriers to hopping were extracted [Figure S26]. These barriers are only approximate, as the model does not recreate the intensities perfectly; the error on each of these barriers is estimated at 15 meV.

For all three types of hop considered, the hop from a P(2d) to a P(2b) site is greater than the hop from P(2b) to P(2d), which can be assigned to the higher energy of P(2b) sites compared to P(2d), due to greater Coulombic repulsion in P(2b). Notably, the barriers for hops between P(2d) and a P(2b) with no Mn^{4+} nearest neighbours is the largest, which can be ascribed to the large Coulomb repulsion at these P(2b) sites compared to P(2b) sites with fewer Mn^{4+} next nearest neighbours. The barriers for hopping between P(2d) and P(2b) with either one or two Mn^{4+} nearest neighbours are very similar in size, which can be attributed to the similar amounts of Coulomb repulsion in each of these sites: whilst the P(2b) with one Mn^{4+} nearest neighbour has more Mn^{4+} nearest neighbours, it has fewer Mn^{4+} next nearest neighbours, such that the total repulsion experienced at these two P(2b) sites are similar.

The effect of Mn^{3+} on simulations

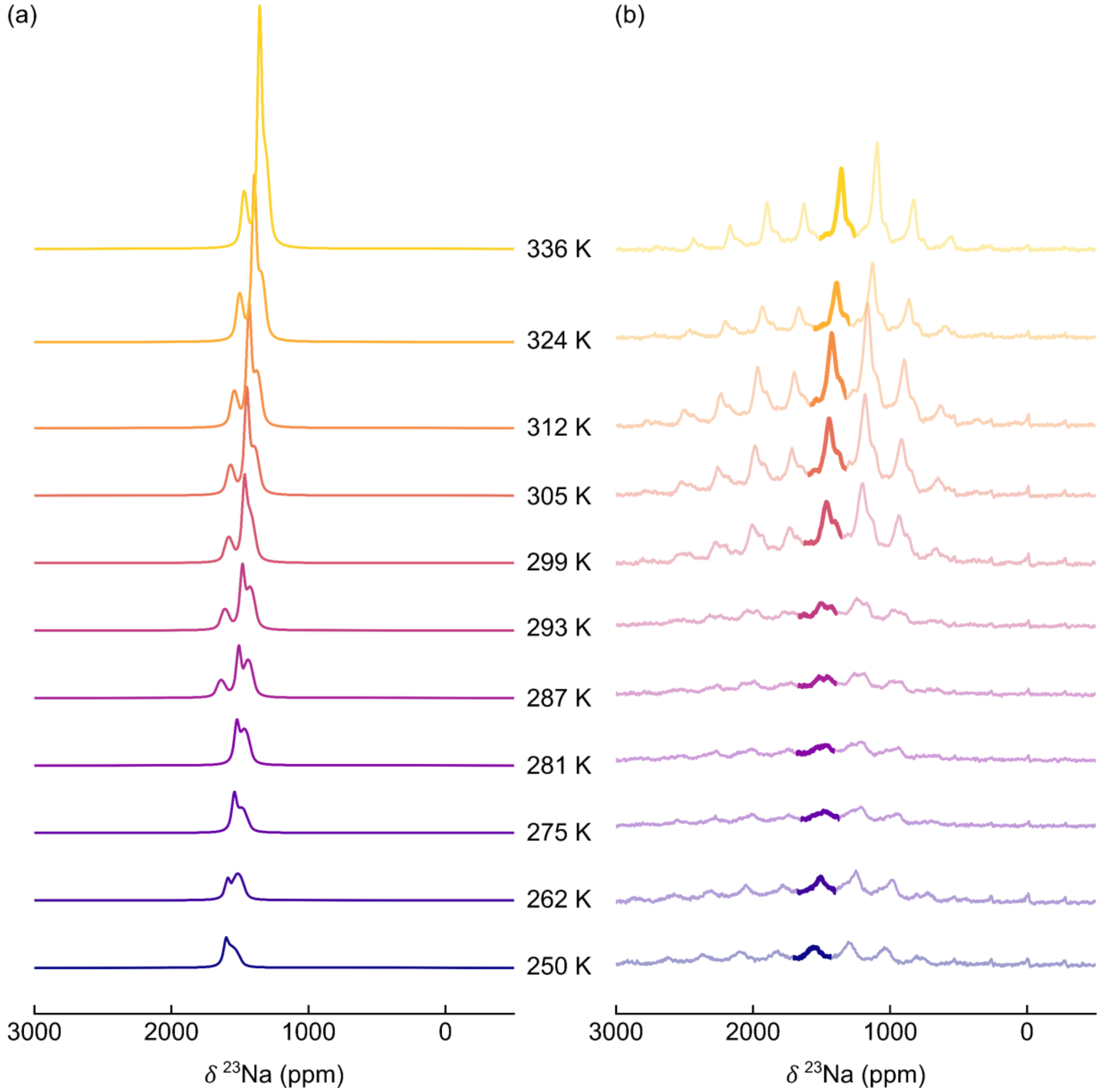

Figure S27: Comparison of **(a)** simulated and **(b)** observed variable-temperature ^{23}Na NMR spectra for NMMO recorded at 16.4 T under 50 kHz MAS rate. Isotropic resonances in **(b)** are highlighted in bold. The simulations include Mn^{3+} bond pathways for the central resonance exchanging sites.

To investigate the effect of Na^{+} sites with Mn^{3+} nearest neighbours, we performed Bloch-McConnell simulations [Figure S27]. The best match to the experimental data obtained corresponded to Na^{+} two of the same types of hops seen in the main text—honeycomb-ordered P(2d) Na^{+} hopping to a honeycomb-ordered P(2b) sites with one or two Mn^{4+} ions in the nearest neighbour coordination shell. The third site is also from a honeycomb-ordered P(2d) site with only Mn^{4+} and Mg^{2+} as nearest and next-nearest neighbours, but goes to a honeycomb P(2b) with one Mn^{3+} next-nearest neighbour. This corresponded to the highest probability Mn^{3+}-containing honeycomb-ordered environment [Table S5]. The energy barriers obtained from an Arrhenius fit to the data gives values between 39 meV and 320 meV [Figure S28]. While the spectra look qualitatively similar, we suggest this fit is poorer than that presented in the main text, especially when the Arrhenius fits are examined. The variation in

the hopping frequency as a function of temperature suggests mass transport occurs, with rate constants which differ significantly in the forward and reverse directions. The size of the barriers also seem much lower than might be reasonably expected.

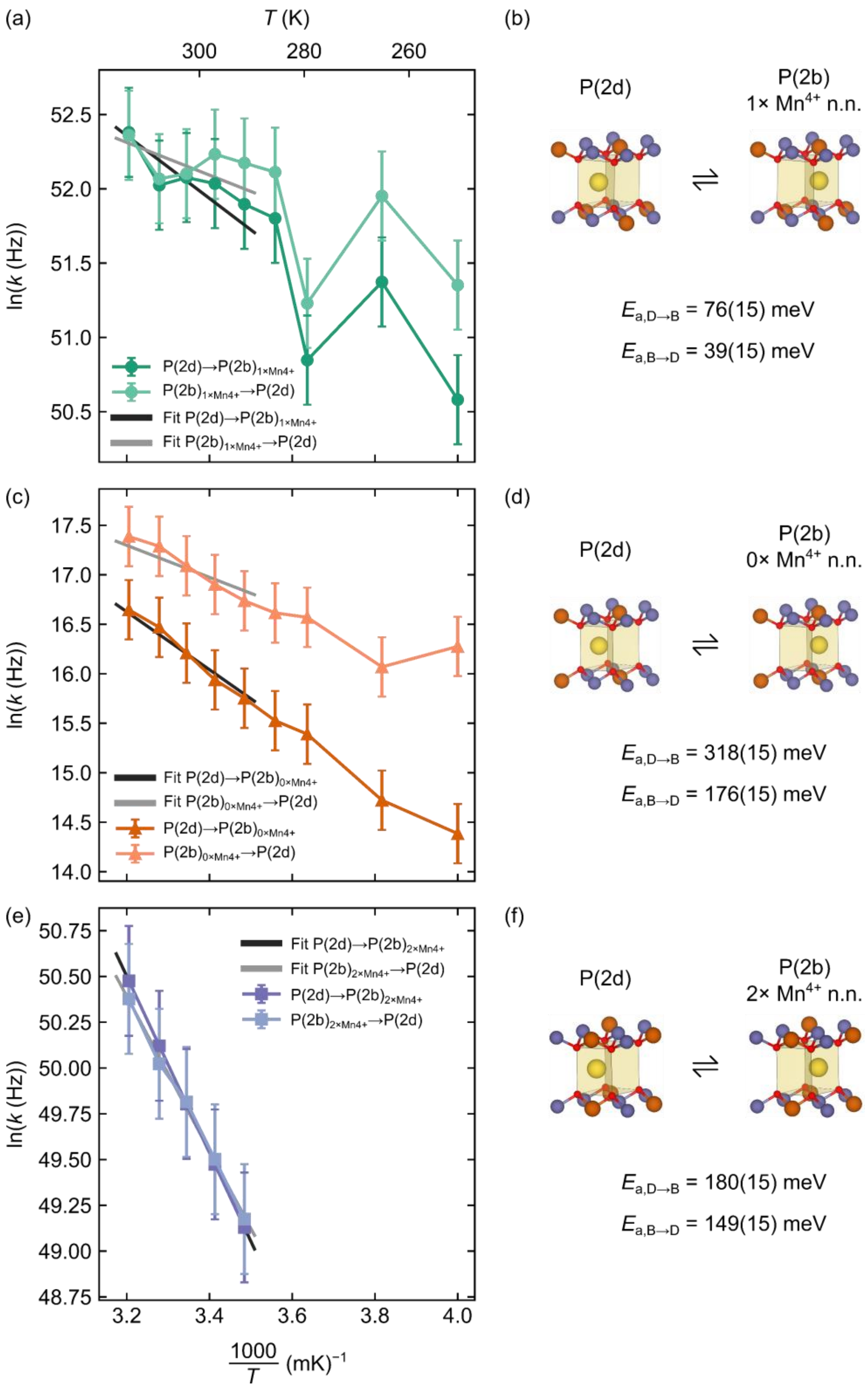

Figure S28: Arrhenius fit of the natural logarithm of the hopping rate, *k*, versus 1000/*T*, to obtain activation energy barriers for Na^{+} ion hopping between: **(a)** and **(b)** a P(2d) site and a P(2b) site with one Mn^{4+} and one Mg^{2+} nearest neighbour; **(c)** and **(d)** a P(2d) site and a P(2b) site with two Mn^{4+} nearest neighbours and **(e)** and **(f)** a P(2d) site and a P(2b) site with two Mg^{2+} nearest neighbours.

S8. Bond pathways in NMMO, assigning the NMR spectrum and differences between models used

In our previous work, we assigned the ^{23}Na NMR spectrum to Na^+ ions hopping between sites in the Na layers of NMMO but were unable to identify the environments between which Na^+ was hopping.[25] Such an assignment was based on qualitative observations of the variable-temperature ^{23}Na NMR spectra collected and on the basis that the shifts of sites computed from bond pathways did not match to the observed spectrum unless mixing (hopping) between sites was allowed. Our previous work only used Mn^{4+}, bond pathways, however. In the current work, we have successfully computed the Mn^{3+} bond pathways for the two types of crystallographic Na site, P(2d) and P(2b), by adding additional Na^+ sites into our previous model to create $Na_{1/2}[Mg_{1/6}Mn^{3+}{}_{1/6}Mn^{4+}{}_{2/3}]O_2$, which was as close as possible to the true stoichiometry while preserving the P2 structure. We note that, due to the Jahn-Teller distortion of Mn^{3+}, the Mn^{3+}–O–Na^+ pathway may proceed *via* a lengthened (L) or shortened (S) Mn^{3+}–O bond. In the cases where Mn^{3+} interacts *via* a 90°-like pathway (i.e., Mn^{3+} is a nearest neighbour, as for Mn_A and Mn_C in Figure S29(a) and (b), respectively).

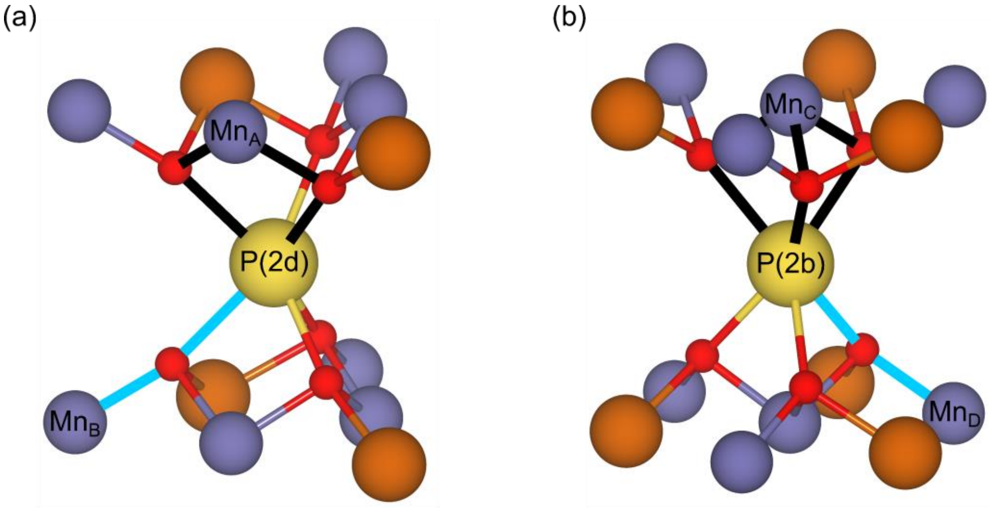

	Hyb35	Hyb20
Mn^{4+}_{A}	+459 ppm	+542 ppm
Mn^{4+}_{B}	−12 ppm	−15 ppm
$Mn^{3+}_{A,SS}$	+224 ppm	+498 ppm
$Mn^{3+}_{A,SL}$	−83 ppm	+117 ppm
$Mn^{3+}_{B,S}$	+98 ppm	+298 ppm
$Mn^{3+}_{B,L}$	+656 ppm	+929 ppm

	Hyb35	Hyb20
Mn^{4+}_{C}	−268 ppm	−222 ppm
Mn^{4+}_{D}	+95 ppm	+104 ppm
$Mn^{3+}_{C,2S+L}$	−825 ppm	−665 ppm
$Mn^{3+}_{D,S}$	−11 ppm	+221 ppm
$Mn^{3+}_{D,L}$	+116 ppm	+367 ppm

Figure S29: Computed ^{23}Na NMR hyperfine (Fermi contact) bond pathways for Mn^{3+} and Mn^{4+} ions in the nearest or next-nearest coordination shell of Na^+ in **(a)** the P(2d) sites and **(b)** the P(2b) sites. Note that Mn^{3+} ions are Jahn-Teller distorted, so pathways may involve individual or combinations of long (L) and short (S) Mn^{3+}–O pathways. Shifts shown are calculated at 318 K, corresponding to a 60 kHz MAS speed. Model used was $Na_{1/2}[Mg_{1/6}Mn^{3+}{}_{1/6}Mn^{4+}{}_{2/3}]O_2$ ($a \approx 10.20$ Å, $b \approx 5.10$ Å, $c \approx 22.86$ Å).

In an analogous manner to our previous paper, we can compute the expected shifts for P(2d) and P(2b) environments assuming either a "random" distribution of Mg, Mn^{3+} and Mn^{4+}, or honeycomb order between Mg and $Mn^{3+/4+}$. Briefly, the total Fermi contact shift of the Na nucleus, δ_{FC}, is given by summing the Fermi contact bond pathways, $\delta_{\mathrm{path},i}$, over both the nearest and next-nearest coordination shells of transition metal (*TM*) ions relative to Na^+:[2,3]

$$\delta_{\mathrm{FC}} = \sum_i z_i \delta_{\mathrm{path},i}, \tag{12}$$

where z_i is the number of such bond pathways around Na. On changing the experimental temperature, the shift must be re-scaled from the zero-Kelvin isotropic hyperfine coupling constant, A_{iso}, using the Curie-Weiss law using the scaling parameter Φ:

$$\delta_{\mathrm{FC}} = \frac{A_{\mathrm{iso}}}{\omega_0 h}\Phi, \tag{13}$$

where h is Planck's constant and ω_0 is the Larmor frequency of the observed nucleus. The scaling parameter Φ is given by:

$$\Phi = \frac{B_0 \mu_{\mathrm{eff}}^2}{3 k_{\mathrm{B}} g_{\mathrm{e}} \mu_{\mathrm{B}} (T-\theta)}, \tag{14}$$

where B_0 is the applied magnetic field strength, μ_{eff} is the effective magnetic moment, k_{B} is the Boltzmann constant, g_{e} the free electron *g*-factor, μ_{B} the Bohr magneton, T the experimental temperature and θ the Weiss constant. In addition, one must consider the quadrupole-induced shift—a change in the Zeeman-split states when the quadrupolar interaction is "large" (approximately 10% or more of the Zeeman splitting[26]). This is given by:

$$\delta_{\mathrm{QIS}} = -\frac{\nu_Q^2}{30\nu_0^2}\left[I(I+1)-\frac{3}{4}\right]\left(1+\frac{\eta_Q^2}{3}\right), \tag{15}$$

where ν_0 is the nuclear Larmor frequency (in Hz), *I* is the nuclear spin quantum number, η_Q is the asymmetry of the quadrupolar interaction () and ν_Q the quadrupolar frequency (in Hz), defined as:

$$\nu_Q = \frac{3C_Q}{2I(2I-1)}. \tag{16}$$

Here, C_Q is the quadrupolar coupling constant:

$$C_Q = \frac{e^2 q Q}{\hbar}, \tag{17}$$

which relates the anisotropy of the local electric field gradient tensor, *eq*. The quadrupole-induced shift is then added to the total Fermi contact shift, $\delta_{\mathrm{tot}} = \delta_{\mathrm{FC}} + \delta_{\mathrm{QIS}}$, to give the total shift of the nucleus. The computed quadrupolar coupling constants and shifts are given in Table S1.

Table S1: *Ab initio* calculated quadrupolar coupling constants, quadrupolar asymmetries and quadrupole-induced shifts for $Na_{1/2}[Mg_{1/6}Mn^{3+}_{1/6}Mn^{4+}_{2/3}]O_2$ ($a \approx 10.20$ Å, $b \approx 5.10$ Å, $c \approx 22.86$ Å), using hybrid 35 and hybrid 20 functionals computed assuming an NMR field of 11.7 T. Note that these parameters are averages across all sites (P(2d) and P(2b)), as there was essentially no difference between the values of C_Q, η_Q and δ_{QIS} for the two sites. Note that environments with only Mn^{4+} and with one Mn^{3+} nearby (either as first- or second-nearest neighbours) have different C_Qs and quadrupole-induced shifts, but that the P(2d) and P(2b) environments show almost identical quadrupolar parameters.

		Hyb35	Hyb20
	C_Q (MHz)	3.21	3.25
Mn^{4+}-only	η_Q	0.051	0.047
	δ_{QIS} (ppm)	−9.5	−9.7
	C_Q (MHz)	3.60	3.40
One Mn^{3+} nearby	η_Q	0.18	0.19
	δ_{QIS} (ppm)	−9.3	−9.2

The quadrupolar shift, therefore, makes a negligible contribution to the observed shift of ^{23}Na in NMMO.

Computing the spectra under static models

The total number of possible combinations of *TM* ions in the first, N_1, and second, N_2, coordination shells can be obtained from the binomial coefficients to determine the number of combinations with allowed repeats by selecting r items from n possibilities:

$$N = C(n + r - 1, r) = \frac{(n + r - 1)!}{r!\,(n - 1)!}.$$

Here, for the P(2d) sites, $r_1 = r_2 = 6$ (i.e., the first and second coordination shell each have six possible *TM* sites which can be occupied) and for the P(2b) sites, $r_1 = 2$ and $r_2 = 12$.

The number of possible *TM* interactions are: $n_1 = n_2 = 4$ for the P(2d) sites—noting that an interaction of Mn^{3+} in the first shell could be *via* a short and long Mn^{3+}–O bond, or *via* two short bonds (as Mn^{3+} is Jahn-Teller distorted), or for the second shell *via* either a short or a long bond. For P(2b) sites, $n_1 = 3$ (there is only one unique way in which Mn^{3+} can coordinate to Na in the P(2b) site, as there will always be two short and one long bond) and $n_2 = 4$ (as Mn^{3+} can now interact either *via* a shortened or lengthened Mn^{3+}–O bond. This gives $N_{1,P(2d)} = N_{2,P(2d)} = 84$, so a total of $N_{P(2d)} = N_{1,P(2d)}N_{2,P(2d)} = 7056$ unique environments for P(2d), and $N_{1,P(2b)} = 6$ and $N_{2,P(2b)} = 455$, so a total of $N_{P(2b)} = N_{1,P(2b)}N_{2,P(2b)} = 2730$ unique environments for P(2b).

If it is assumed the Mn^{3+} Jahn-Teller is dynamic, however, we obtain $N_{1,P(2d)} = N_{2,P(2d)} = 28$ and a total of $N_{P(2d)} = 784$ unique environments for P(2d) sites. For P(2b) sites, $N_{1,P(2b)} = 6$ and $N_{2,P(2b)} = 91$, giving $N_{P(2b)} = 546$ unique environments. Note that, under a dynamic Jahn-Teller, the bond pathways from Mn^{3+} are averaged:

$$\delta_{\mathrm{P(2d),n.n.}} = \frac{\delta_{\mathrm{SL}} + 2\delta_{\mathrm{SS}}}{3},$$

$$\delta_{\mathrm{P(2d),n.n.n.}} = \frac{\delta_{\mathrm{L}} + 2\delta_{\mathrm{S}}}{3},$$

$$\delta_{\mathrm{P(2d),n.n.n.}} = \frac{\delta_{\mathrm{L}} + 2\delta_{\mathrm{S}}}{3},$$

where δ_{SL}, δ_{SS}, δ_L and δ_S are the bond pathways with: nearest-neighbour Mn^{3+} connected to Na^+ *via* one short and one long Mn^{3+}–O bond; nearest-neighbour Mn^{3+} connected *via* two short Mn^{3+}–O bonds; next-nearest neighbour Mn^{3+} connected *via* a long Mn^{3+}–O bond and next-nearest neighbour Mn^{3+} connected *via* a short Mn^{3+}–O bond, respectively.

The probability of each environment, *P*, is the product of concentrations of the *TM*s at the first and second nearest neighbour sites, which corresponds to the composition: p = 0.61 for $Mn^{4+}$and p = 0.28 for Mg^{2+}. For Mn^{3+}, we must separate probabilities for interactions *via* short and long bonds: for example, if a nearest neighbour Mn^{3+} is connected to Na^+ *via* one short bond (local probability 2/3) and one long bond (local probability 1/3), the probability is (4/12)×0.11, (4/12) is the probability of the connection to Na^+ occurring *via* two short bonds (4 out of 12 pairs are short-short) and 0.11 the total Mn^{3+} concentration. These probabilities are listed in Table S2. Therefore, the total probabilities, *P*, for each coordination shell of the P(2d) and P(2b) environments under a static Jahn-Teller are:

$$P_{\mathrm{P(2d)},1} = n_1(\mathrm{Mn}^{4+}) \cdot p_1(\mathrm{Mn}^{4+}) \cdot n_1(\mathrm{Mg}^{2+}) \cdot p_1(\mathrm{Mg}^{2+}) \cdot n_1(\mathrm{Mn}_{\mathrm{SS}}^{3+}) \cdot p_1(\mathrm{Mn}_{\mathrm{SS}}^{3+}) \cdot n_1(\mathrm{Mn}_{\mathrm{SL}}^{3+}) \cdot p_1(\mathrm{Mn}_{\mathrm{SL}}^{3+})$$

$$P_{\mathrm{P(2d)},2} = n_2(\mathrm{Mn}^{4+}) \cdot p_2(\mathrm{Mn}^{4+}) \cdot n_2(\mathrm{Mg}^{2+}) \cdot p_2(\mathrm{Mg}^{2+}) \cdot n_2(\mathrm{Mn}_{\mathrm{S}}^{3+}) \cdot p_2(\mathrm{Mn}_{\mathrm{S}}^{3+}) \cdot n_2(\mathrm{Mn}_{\mathrm{L}}^{3+}) \cdot p_2(\mathrm{Mn}_{\mathrm{L}}^{3+})$$

$$P_{\mathrm{P(2b)},1} = n_1(\mathrm{Mn}^{4+}) \cdot p_1(\mathrm{Mn}^{4+}) \cdot n_1(\mathrm{Mg}^{2+}) \cdot p_1(\mathrm{Mg}^{2+}) \cdot n_1(\mathrm{Mn}^{3+}) \cdot p_1(\mathrm{Mn}^{3+})$$

$$P_{\mathrm{P(2d)},2} = n_2(\mathrm{Mn}^{4+}) \cdot p_2(\mathrm{Mn}^{4+}) \cdot n_2(\mathrm{Mg}^{2+}) \cdot p_2(\mathrm{Mg}^{2+}) \cdot n_2(\mathrm{Mn}_{\mathrm{S}}^{3+}) \cdot p_2(\mathrm{Mn}_{\mathrm{S}}^{3+}) \cdot n_2(\mathrm{Mn}_{\mathrm{L}}^{3+}) \cdot p_2(\mathrm{Mn}_{\mathrm{L}}^{3+})$$

Clearly in the presence of a dynamic Jahn-Teller, the probabilities simplify to:

$$P_1 = n_1(\mathrm{Mn}^{4+}) \cdot p_1(\mathrm{Mn}^{4+}) \cdot n_1(\mathrm{Mg}^{2+}) \cdot p_1(\mathrm{Mg}^{2+}) \cdot n_1(\mathrm{Mn}^{3+}) \cdot p_1(\mathrm{Mn}^{3+})$$

$$P_2 = n_2(\mathrm{Mn}^{4+}) \cdot p_2(\mathrm{Mn}^{4+}) \cdot n_2(\mathrm{Mg}^{2+}) \cdot p_2(\mathrm{Mg}^{2+}) \cdot n_2(\mathrm{Mn}^{3+}) \cdot p_2(\mathrm{Mn}^{3+})$$

for both the P(2d) and P(2b) environments.

The total probability of an environment is then given by the product of the probabilities of the two coordination shells, P_1P_2, and the probability of occupying a P(2d) or a P(2b) site, P_{site} (0.6 and 0.4, respectively, based on the crystal symmetry). In other words:

$$P = P_1 P_2 P_{\mathrm{site}}$$

These probabilities are then used to scale the lines for each corresponding unique environment.

Table S2: Probabilities of Mn^{4+}, Mn^{3+} and Mg^{2+} at the nearest neighbour (p_1) and next-nearest neighbour (p_2) sites to Na^+ in NMMO, rounded to four decimal places, computed for the random distribution model.

Species and connection to Na^+	p_1	p_2
Mn^{4+}	0.6100	0.6100
Mg^{2+}	0.2800	0.2800
Mn^{3+}_{SS}	0.0366	n/a
Mn^{3+}_{SL}	0.0733	n/a
Mn^{3+}_{S}	n/a	0.0733
Mn^{3+}_{L}	n/a	0.0366

In the case of honeycomb ordering, we must artificially restrict the distribution of Mg^{2+}, Mn^{3+} and Mn^{4+}. The possible combinations are given in Table S3 and Table S4: for P(2d) sites, there must always be four $Mn^{3+/4+}$ nearest and next-nearest neighbours, and two Mg^{2+} nearest and next-nearest neighbours. For P(2b) sites, there can be zero, one or two Mg^{2+}, $Mn^{3+/4+}$ nearest neighbours; the occupation of this site dictates the ordering of the surrounding, next-nearest neighbour sites: for Mn as a nearest neighbour in one layer (above or below Na), there must be six Mg and six Mn next-nearest neighbours, whilst for Mg as a nearest neighbour, there must be six Mn as the next-nearest neighbours.

Table S3: Probabilities and distributions of Mn^{4+}, Mn^{3+} and Mg^{2+} at the nearest and next-nearest neighbour P(2d) sites to Na^+ in NMMO, rounded to four decimal places, computed for the honeycomb-ordered distribution model. Note that, for Mn^{3+} as a nearest- or next-nearest neighbour, there are two distinct coordinations relative to Na^+, depending on whether long or short Mn^{3+}–O bonds coordinate to Na. The four highest probability environments are highlighted in blue.

	No. of nearest neighbours			No. of next-nearest neighbours			
Site	Mn^{4+}	Mg^{2+}	Mn^{3+}	Mn^{4+}	Mg^{2+}	Mn^{3+}	Probability
P(2d)	4	2	0	4	2	0	6.721E-01
P(2d)	4	2	0	3	2	1	1.212E-01
P(2d)	4	2	0	2	2	2	2.186E-02
P(2d)	4	2	0	1	2	3	3.941E-03
P(2d)	4	2	0	0	2	4	7.107E-04
P(2d)	3	2	1	4	2	0	1.212E-01
P(2d)	3	2	1	3	2	1	2.186E-02
P(2d)	3	2	1	2	2	2	3.941E-03
P(2d)	3	2	1	1	2	3	7.107E-04
P(2d)	3	2	1	0	2	4	1.282E-04
P(2d)	2	2	2	4	2	0	2.186E-02
P(2d)	2	2	2	3	2	1	3.941E-03
P(2d)	2	2	2	2	2	2	7.107E-04
P(2d)	2	2	2	1	2	3	1.282E-04
P(2d)	2	2	2	0	2	4	2.311E-05
P(2d)	1	2	3	4	2	0	3.941E-03
P(2d)	1	2	3	3	2	1	7.107E-04
P(2d)	1	2	3	2	2	2	1.282E-04
P(2d)	1	2	3	1	2	3	2.311E-05
P(2d)	1	2	3	0	2	4	4.168E-06
P(2d)	0	2	4	4	2	0	7.107E-04
P(2d)	0	2	4	3	2	1	1.282E-04
P(2d)	0	2	4	2	2	2	2.311E-05
P(2d)	0	2	4	1	2	3	4.168E-06
P(2d)	0	2	4	0	2	4	7.515E-07

The most likely honeycomb-ordered environments are highlighted in Table S3 and Table S4.

Comparing the experimental pjMATPASS spectrum of NMMO to the simulated static spectra, in which Na^+ motion is assumed to be frozen and Na^+ occupy either P(2d) or P(2b) sites only (i.e., no Na^+ sit in between these sites), we observe a poor fit to both models [Figure S30 and Figure S31]. In both the random and honeycomb ordered models, the P(2d) sites dominate the region of the observed spectrum, as seen in our earlier work. The experimentally observed region straddles the P(2d) and P(2b) static sets of Na sites, further indicating that Na^+ ion motion is necessary to describe the observed spectra.

Table S4: Probabilities and distributions of Mn^{4+}, Mn^{3+} and Mg^{2+} at the nearest and next-nearest neighbour P(2b) sites to Na^{+} in NMMO, rounded to four decimal places, computed for the honeycomb-ordered distribution model. Note that, for Mn^{3+} as a next-nearest neighbour, there are two distinct coordinations relative to Na^{+}, depending on whether long or short Mn^{3+}–O bonds coordinate to Na. The four highest probability environments are highlighted in blue.

	No. of nearest neighbours			No. of next-nearest neighbours			
Site	Mn^{4+}	Mg^{2+}	Mn^{3+}	Mn^{4+}	Mg^{2+}	Mn^{3+}	Probability
P(2b)	2	0	0	6	6	0	2.79E-02
P(2b)	2	0	0	5	6	1	5.04E-03
P(2b)	2	0	0	4	6	2	9.08E-04
P(2b)	2	0	0	3	6	3	1.64E-04
P(2b)	2	0	0	2	6	4	2.95E-05
P(2b)	2	0	0	1	6	5	5.33E-06
P(2b)	2	0	0	0	6	6	9.61E-07
P(2b)	1	0	1	6	6	0	5.04E-03
P(2b)	1	0	1	5	6	1	9.08E-04
P(2b)	1	0	1	4	6	2	1.64E-04
P(2b)	1	0	1	3	6	3	2.95E-05
P(2b)	1	0	1	2	6	4	5.33E-06
P(2b)	1	0	1	1	6	5	9.61E-07
P(2b)	1	0	1	0	6	6	1.73E-07
P(2b)	0	0	2	6	6	0	9.08E-04
P(2b)	0	0	2	5	6	1	1.64E-04
P(2b)	0	0	2	4	6	2	2.95E-05
P(2b)	0	0	2	3	6	3	5.33E-06
P(2b)	0	0	2	2	6	4	9.61E-07
P(2b)	0	0	2	1	6	5	1.73E-07
P(2b)	0	0	2	0	6	6	3.12E-08
P(2b)	1	1	0	8	3	1	2.39E-02
P(2b)	1	1	0	7	3	2	4.31E-03
P(2b)	1	1	0	6	3	3	7.77E-04
P(2b)	1	1	0	5	3	4	1.40E-04
P(2b)	1	1	0	4	3	5	2.53E-05
P(2b)	1	1	0	3	3	6	4.56E-06
P(2b)	1	1	0	2	3	7	8.22E-07
P(2b)	1	1	0	1	3	8	1.48E-07
P(2b)	1	1	0	0	3	9	2.67E-08
P(2b)	0	1	1	9	3	0	2.39E-02
P(2b)	0	1	1	8	3	1	4.31E-03
P(2b)	0	1	1	7	3	2	7.77E-04
P(2b)	0	1	1	6	3	3	1.40E-04
P(2b)	0	1	1	5	3	4	2.53E-05
P(2b)	0	1	1	4	3	5	4.56E-06
P(2b)	0	1	1	3	3	6	8.22E-07
P(2b)	0	1	1	2	3	7	1.48E-07
P(2b)	0	1	1	1	3	8	2.67E-08
P(2b)	0	1	1	0	3	9	4.82E-09
P(2b)	0	2	0	12	0	0	6.29E-01
P(2b)	0	2	0	11	0	1	1.13E-01
P(2b)	0	2	0	10	0	2	2.05E-02
P(2b)	0	2	0	9	0	3	3.69E-03
P(2b)	0	2	0	8	0	4	6.65E-04
P(2b)	0	2	0	7	0	5	1.20E-04
P(2b)	0	2	0	6	0	6	2.16E-05
P(2b)	0	2	0	5	0	7	3.90E-06
P(2b)	0	2	0	4	0	8	7.04E-07
P(2b)	0	2	0	3	0	9	1.27E-07
P(2b)	0	2	0	2	0	10	2.29E-08
P(2b)	0	2	0	1	0	11	4.13E-09
P(2b)	0	2	0	0	0	12	7.44E-10

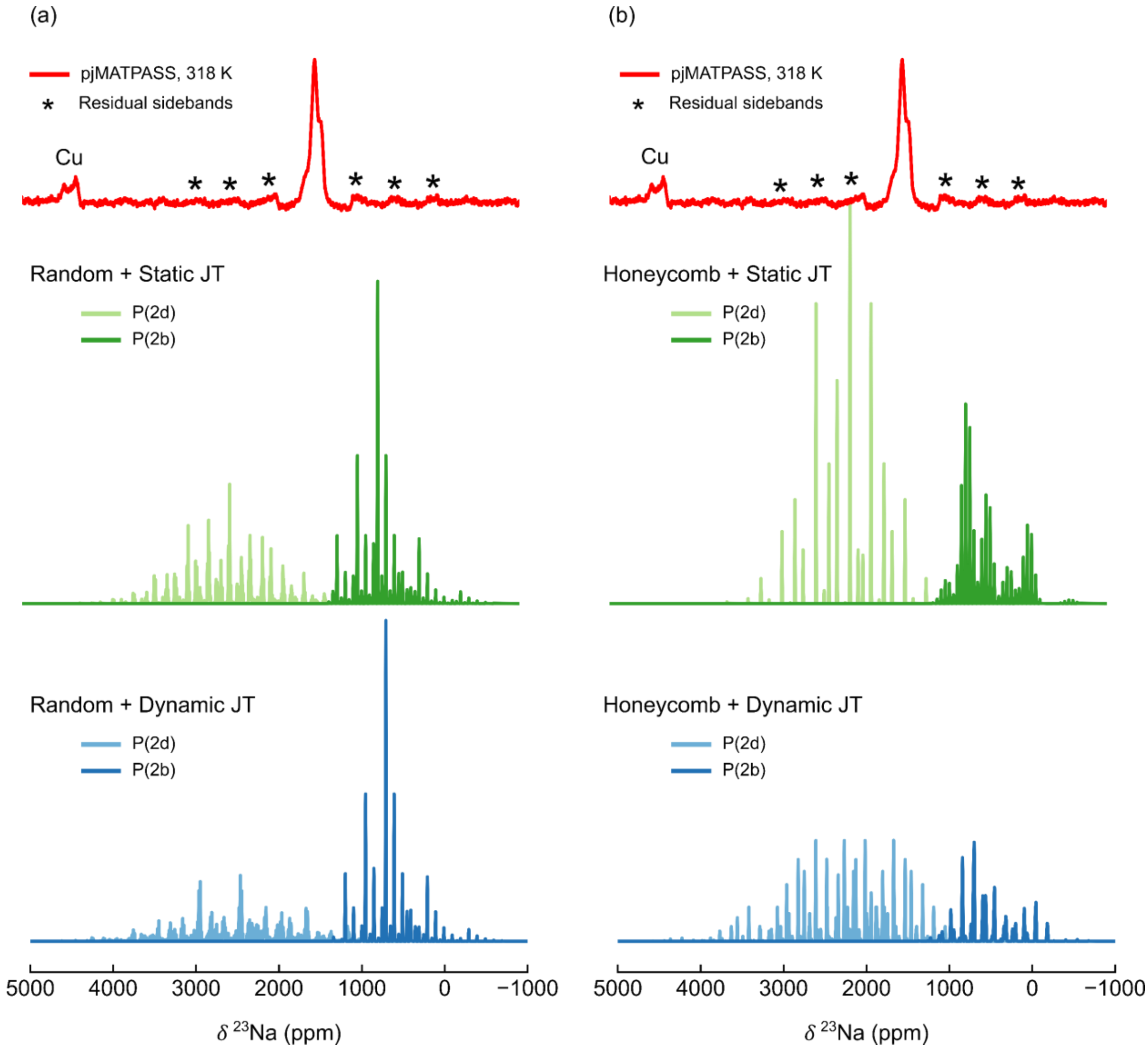

Figure S30: Comparison between the projected magic angle turning phase adjusted sideband suppression (pjMATPASS) spectrum recorded at 11.7 T and 318 K (60 kHz MAS speed) for NMMO, and bond pathway-based models. In **(a)** and **(b)**, the random and honeycomb-ordered models are compared to the observed spectrum, with a static (middle) and dynamic (bottom) Jahn-Teller (JT) distortion at Mn^{3+}.

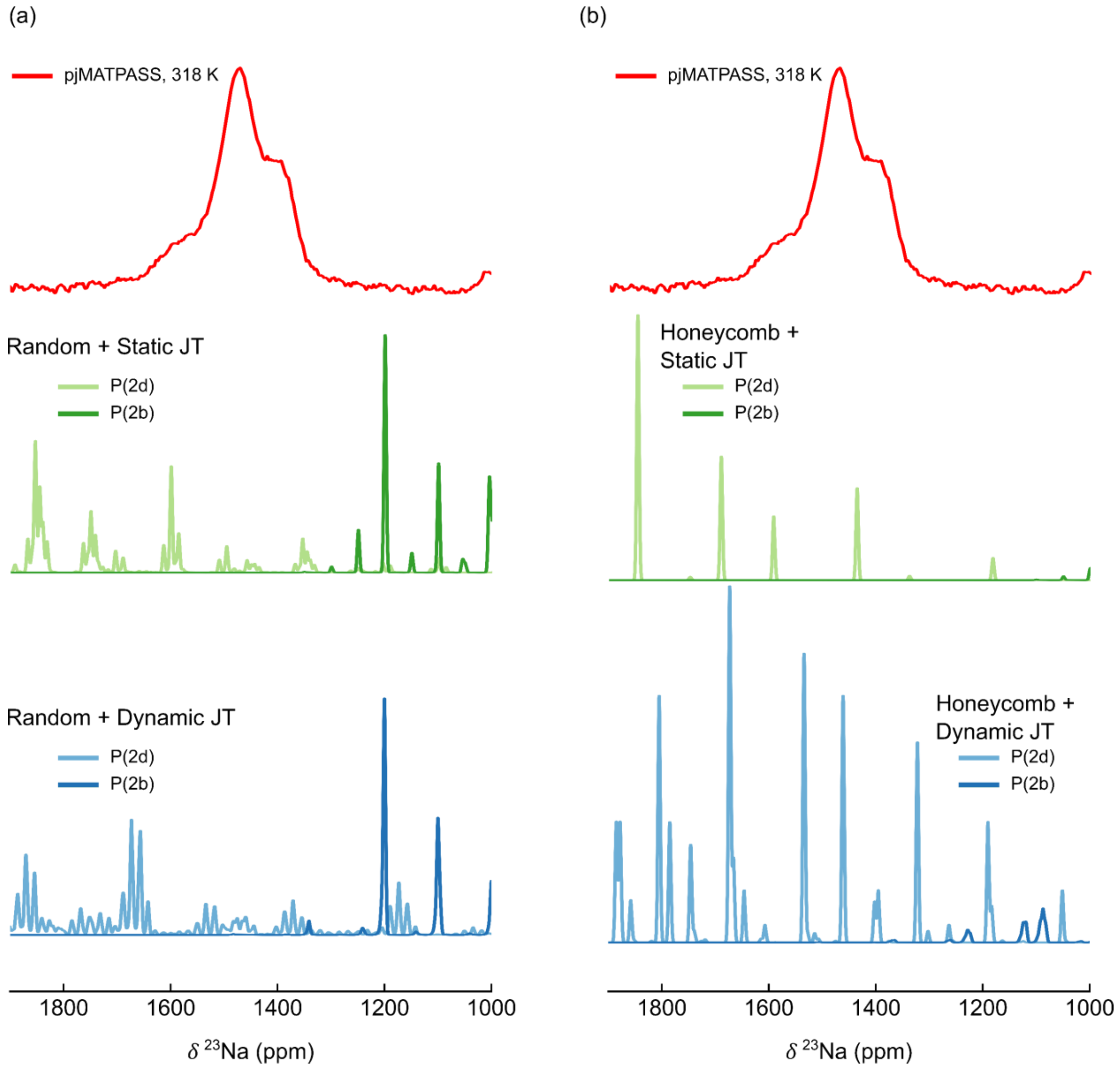

Figure S31: Comparison between the projected magic angle turning phase adjusted sideband suppression (pjMATPASS) spectrum recorded at 11.7 T and 318 K (60 kHz MAS speed) for NMMO, and bond pathway-based models, zoomed in on the experimentally observed region. In **(a)** and **(b)**, the random and honeycomb-ordered models are compared to the observed spectrum, with a static (middle) and dynamic (bottom) Jahn-Teller (JT) distortion at Mn^{3+}.

Rationale for using the simplified Bloch-McConnell simulations to model spectra

The ^{23}Na NMR spectra for NMMO are clearly affected by motion, with at least some of the Na^+ sites entering the intermediate regime of motion at low temperatures (see Figure 5 of main text; between 250 to 275 K, only one broad signal can be seen clearly). As such, we simulated our spectra using the Bloch-McConnell model outlined in section S2. The model presented in the main text, however, only uses Mn^{4+}-based bond pathways to generate the shifts of the sites, rather than the Mn^{3+} environments. The reason for this is threefold: firstly, the error bar on the Mn^{3+} bond pathways is much larger, making it harder to predict the shifts accurately. Secondly, the Mn^{4+}-based environments are among the most likely, according to the honeycomb model and random models used above. Finally, as the material adopts a mostly ordered structure in which Mn^{4+} and Mg^{2+} almost exclusively occupy only one set of *TM* sites each (i.e. the material is almost fully honeycomb-ordered), such a material would have the formula $Na_{2/3}[Mg_{1/3}Mn_{2/3}]O_2$ and therefore will only contain Mn^{4+} centres. The Mn^{3+} will therefore make a much smaller contribution.

We also note that modelling the Jahn-Teller distortion in NMMO is challenging: in particular, it is unclear whether the Jahn-Teller is dynamic or static. It is anticipated it will be dynamic, at most of the experimentally observed NMR temperatures (except perhaps the 100 K data). As it is unclear how the timescale of the JT changes as a function of temperature and how this influences the ^{23}Na NMR spectrum, we concluded it would be simplest to focus on Mn^{4+}-only-environments.

It should also be noted that, examining the probabilities of occupying the honeycomb sites and the products of these probabilities—a proxy for the probability of a hop—the Mn^{4+}/Mg^{2+}-only sites account for approximately 60% of all site exchange.

Table S5: Highest probability Na sites with Mn^{4+}, Mn^{3+} and Mg^{2+} at the nearest and next-nearest neighbour P(2d) and P(2b) sites to Na^+ in NMMO, computed for the honeycomb-ordered distribution model. The products of these site probabilities is also shown, alongside the proportion of the overall site exchanges (i.e., the probability product divided by the sum of all probability products).

Site	Arbitrary label	No. of nearest neighbours			No. of next-nearest neighbours			Probability
		Mn^{4+}	Mg^{2+}	Mn^{3+}	Mn^{4+}	Mg^{2+}	Mn^{3+}	
P(2b)	b_1	0	2	0	12	0	0	6.29E-01
P(2b)	b_2	1	1	0	9	3	0	1.33E-01
P(2b)	b_3	0	2	0	11	0	1	1.13E-01
P(2b)	b_4	2	0	0	6	6	0	2.79E-02
P(2d)	d_1	4	2	0	4	2	0	6.721E-01
P(2d)	d_2	4	2	0	3	2	1	1.212E-01
P(2d)	d_3	3	2	1	4	2	0	1.212E-01
P(2d)	d_4	4	2	0	2	2	2	2.186E-02

Probability products	d_1	d_2	d_3	d_4
b_1	0.423	0.076	0.076	0.014
b_2	0.089	0.016	0.016	0.003
b_3	0.076	0.014	0.014	0.002
b_4	0.019	0.003	0.003	0.001

Probability proportions	d_1	d_2	d_3	d_4
b_1	50.0%	9.0%	1.6%	1.6%
b_2	10.5%	1.9%	0.3%	0.3%
b_3	9.0%	1.6%	0.3%	0.3%
b_4	2.2%	0.4%	0.1%	0.1%

S9. Additional *ab initio* calculations of Na^+ hopping pathways

Climbing Image Nudged Elastic Band Results

It was hoped that either single- or double-ended transition state searches might be used to obtain the energy barriers to Na^+ ion hopping in NMMO. Unfortunately, these proved incredibly challenging to obtain, which is ascribed to a relatively shallow potential energy surface, making convergence of the transition state challenging. To provide approximate values of the barriers and assess to what extent Mg^{2+} doping influences the size of Na^+ ion hopping barriers, climbing-image nudged elastic band (CI-NEB) calculations on $Na_{1/2}MnO_2$ and $Na_{1/2}[Mg_{1/6}Mn_{5/6}]O_2$ were carried out [Figure S32]. The energy profiles indicate that both the P(2b) and P(2d) sites are local energy minima. Note that, at 80%, the Na^+ is still essentially in the P(2b) site, but is slightly offset from sitting directly above or below the Mg/Mn centre; it only displaces to sit exactly on top of the Mn site at 100% (arrow in Figure S32(a)). Bearing this in mind, the energy barrier to hopping in $Na_{1/2}MnO_2$ is approximately 280 meV; for $Na_{1/2}[Mg_{1/6}Mn_{5/6}]O_2$, the hop from P(2d) to P(2b) with one Mg^{2+} nearest neighbour and one Mn^{4+} nearest neighbour is approximately 180 meV. The second pathway in $Na_{1/2}[Mg_{1/6}Mn_{5/6}]O_2$ is more unusual; in all cases, calculations appeared to show Na^+ suddenly moving from the P(2d) to the P(2b) site, with no intermediate steps. Additional calculations are required to understand this behaviour, but it perhaps illustrates the shallow potential energy landscape when Mg^{2+} is present in the *TM* layer.

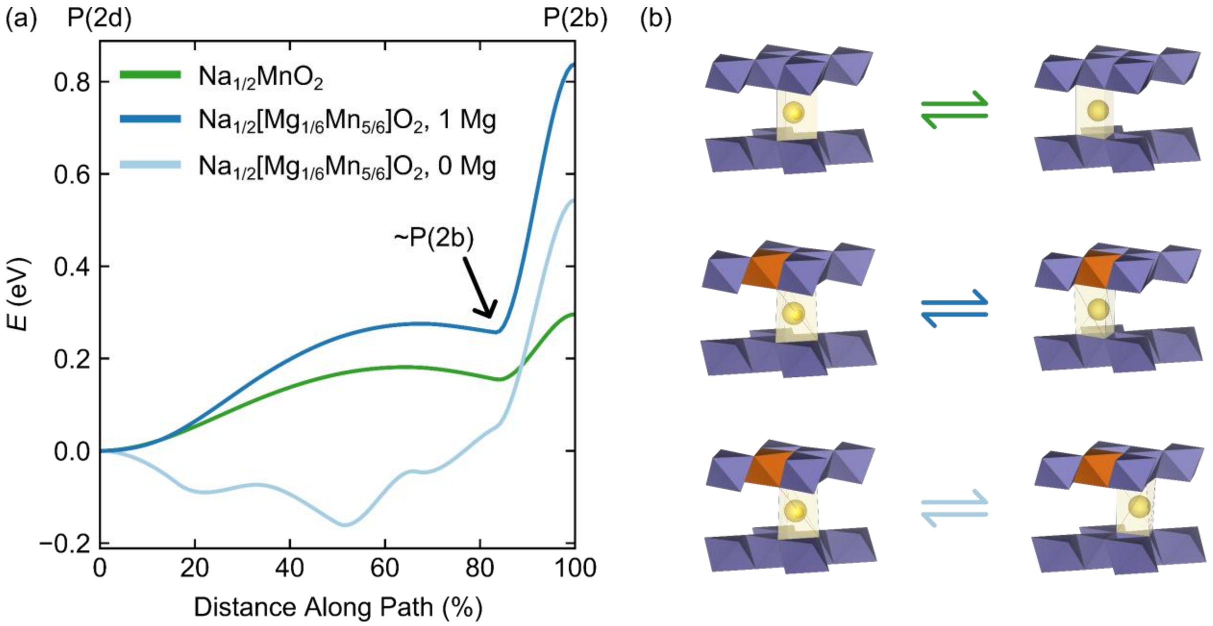

Figure S32: Climbing-image NEB results. In **(a)**, the energy profiles for Na^+ ion hops in $Na_{1/2}MnO_2$ and $Na_{1/2}[Mg_{1/6}Mn_{5/6}]O_2$ are shown, with 0% corresponding to the P(2d) site and 100% the P(2b) site. The arrow indicates that, at approximately 80% along the path, Na^+ is already in a P(2b) site; the remainder of the path involves moving directly on top of Mg/Mn. In **(b)**, the local environments of Na^+ at the beginning and end of the hop are shown.

References:

(1) Yabuuchi, N.; Hara, R.; Kubota, K.; Paulsen, J.; Kumakura, S.; Komaba, S. A New Electrode Material for Rechargeable Sodium Batteries: P2-Type Na2/3[Mg0.28Mn0.72]O2 with Anomalously High Reversible Capacity. *J Mater Chem A Mater* **2014**, *2* (40), 16851–16855. https://doi.org/10.1039/c4ta04351k.

(2) Maitra, U.; House, R. A.; Somerville, J. W.; Tapia-Ruiz, N.; Lozano, J. G.; Guerrini, N.; Hao, R.; Luo, K.; Jin, L.; Pérez-Osorio, M. A.; Massel, F.; Pickup, D. M.; Ramos, S.; Lu, X.; McNally, D. E.; Chadwick, A. v.; Giustino, F.; Schmitt, T.; Duda, L. C.; Roberts, M. R.; Bruce, P. G. Oxygen Redox Chemistry without Excess Alkali-Metal Ions in Na2/3[Mg0.28Mn0.72]O2. *Nat Chem* **2018**, *10* (3), 288–295. https://doi.org/10.1038/nchem.2923.

(3) Tartoni, N.; Thompson, S. P.; Tang, C. C.; Willis, B. L.; Derbyshire, G. E.; Wright, A. G.; Jaye, S. C.; Homer, J. M.; Pizzey, J. D.; Bell, A. M. T.; IUCr. High-Performance X-Ray Detectors for the New Powder Diffraction Beamline I11 at Diamond. *J Synchrotron Radiat* **2008**, *15* (1), 43–49. https://doi.org/10.1107/S0909049507046250.

(4) Thompson, S. P.; Parker, J. E.; Potter, J.; Hill, T. P.; Birt, A.; Cobb, T. M.; Yuan, F.; Tang, C. C. Beamline I11 at Diamond: A New Instrument for High Resolution Powder Diffraction. *Review of Scientific Instruments* **2009**, *80* (7), 075107. https://doi.org/10.1063/1.3167217.

(5) Day, P.; Enderby, J.; Williams, W.; Chapon, L.; Hannon, A.; Radaelli, P.; Soper, A. Scientific Reviews: GEM: The General Materials Diffractometer at ISIS-Multibank Capabilities for Studying Crystalline and Disordered Materials. *Neutron News* **2004**, *15* (1), 19–23. https://doi.org/10.1080/00323910490970564.

(6) Arnold, O.; Bilheux, J. C.; Borreguero, J. M.; Buts, A.; Campbell, S. I.; Chapon, L.; Doucet, M.; Draper, N.; Ferraz Leal, R.; Gigg, M. A.; Lynch, V. E.; Markvardsen, A.; Mikkelson, D. J.; Mikkelson, R. L.; Miller, R.; Palmen, K.; Parker, P.; Passos, G.; Perring, T. G.; Peterson, P. F.; Ren, S.; Reuter, M. A.; Savici, A. T.; Taylor, J. W.; Taylor, R. J.; Tolchenov, R.; Zhou, W.; Zikovsky, J. Mantid—Data Analysis and Visualization Package for Neutron Scattering and μ SR Experiments. *Nucl Instrum Methods Phys Res A* **2014**, *764*, 156–166. https://doi.org/10.1016/J.NIMA.2014.07.029.

(7) Coelho, A. A. *TOPAS* and *TOPAS-Academic* : An Optimization Program Integrating Computer Algebra and Crystallographic Objects Written in C++. *J Appl Crystallogr* **2018**, *51* (1), 210–218. https://doi.org/10.1107/S1600576718000183.

(8) Wasylishen,R.; Ashbrook, S.; Wimperis, S. Vega, A. J., Quadrupolar Nuclei in Solids. In *NMR of Quadrupolar Nuclei in Solid Materials*; Wiley, 2012; pp 17–44.

(9) Stoll, S.; Schweiger, A. EasySpin, a Comprehensive Software Package for Spectral Simulation and Analysis in EPR. *Journal of Magnetic Resonance* **2006**, *178* (1), 42–55. https://doi.org/10.1016/J.JMR.2005.08.013.

(10) Middlemiss, D. S.; Ilott, A. J.; Clément, R. J.; Strobridge, F. C.; Grey, C. P. Density Functional Theory-Based Bond Pathway Decompositions of Hyperfine Shifts: Equipping Solid-State NMR to Characterize Atomic Environments in Paramagnetic Materials. *Chemistry of Materials* **2013**, *25* (9), 1723–1734. https://doi.org/10.1021/cm400201t.

(11) Clément, R. J.; Xu, J.; Middlemiss, D. S.; Alvarado, J.; Ma, C.; Meng, Y. S.; Grey, C. P. Direct Evidence for High Na + Mobility and High Voltage Structural Processes in P2-Na x [Li y Ni z Mn 1−y−z]O 2 (x, y, z ≤ 1) Cathodes from Solid-State NMR and DFT Calculations. *J Mater Chem A Mater* **2017**, *5* (8), 4129–4143. https://doi.org/10.1039/C6TA09601H.

(12) Clément, R. J.; Pell, A. J.; Middlemiss, D. S.; Strobridge, F. C.; Miller, J. K.; Whittingham, M. S.; Emsley, L.; Grey, C. P.; Pintacuda, G. Spin-Transfer Pathways in Paramagnetic Lithium Transition-Metal Phosphates from Combined Broadband Isotropic Solid-State MAS NMR

Spectroscopy and DFT Calculations. *J Am Chem Soc* **2012**, *134* (41), 17178–17185. https://doi.org/10.1021/ja306876u.

(13) Kim, J.; Middlemiss, D. S.; Chernova, N. A.; Zhu, B. Y. X.; Masquelier, C.; Grey, C. P. Linking Local Environments and Hyperfine Shifts: A Combined Experimental and Theoretical 31 P and 7 Li Solid-State NMR Study of Paramagnetic Fe(III) Phosphates. *J Am Chem Soc* **2010**, *132* (47), 16825–16840. https://doi.org/10.1021/ja102678r.

(14) Kim, J.; Ilott, A. J.; Middlemiss, D. S.; Chernova, N. A.; Pinney, N.; Morgan, D.; Grey, C. P. 2H and 27Al Solid-State NMR Study of the Local Environments in Al-Doped 2-Line Ferrihydrite, Goethite, and Lepidocrocite. *Chemistry of Materials* **2015**, *27* (11), 3966–3978. https://doi.org/10.1021/acs.chemmater.5b00856.

(15) Kresse, G.; Hafner, J. Ab Initio Molecular Dynamics for Liquid Metals. *Phys Rev B* **1993**, *47* (1), 558–561. https://doi.org/10.1103/PhysRevB.47.558.

(16) Kresse, G.; Hafner, J. Ab Initio Molecular-Dynamics Simulation of the Liquid-Metalamorphous-Semiconductor Transition in Germanium. *Phys Rev B* **1994**, *49* (20), 14251–14269. https://doi.org/10.1103/PhysRevB.49.14251.

(17) Kresse, G.; Furthmüller, J. Efficiency of Ab-Initio Total Energy Calculations for Metals and Semiconductors Using a Plane-Wave Basis Set. *Comput Mater Sci* **1996**, *6* (1), 15–50. https://doi.org/10.1016/0927-0256(96)00008-0.

(18) Blöchl, P. E. Projector Augmented-Wave Method. *Phys Rev B* **1994**, *50* (24), 17953–17979. https://doi.org/10.1103/PhysRevB.50.17953.

(19) Kresse, G.; Joubert, D. From Ultrasoft Pseudopotentials to the Projector Augmented-Wave Method. *Phys Rev B* **1999**, *59* (3), 1758–1775. https://doi.org/10.1103/PhysRevB.59.1758.

(20) Zhou, F.; Cococcioni, M.; Marianetti, C. A.; Morgan, D.; Ceder, G. First-Principles Prediction of Redox Potentials in Transition-Metal Compounds with LDA + U. *Phys Rev B* **2004**, *70* (23), 235121-1-235121–235128. https://doi.org/10.1103/PhysRevB.70.235121.

(21) Anisimov, V. I.; Zaanen, J.; Andersen, O. K. Band Theory and Mott Insulators: Hubbard U Instead of Stoner I. *Phys Rev B* **1991**, *44* (3), 943–954. https://doi.org/10.1103/PhysRevB.44.943.

(22) Anisimov, V. I.; Solovyev, I. v.; Korotin, M. A.; Czyyk, M. T.; Sawatzky, G. A. Density-Functional Theory and NiO Photoemission Spectra. *Phys Rev B* **1993**, *48* (23), 16929–16934. https://doi.org/10.1103/PhysRevB.48.16929.

(23) Liechtenstein, A. I.; Anisimov, V. I.; Zaanen, J. Density-Functional Theory and Strong Interactions: Orbital Ordering in Mott-Hubbard Insulators. *Phys Rev B* **1995**, *52* (8), R5467. https://doi.org/10.1103/PhysRevB.52.R5467.

(24) Li, X.; Ma, X.; Su, D.; Liu, L.; Chisnell, R.; Ong, S. P.; Chen, H.; Toumar, A.; Idrobo, J.-C.; Lei, Y.; Bai, J.; Wang, F.; Lynn, J. W.; Lee, Y. S.; Ceder, G. Direct Visualization of the Jahn–Teller Effect Coupled to Na Ordering in Na 5/8 MnO 2. *Nat Mater* **2014**, *13* (6), 586–592. https://doi.org/10.1038/nmat3964.

(25) Dovesi, R.; Erba, A.; Orlando, R.; Zicovich-Wilson, C. M.; Civalleri, B.; Maschio, L.; Rérat, M.; Casassa, S.; Baima, J.; Salustro, S.; Kirtman, B. Quantum-Mechanical Condensed Matter Simulations with CRYSTAL. *Wiley Interdiscip Rev Comput Mol Sci* **2018**, *8* (4), e1360. https://doi.org/10.1002/wcms.1360.

(26) Becke, A. D. Density-functional Thermochemistry. III. The Role of Exact Exchange. *J Chem Phys* **1993**, *98* (7), 5648–5652. https://doi.org/10.1063/1.464913.

(27) Lee, C.; Yang, W.; Parr, R. G. Development of the Colle-Salvetti Correlation-Energy Formula into a Functional of the Electron Density. *Phys Rev B* **1988**, *37* (2), 785–789. https://doi.org/10.1103/PhysRevB.37.785.

(28) Catti, M.; Sandrone, G.; Dovesi, R. Periodic Unrestricted Hartree-Fock Study of Corundumlike Ti2O3 and V2O3. *Phys Rev B* **1997**, *55* (24), 16122–16131. https://doi.org/10.1103/PhysRevB.55.16122.

(29) Catti, M.; Valerio, G.; Dovesi, R.; Causà, M. Quantum-Mechanical Calculation of the Solid-State Equilibrium MgO+α-Al2O3 ⇄ MgAl2O4 (Spinel) versus Pressure. *Phys Rev B* **1994**, *49* (20), 14179–14187. https://doi.org/10.1103/PhysRevB.49.14179.

(30) Catti, M.; Sandrone, G.; Valerio, G.; Dovesi, R. Electronic, Magnetic and Crystal Structure of Cr2O3 by Theoretical Methods. *Journal of Physics and Chemistry of Solids* **1996**, *57* (11), 1735–1741. https://doi.org/10.1016/0022-3697(96)00034-0.

(31) Schäfer, A.; Horn, H.; Ahlrichs, R. Fully Optimized Contracted Gaussian Basis Sets for Atoms Li to Kr. *J Chem Phys* **1992**, *97* (4), 2571–2577. https://doi.org/10.1063/1.463096.

(32) Kutzelnigg, W.; Fleischer, U.; Schindler, M. The IGLO-Method: Ab-Initio Calculation and Interpretation of NMR Chemical Shifts and Magnetic Susceptibilities; Springer, Berlin, Heidelberg, 1990; pp 165–262. https://doi.org/10.1007/978-3-642-75932-1_3.

(33) Lee, J.; Seymour, I. D.; Pell, A. J.; Dutton, S. E.; Grey, C. P. A Systematic Study of 25 Mg NMR in Paramagnetic Transition Metal Oxides: Applications to Mg-Ion Battery Materials. *Physical Chemistry Chemical Physics* **2017**, *19* (1), 613–625. https://doi.org/10.1039/C6CP06338A.

(34) Henkelman, G.; Uberuaga, B. P.; Jónsson, H. Climbing Image Nudged Elastic Band Method for Finding Saddle Points and Minimum Energy Paths. *Journal of Chemical Physics* **2000**, *113* (22), 9901–9904. https://doi.org/10.1063/1.1329672.

(35) Henkelman Group. VTST Tools 3.1. Austin, TX.

(36) Bassey, E. N.; Reeves, P. J.; Jones, M. A.; Lee, J.; Seymour, I. D.; Cibin, G.; Grey, C. P. Structural Origins of Voltage Hysteresis in the Na-Ion Cathode P2-Na0.67[Mg0.28Mn0.72]O2: A Combined Spectroscopic and DFT Study. *Chemistry of Materials Submitted*.

(37) Van Der Ven, A.; Deng, Z.; Banerjee, S.; Ong, S. P. Rechargeable Alkali-Ion Battery Materials: Theory and Computation. *Chemical Reviews*. American Chemical Society July 22, 2020, pp 6977–7019. https://doi.org/10.1021/acs.chemrev.9b00601.

(38) Urban, A.; Seo, D. H.; Ceder, G. Computational Understanding of Li-Ion Batteries. *npj Computational Materials*. Nature Publishing Group March 18, 2016, pp 1–13. https://doi.org/10.1038/npjcompumats.2016.2.

(39) Smart, L. E.; Moore, E. A. *Solid State Chemistry: An Introduction*, 4th ed.; CRC Press, 2012.

(40) Mcconnell, H. M. Reaction Rates by Nuclear Magnetic Resonance. *J Chem Phys* **1958**, *28*, 430–431. https://doi.org/10.1063/1.1744152.

(41) Bielecki, A.; Burum, D. P. Temperature Dependence Of207Pb MAS Spectra of Solid Lead Nitrate. An Accurate, Sensitive Thermometer for Variable-Temperature MAS. *J Magn Reson A* **1995**, *116* (2), 215–220. https://doi.org/10.1006/jmra.1995.0010.

(42) Thurber, K. R.; Tycko, R. Measurement of Sample Temperatures under Magic-Angle Spinning from the Chemical Shift and Spin-Lattice Relaxation Rate of 79Br in KBr Powder. *Journal of Magnetic Resonance* **2009**, *196* (1), 84–87. https://doi.org/10.1016/J.JMR.2008.09.019.

(43) Keen, D. A. Total Scattering and the Pair Distribution Function in Crystallography. *Crystallogr Rev* **2020**, *2020* (3), 141–199. https://doi.org/10.1080/0889311X.2020.1797708.

(44) Goodwin, A. L.; Tucker, M. G.; Dove, M. T.; Keen, D. A. Magnetic Structure of MnO at 10 K from Total Neutron Scattering Data. **2006**. https://doi.org/10.1103/PhysRevLett.96.047209.

(45) Blundell, S. J. *Magnetism in Condensed Matter*, Second.; Oxford University Press: Oxford, 2014.
